# Direct coupling and protective activation of DRP1 by the DNA-PKcs inhibitor KU-57788 synergizes with ferroptosis in anaplastic thyroid cancer cells

**DOI:** 10.1038/s41419-026-08595-3

**Published:** 2026-04-28

**Authors:** Lingling Ding, Changtian Yin, Yehao Guo, Qiang Geng, Wanwan He, Yawen Guo, Jinpeng Wen, Aoni Zhou, Jieyu Luo, Xinxin Ren, Jiajie Xu, Renhao Ou, Ruonan Jia, Jiaxin Tian, Yuchen Wang, Yefeng Cai, Wenzhen Wang, Haifeng Xu, Lei Zhu, Minghua Ge, Guowan Zheng, Chuanming Zheng

**Affiliations:** 1 Otolaryngology & Head and Neck Center, Cancer Center, Department of Head and Neck Surgery, Zhejiang Provincial People’s Hospital (Affiliated People’s Hospital), Hangzhou Medical College, Hangzhou, Zhejiang China; 2 Department of Otolaryngology-Head and Neck Surgery, The Affiliated Lihuili Hospital, Ningbo University, Ningbo, Zhejiang China; 3 Zhejiang Key Laboratory of Precision Medicine Research on Head & Neck Cancer, Hangzhou, China, Hangzhou, Zhejiang China; 4 Zhejiang Provincial Clinical Research Center for Head & Neck Cancer, Hangzhou, Zhejiang China; 5 Postgraduate Training Base Alliance of Wenzhou Medical University (Zhejiang Provincial People’s Hospital), Wenzhou, Zhejiang China; 6 The Second School of Clinical Medicine, Zhejiang Chinese Medical University, Hangzhou, Zhejiang China; 7 The Second School of Clinical Medicine, Hangzhou Normal University, Hangzhou, Zhejiang China; 8 Department of Thyroid Surgery, National Key Clinical Specialty (General Surgery), The First Affiliated Hospital of Wenzhou Medical University, Wenzhou, Zhejiang China; 9 Zhejiang University School of Medicine, Zhejiang University, Hangzhou, Zhejiang China; 10 Key Laboratory of Bioorganic Synthesis of Zhejiang Province, College of Biotechnology and Bioengineering, Zhejiang University of Technology, Hangzhou, China; 11 Department of Clinical Laboratory, The Children’s Hospital, Zhejiang University School of Medicine, National Clinical Research Center for Child Health, Hangzhou, Zhejiang China; 12 Department of Thyroid Surgery, The Fifth Hospital Affiliated to Wenzhou Medical University, Lishui Central Hospital, Lishui, Zhejiang China

**Keywords:** Cancer, Medical research

## Abstract

Anaplastic thyroid carcinoma (ATC) is one of the most aggressive and lethal malignancies, with limited treatment options and poor clinical outcomes. KU-57788, a selective inhibitor of DNA-dependent protein kinase catalytic subunit (DNA-PKcs), has shown promise in cancer therapy when combined with radiotherapy and chemotherapy. However, its therapeutic potential and underlying mechanisms in ATC remain unclear. In this study, we demonstrate that KU-57788 exerts potent anti-ATC activity both in vitro and in vivo by inducing DNA damage and triggering mitotic catastrophe. Unexpectedly, we identify a novel mechanism whereby KU-57788 directly binds to and activates dynamin-related protein 1 (DRP1), leading to excessive mitochondrial fission and fragmentation. This process is accompanied by the protective activation of the NRF2/SLC7A11/GSH axis, which mitigates the cytotoxic effects of KU-57788. Notably, pharmacological induction of ferroptosis or cystine depletion effectively synergizes with ATC cells to KU-57788, overcoming resistance and promoting ferroptosis. Collectively, our findings highlight the therapeutic potential of KU-57788 in ATC while revealing an intrinsic resistance mechanism mediated by DRP1 activation and the potential involvement of the NRF2/SLC7A11/GSH axis. More importantly, we provide strong evidence that combining KU-57788 with ferroptosis inducers significantly enhances its anticancer efficacy, offering a promising therapeutic strategy for ATC.

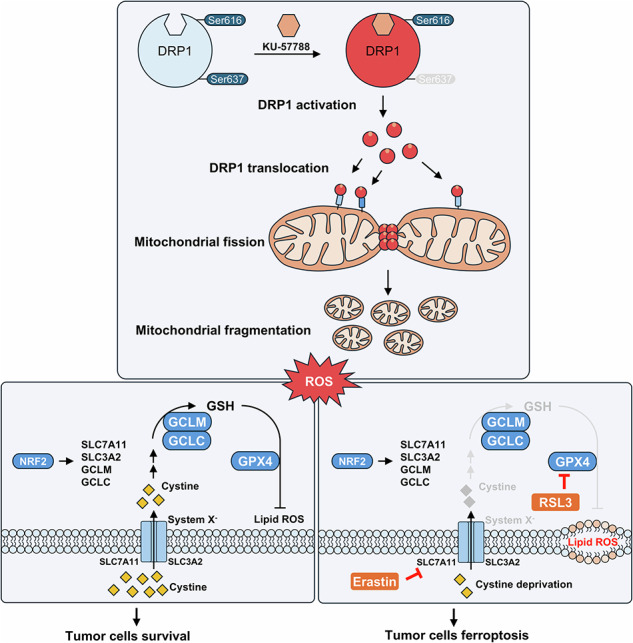

## Introduction

Anaplastic thyroid carcinoma (ATC) is among the most aggressive malignancies in humans. Although ATC accounts for less than 2% of all thyroid cancers, it is responsible for 14–39% of thyroid cancer-related deaths. The prognosis for ATC is exceptionally poor, with a 5-year survival rate typically below 10%. The median overall survival (OS) from diagnosis is approximately four months, and the disease-specific mortality rate approaches 100% [1]. This grim outlook is largely attributed to the fact that ATC is often diagnosed at an advanced stage, by which point it has already metastasized to cervical lymph nodes and distant organs. Moreover, ATC exhibits a poor response to conventional treatments like surgery, radiotherapy, and chemotherapy, and shows limited efficacy with emerging therapies, including targeted therapies and immunotherapy [2]. Therefore, there is an urgent need to identify novel therapeutic strategies, targets, and candidate drugs to improve the survival rates and quality of life for patients with ATC.

Targeted therapy has emerged as a cornerstone of cancer treatment; however, the widespread emergence of drug resistance remains a major obstacle to its effectiveness. The intricate genetic landscape and heterogeneity of tumor cells complicate the ability of targeted therapies to uniformly address all cancer subtypes. Even in cases where tumors initially respond to targeted therapy, tumor cells can activate compensatory signaling pathways that bypass the inhibitory effects of the drug, ultimately driving tumor relapse and progression [3, 4]. For instance, treatment with MEK inhibitors can lead to the activation of the IL6/JAK/STAT3 signaling pathway in tumor cells, enabling them to sustain survival and proliferation despite the inhibitory effects of the drug [5]. As our understanding of targeted therapies advances, it has become increasingly clear that many targeted drugs do not solely interact with a single, defined target. Instead, numerous small molecule inhibitors exert their anti-cancer effects through off-target interactions, with their anti-tumor activity either independent of, or partially reliant on, their intended targets [6–8]. These off-target effects, which may involve previously unrecognized or alternative targets, can offer potential therapeutic benefits but also pose risks of adverse side effects and the development of resistance [9, 10]. It is worth noting that these off-target effects may also become significant resistance factors promoting tumor progression, as targeted drugs might activate these targets to protect tumor cells [11]. Therefore, this resistance mechanism deserves our attention, as studying this phenomenon is crucial for enhancing the anti-tumor efficacy of targeted therapies and guiding drug development.

DNA-dependent protein kinase catalytic subunit (DNA-PKcs), encoded by the PRKDC gene, primarily participates in DNA damage repair through the non-homologous end-joining (NHEJ) pathway, which is essential for repairing double-strand DNA breaks and maintaining genomic integrity [12]. DNA-PKcs is abnormally activated in various tumors, playing a key role in repairing DNA damage caused by replication stress, thus supporting the high proliferation rate of tumor cells [13]. Genetic alterations in DNA-PKcs, such as point mutations and copy number amplifications, are common across multiple cancer types, particularly in uterine corpus endometrial carcinoma, uterine cancer, skin melanoma, stomach adenocarcinoma, liver hepatocellular carcinoma, lung adenocarcinoma, and colorectal adenocarcinoma [14]. Therefore, DNA-PKcs is considered a negative prognostic factor in cancer. Our current findings reveal that DNA-PKcs is significantly elevated in ATC tissues and serves as a negative prognostic factor for thyroid cancer (Fig. S1). Knockdown of PRKDC significantly suppresses ATC cell proliferation (Fig. S2). Currently, the small-molecule compound KU-57788 (also named NU7441) is a specific inhibitor of DNA-PKcs and is commonly used as a research tool for targeting DNA-PKcs function [15]. Although KU-57788 can be used independently to target DNA-PKcs for its anti-tumor effect, it is more commonly applied to synergize with tumors to radiotherapy and chemotherapy drugs [16, 17]. Additionally, KU-57788 can inhibit Brd4, PI3K, and mTOR activities [18], though the effects of these actions remain unclear. These findings encourage further investigation into the therapeutic potential of targeting DNA-PKcs in ATC treatment.

In the present study, we demonstrated that the DNA-PKcs inhibitor KU-57788 significantly inhibits ATC cell growth in vitro and in vivo, associated with cell cycle arrest and mitotic catastrophe. KU-57788 induces excessive mitochondrial fission and fragmentation by directly binding to and activating DRP1, independent of its primary target DNA-PKcs. This mitochondrial dysfunction is subsequently accompanied by the activation of the NRF2/SLC7A11/GSH axis in ATC cells, triggering a protective response that mitigates KU-57788-induced cytotoxicity. Notably, pharmacological induction of ferroptosis or cystine depletion effectively enhances the synergistic effect of ATC cells to KU-57788, promoting ferroptosis of ATC cells. These findings uncover a previously unrecognized role of KU-57788 as a DRP1 agonist, provide new insights into its role as a synergistic promoter of ferroptosis and offer a novel perspective for its application in ATC treatment.

## Results

### KU-57788 induces mitotic catastrophe and cellular senescence in ATC cells

**Fig. 1:**
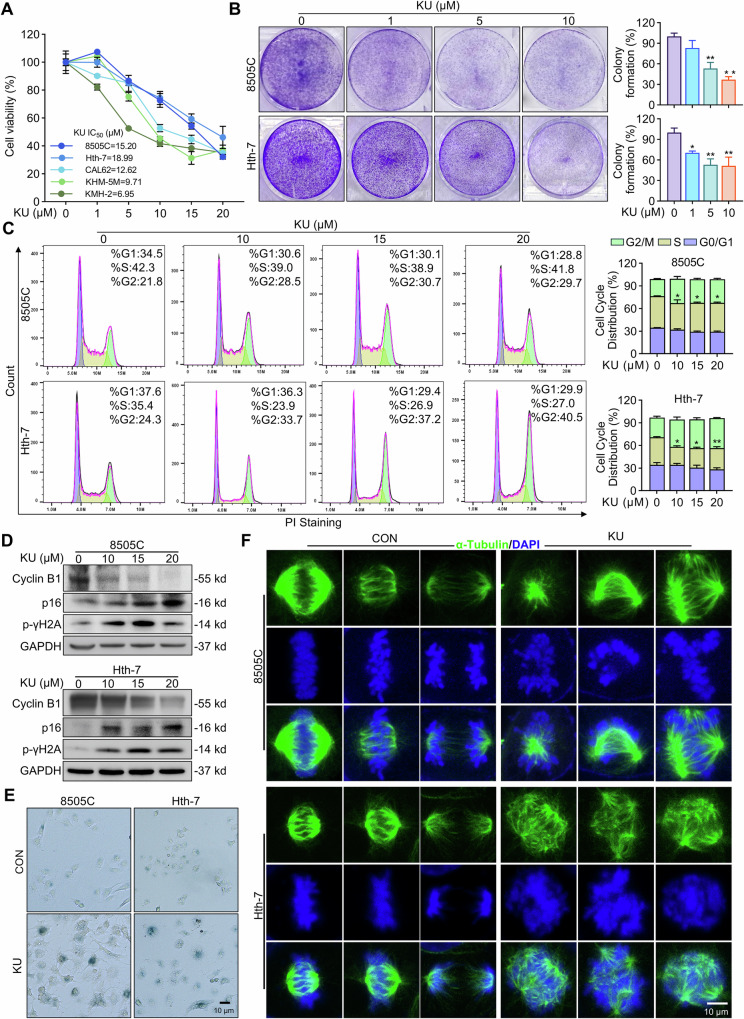
KU-57788 inhibited cell viability, colony formation, and the cell cycle, induced cellular senescence, and caused mitotic catastrophe in human ATC cells. **A** CCK-8 assay was used to determine the cell viability of different concentrations of KU-57788 (0, 1, 5, 10, 15, and 20 μM) in 8505C, Hth-7, CAL-62, KHM-5M, and KMH-2 cells after 24 h, respectively. IC_50_ values were calculated from non-linear regression plots using GraphPad 9.0. **B** Quantitative and qualitative analysis of clone formation in 8505 C and Hth-7 (seeded at 2 × 10⁴ cells/well) cells at the indicated concentrations of KU-57788 (0, 1, 5, and 10 μM). **C** Cell cycle phase distribution of KU-57788-treated 8505C and Hth-7 cells (0, 10, 15, and 20 μM) for 12 h was determined by flow cytometry. **D** Western blot analysis was used to measure the protein levels of Cyclin B1, P16, and p-γH2A in 8505C and Hth-7 cells after treatment with KU-57788 (0, 10, 15, and 20 μM) for 24 hours. **E** Cellular senescence analysis of 8505C and Hth-7 cells treated with 10 μM KU-57788 for 72 h was performed using SA-β-Gal staining. **F** Chromatin of cells in the cytokinesis phase, after KU-57788 (10 μM) administration for 24 hours, was observed by confocal microscopy. Values are presented as mean ± SD for *n* = 3, analyzed by one-way ANOVA using the Holm-Sidak method. ns, *p < 0.05, **p < 0.01, ns, *p* > 0.05.

The abnormal upregulation of DNA-PKcs in ATC tissues suggests that DNA-PKcs might be a critical protein for ATC cell division and proliferation (Fig. S1). Using small RNA interference (siRNA), we demonstrated that DNA-PKcs knockdown significantly inhibited the proliferation and colony formation of ATC cell lines 8505C and Hth-7 (Fig. S2 and Fig. S3A), underscoring its critical role in driving ATC progression. Subsequently, we evaluated the inhibitory effects of the DNA-PKcs inhibitor KU-57788 in five ATC cell lines using the CCK8 assay. As shown in Fig. 1A, KU-57788 inhibited ATC cell viability in a dose-dependent manner, with IC_50_ values of 15.20 μM for 8505C, 18.99 μM for Hth-7, 12.62 μM for CAL-62, 9.71 μM for KHM-5M, and 6.95 μM for KMH-2, further supporting the potent anti-tumor activity and effective DNA-PKcs inhibition of KU-57788. Additionally, KU-57788 significantly inhibited colony formation in 8505C and Hth-7 cells in a dose-dependent manner (Fig. 1B), providing strong evidence of the growth arrest. We next assessed the cell cycle distribution in KU-57788-treated 8505C and Hth-7 cells using flow cytometry. The results showed that KU-57788 induced a dose-dependent G2/M phase arrest (Fig. 1C). Western blot analysis further confirmed the downregulation of the G2/M phase-associated protein cyclin B1 (Fig. 1D and Fig. S3B).

Surprisingly, further analysis using a flow cytometry apoptosis kit revealed that although KU-57788 increased the number of PI/AV-positive cells in ATC cells (Fig. S4), the majority of the cells remained viable. This observation was inconsistent with the reduction in cell viability (Fig. 1A). Notably, KU-57788-treated cells remained adherent, adopted a more spindle-shaped and elongated morphology, and exhibited an increase in cell size with prolonged treatment (Fig. S5A). These changes suggested that KU-57788 may ultimately induce senescence in ATC cells. Indeed, a significant increase in β-galactosidase-positive cells, exhibiting blue or green staining, was observed in KU-57788-treated 8505C and Hth-7 cells (Fig. 1E), along with a notable upregulation of senescence-associated markers p16 and γH2A (Fig. 1D and Fig. S3B). These results collectively demonstrate that KU-57788 prominently induces senescence in ATC cells.

DNA-PKcs is a critical component of the DNA non-homologous end joining (NHEJ) repair pathway, essential for maintaining DNA integrity in tumor cells. Previous studies have shown that inhibition of DNA-PKcs activity can lead to mitotic abnormalities in tumor cells, resulting in mitotic catastrophe [19]. This provides a potential explanation for why KU-57788 induces senescence in ATC cells. Using microtubule staining, we observed that KU-57788 treatment caused hallmark features of mitotic catastrophe in ATC cells, including chromosomal misalignment and multipolar spindle formation during cell division (Fig. 1F). These findings suggest that KU-57788 induces mitotic catastrophe in ATC cells, which may be a key mechanism underlying their senescence.

### KU-57788 induces mitophagy in ATC cells independent of cell viability effects

**Fig. 2:**
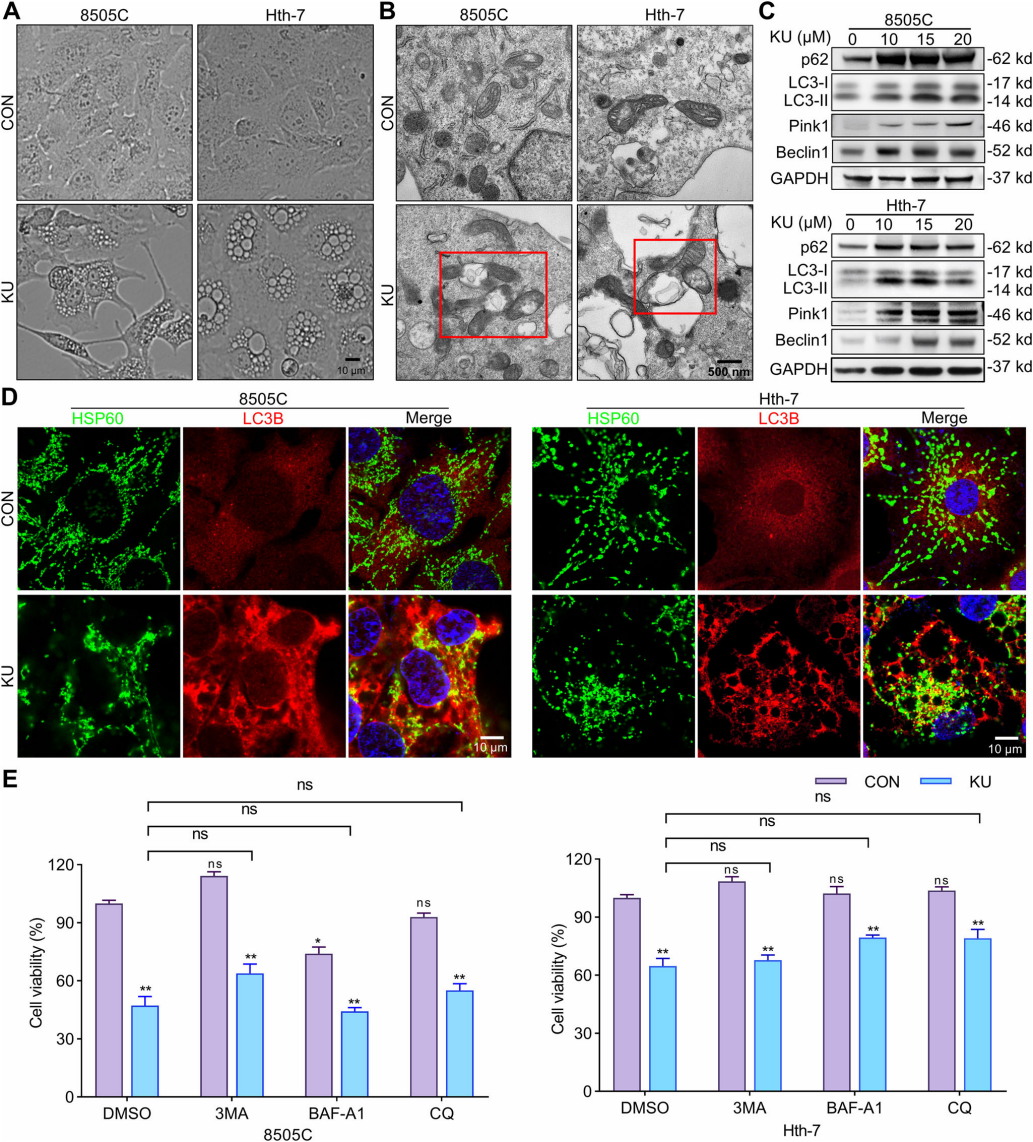
KU-57788 activates ATC mitophagy. **A** Characteristic morphology of 8505C and Hth-7 cells induced by KU-57788 (0 and 10 μM for 24 h) by using a phase-contrast optical microscope (Evos M7000). **B** Transmission electron microscopy was used to observe mitochondrial morphological changes in 8505C and Hth-7 cells treated with KU-57788 (0 and 10 μM for 24 h). **C** Western blot was used to measure the protein levels of p62, LC3, Pink1, Beclin1, and GAPDH in 8505C and Hth-7 cells, after KU-57788 (0, 10, 15 and 20 μM for 24 h). **D** Confocal microscopy was used to observe changes in HSP60 and LC3B in 8505C and Hth-7 cells treated with KU-57788 (0 and 10 μM for 24 h). **E** In 8505C and Hth-7 cells, KU-57788 (0 and 20 μM) is used in combination with 3-MA (3 mM, pre-treated for 2 h), BafA1 (100 nM, pre-treated for 2 h) and CQ (10 μM, pre-treated for 2 h) for 24 h, cell viability was measured by the CCK-8 assay, respectively. Statistical comparisons were made between all drug treatment groups and the control group. Data are shown as mean ± SD for *n* = 3, analyzed by one-way ANOVA using the Holm-Sidak method. *p < 0.05, **p < 0.01, ns, *p* > 0.05.

Cellular senescence is often associated with the loss of mitochondrial function and the accumulation of damaged mitochondria [20, 21]. Mitophagy serves as the primary mechanism for clearing damaged mitochondria [22]. In KU-57788-treated ATC cells, 8505C and Hth-7, numerous vacuoles were observed under bright-field phase-contrast microscopy (Fig. 2A and Fig. S5B). To investigate further, transmission electron microscopy analysis was conducted, revealing altered mitochondrial morphology and damaged mitochondria (Fig. 2B and Fig. S5C). These damaged mitochondria were surrounded by vacuoles and incorporated within them (Fig. 2B and Fig. S5C), suggesting that KU-57788 may induce mitophagy. Thus, we investigated the expression of mitophagy-related proteins using western blot analysis and found that KU-57788 significantly increased the levels of p62, LC3B, Pink1 and Beclin1, and decrease the level of Parkin and BNIP3 in ATC cells (Fig. 2C, Fig. S6, Fig. S3C, D). This indicates that KU-57788 causes mitochondrial damage in ATC cells, leading to the induction of selective mitophagy, potentially through a Pink1-dependent mechanism. Immunofluorescence staining demonstrated colocalization of the autophagy marker protein LC3B (Red) with the mitochondria marker protein HSP60 (Green) (Fig. 2D and Fig. S7). Notably, knocking-down DNA-PKcs had minimal impact on the expression of mitophagy-related proteins p62, LC3B, Pink1, and Beclin1 (Fig. S3E, F, Fig. S8A, B), and failed to induce vacuole formation (Fig. S8C). KU-57788 treatment led to increased expression of LC3, p62, Pink1 and Beclin1 (Fig. 2C and Fig. S3C), and decreased expression of Parkin and BNIP3 (Fig. S3D and Fig. S6), essentially irrespective of DNA-PKcs knockdown (Fig S3F, Fig. S8B). These results suggesting that KU57788-induced mitophagy was likely independent of DNA-PKcs inhibition.

To clarify the role of KU-57788-induced mitophagy in its anti-ATC effects, we employed several inhibitors: the autophagy initiation inhibitor 3-Methyladenine (3-MA), the autophagolysosome inhibitor chloroquine (CQ), and the V-ATPase inhibitor bafilomycin A1 (BafA1). The results demonstrated that none of these inhibitors effectively reversed or enhanced the growth-inhibitory effects of KU-57788 on ATC cells (Fig. 2E). Besides, we utilized 3-MA, BafA1, and CQ, in combination with KU-57788 to treat both 8505C and Hth-7 cells. Western blot analysis was performed to measure the expression levels of autophagy marker LC3 II to assess the inhibitory effects of these treatments on autophagy. Our findings demonstrated that co-treatment with KU-57788 and the late-stage autophagy inhibitors (CQ or Baf-A1) resulted in enhanced accumulation of LC3-II induced by KU-57788. Conversely, the early-stage autophagy inhibitor 3-MA partially attenuated KU-mediated LC3-II activation. Together, these complementary lines of evidence confirm that KU-57788 activates autophagic flux, and this pro-autophagic effect can be partially reversed by 3-MA (Fig. S9, Fig. S3H). These findings suggest that the mitophagy induced by KU-57788 may be a stress response to mitochondrial damage, rather than a direct mechanism contributing to its anti-ATC activity.

### KU-57788 promotes mitochondrial fission

**Fig. 3:**
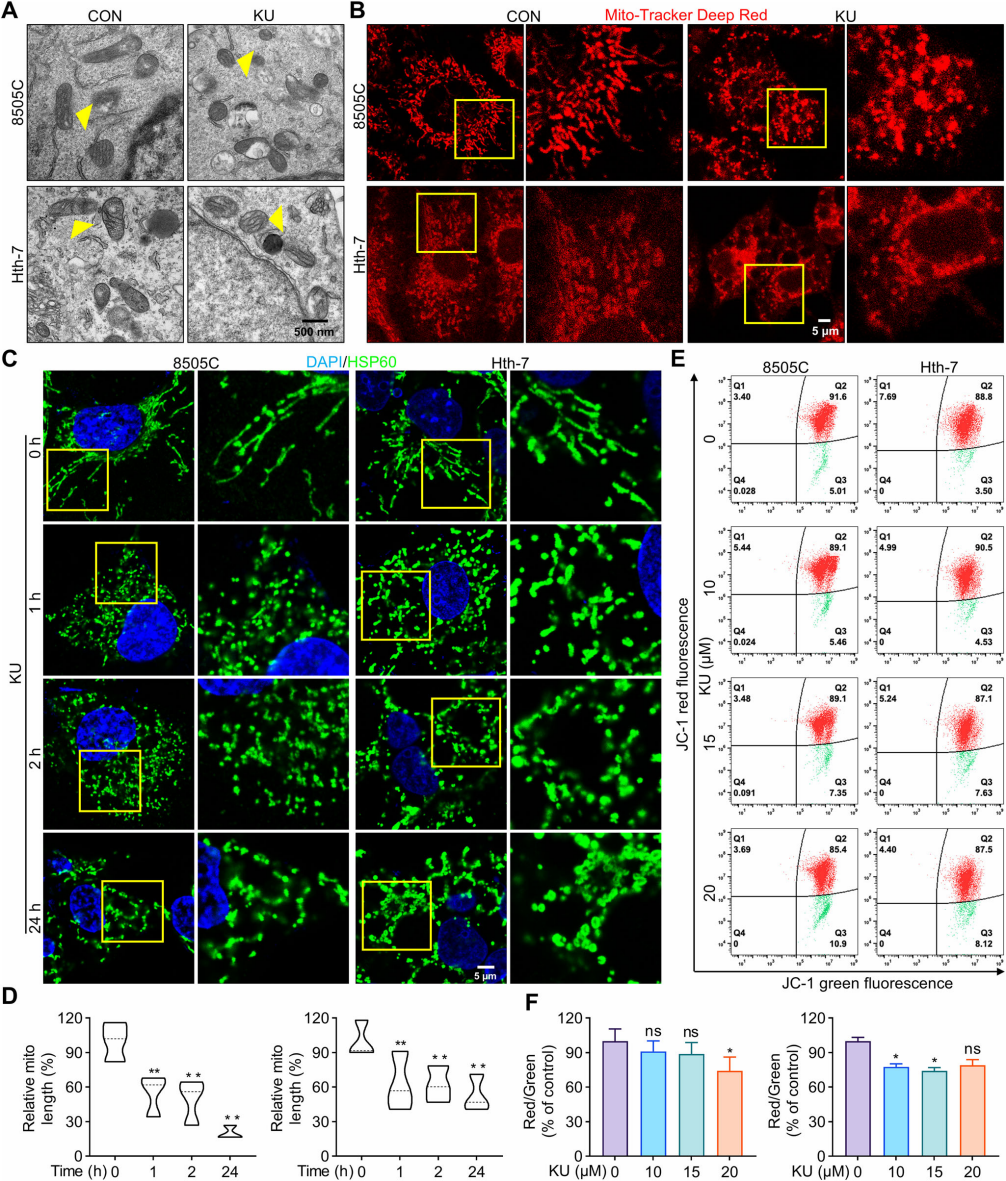
KU-57788 promotes mitochondrial fission. **A** Transmission electron microscopy was used to observe mitochondrial morphological changes in 8505C and Hth-7 cells treated with KU-57788 (0 and 10 μM for 24 h). **B** Confocal laser microscopy (Leica STED, Germany) was used to observe mitochondrial morphological changes in 8505C and Hth-7 cells treated with KU-57788 (0 and 10 μM for 24 h), with MitoTracker Deep Red FM. **C** Confocal laser microscopy was used to observe mitochondrial morphological changes in 8505C and Hth-7 cells treated with KU-57788 (0, 10 μM for 0, 1, 2, and 24 h) after incubation with HSP60 antibody by immunofluorescence. **D** Quantification of mitochondrial length (mito length) from Fig. 3C. **E** Flow cytometry was used to determine the mitochondrial membrane potential indicated by the distribution ratios of red and green cell populations in KU-57788-treated ATC cells stained by JC-1 (8505 C and Hth-7, 0, 10, 15, and 20 μM of KU-57788 for 24 h). **F** Quantitative analysis of mitochondrial membrane potential based on the ratio of red to green fluorescence intensity in Fig. 3E. Data are shown as mean ± SD for *n* = 3, analyzed by one-way ANOVA using the Holm-Sidak method. *p < 0.05, **p < 0.01, ns, *p* > 0.05.

While confirming that KU-57788 induces mitochondrial morphological changes and mitophagy in ATC cells, we further observed under transmission electron microscopy that KU-57788 treatment significantly altered the mitochondrial morphology of ATC cell lines 8505C and Hth-7. The mitochondria became shorter and rounder, with their internal structures appearing more irregular and incomplete (Fig. 3A), which suggested that KU-57788 may promote mitochondrial fission in ATC cells. Therefore, we stained mitochondria using the mitochondrial probe MitoTracker® Deep Red FM and conducted confocal microscopy analysis. Obviously, following KU-57788 treatment, mitochondria in both 8505C and Hth-7 cells became irregular, with smaller spherical or short rod-like shapes, whereas mitochondria predominantly exhibited elongated or oval shapes in the control group (Fig. 3B). Consistent results were observed in HSP60 fluorescence staining of ATC cells treated early with KU-57788, characterized by smaller, more spherical mitochondria and an increased mitochondria count (Fig. 3C, Fig. 3D and Fig S10).

During mitochondrial fission, the proton motive force may be unevenly distributed across the resulting mitochondria, leading to instability in mitochondrial membrane potential. Smaller mitochondria formed after fission are more prone to damage, often exhibiting reduced membrane potential [23, 24]. Using the mitochondrial membrane potential probe JC-1, we found that KU-57788 caused a modest reduction in mitochondrial membrane potential (Fig. 3E and Fig. 3F). These findings support the notion that KU-57788 promotes mitochondrial fission, leading to mitochondrial instability.

### KU-57788 promotes mitochondrial fission by inhibiting Drp1-S637 phosphorylation

**Fig. 4:**
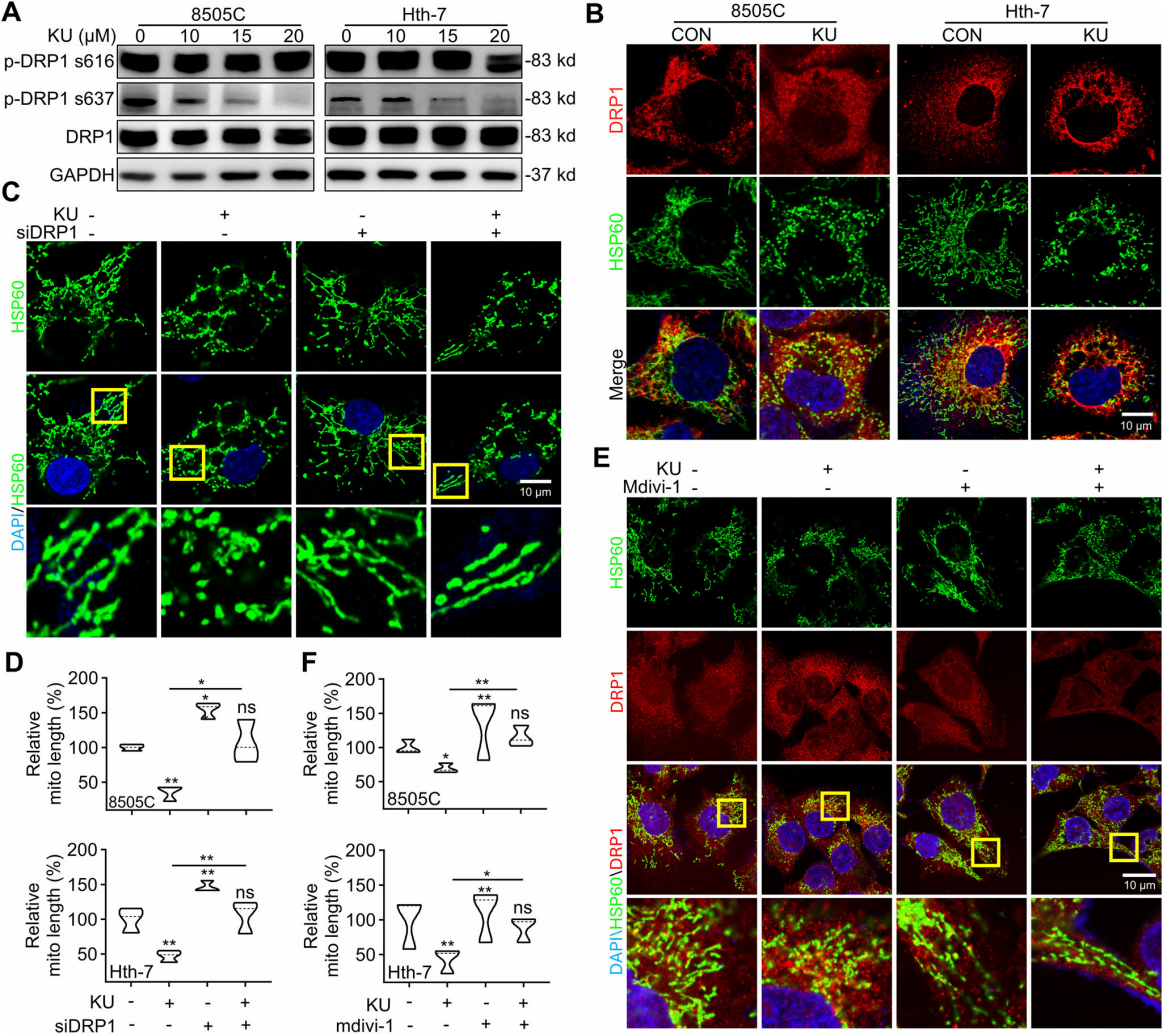
KU-57788 promotes mitochondrial fission through activating DRP1. **A** Western blot was used to measure p-DRP1 s616, p-DRP1 s637, DRP1, and GAPDH protein levels in 8505C and Hth-7 cells, after KU-57788 (0, 10, 15 and 20 μM for 24 h) respectively. **B** Confocal laser was used to observe mitochondrial morphological changes in 8505C and Hth-7 cells treated with KU-57788 (0 and 10 μM for 0 h, 1 h, 2 h and 24 h), after incubated with HSP60 and DRP1 antibody by immunofluorescence. **C** After knock-down DRP1 by siRNA, confocal laser was used to observe mitochondrial morphological changes in 8505C, treated with KU-57788 (0 and 10 μM for 24 h), where mitochondrial were stained by HSP60 antibody. **D** Quantification of mitochondrial length (mito length) of C. **E** Confocal laser was used to observe mitochondrial morphological changes in 8505C, treated with KU-57788 (0 and 10 μM for 24 h) and a DRP1 inhibitor Mdivi-1 (0 and 10 μM for 24 h), where mitochondria were stained by HSP60 and DRP1 antibody. **F** Quantification of mitochondrial length of E. Data are shown as mean ± SD for *n* = 3, analyzed by one-way ANOVA using the Holm-Sidak method. *p < 0.05, **p < 0.01, ns, *p* > 0.05.

Previously, our research demonstrated that Ruxolitinib inhibits the JAK-STAT3 signaling pathway, resulting in the downregulation of dynamin-related protein 1 (DRP1) expression [25]. This reduction in DRP1 disrupts mitochondrial fission, thereby inducing apoptosis and pyroptosis in ATC cells [25, 26]. These findings highlight DRP1 as a critical regulator of mitochondrial fission in ATC cells. Building on this knowledge, we investigated whether DRP1 plays a pivotal role in mediating the rapid and sustained mitochondrial fission observed in ATC cells treatment with KU-57788. Our analysis revealed that KU-57788 did not significantly alter DRP1 mRNA or protein expression levels (Fig. 4A and Fig. S3I, J, Fig. S11A, B). This prompted us to examine DRP1 phosphorylation, as phosphorylation at Ser616 and dephosphorylation at Ser637 are known to be critical for DRP1 activation and its translocation from the cytosol to mitochondria, initiating mitochondrial fission [27, 28]. Western blot assays showed that while KU-57788 did not affect DRP1 Ser616 phosphorylation, it markedly and time- and dose-dependently reduced DRP1-Ser637 phosphorylation in a rapid and sustained manner (Fig. 4A Fig. S3I, J and Fig. S11A). Notably, similar to the lack of effect on mitophagy or mitochondrial morphology observed with DNA-PKcs knockdown (Fig. S3E, F and Fig. S8A, B), silencing DNA-PKcs also did not alter the expression or phosphorylation status of DRP1 (Fig. S3G and Fig. S8D). These findings further confirm that KU-57788-induced DRP1-dependent mitochondrial fission occurs independently of DNA-PKcs. Furthermore, these results suggest that KU-57788 modulates DRP1 activity through dynamic regulation of its phosphorylation. This conclusion was further supported by HSP60 and DRP1 immunofluorescence co-staining, which demonstrated that KU-57788 treatment rapidly induced the translocation of DRP1 from the cytoplasm to mitochondria in ATC cell lines 8505C and Hth-7, coinciding with mitochondrial fission (Fig. 3C, D, Fig. 4B and Fig. S11F). Moreover, the application of DRP1 knockdown via siRNA (Fig. S11C) or a DRP1 inhibitor (mdivi-1) significantly prevented KU-57788-induced mitochondrial fission, preserving the elongated mitochondrial morphology (Fig. 4C–F and Fig. S11D–G). Collectively, these results indicate that KU-57788 promotes sustained mitochondrial fission in ATC cells by activating DRP1 through phosphorylation modulation, highlighting a potential mechanism by which KU-57788 regulates mitochondrial dynamics in ATC cells.

### KU-57788 directly binds to DRP1 to promote mitochondrial fission

**Fig. 5:**
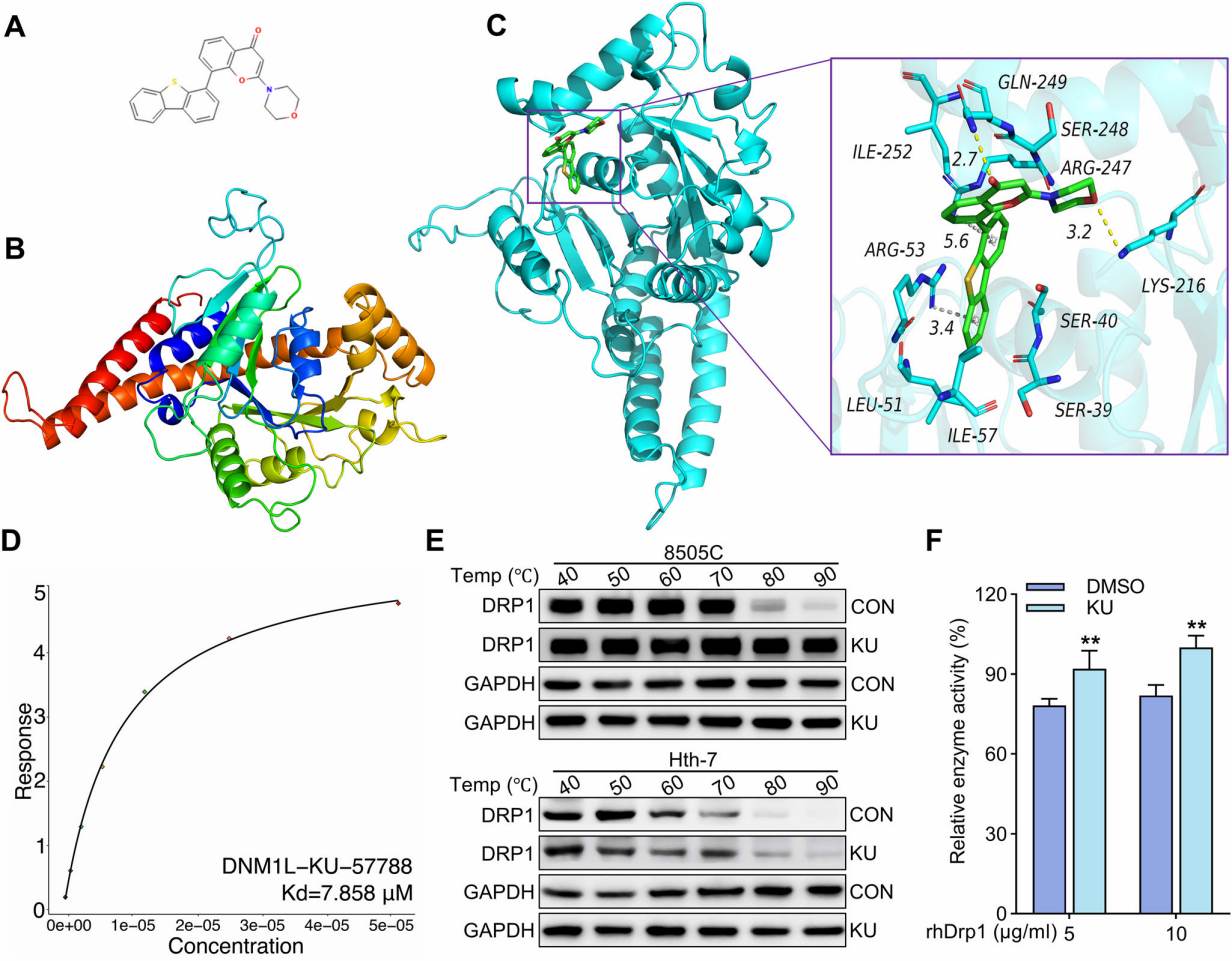
KU-57788 directly interacts DRP1 and activates DRP1. **A** Chemical formula of KU-57788. **B** Structural formula of DRP1. **C** Schematic Representation of the Interaction Pattern Between KU-57788 and DRP1. **D** Results of steady state model fitting of KU-57788 with target protein DRP1. **E** The enzyme activity of recombinant human DRP1 was assessed following in vitro treatment with KU-57788 (20 μM), using an ATPase/GTPase Activity Assay kit as per the manufacturer’s instructions. **F** CETSA-WB showed that KU-57788 (0 and 20 μM) increased the thermal stability of DRP1 protein in 8505C and Hth-7 cell lysates. Data are shown as mean ± SD for *n* = 3, analyzed by one-way ANOVA using the Holm-Sidak method. *p < 0.05, **p < 0.01, ns, *p* > 0.05.

We have demonstrated that KU-57788 regulates DRP1 activity and behavior by modulating DRP1 phosphorylation. Therefore, we first boldly investigated whether KU-57788 directly interacts with DRP1. The chemical structure of KU-57788 was retrieved from the PubChem database (Compound CID: 11327430, https://pubchem.ncbi.nlm.nih.gov/compound/11327430) (Fig. 5A) and structural information for DRP1 was obtained from the RCSB Protein Data Bank (PDB ID: 3W6P, https://www.rcsb.org/structure/3W6P) (Fig. 5B). These data were used to analyze KU-57788 as a ligand in subsequent interaction studies. Molecular docking was performed using the Glide module of Schrödinger’s Maestro software. The docking results revealed that KU-57788 binds to DRP1, forming hydrogen bonds with LYS-216 and GLN-249 and establishing π-cation interactions with ARG-53 and ARG-247 (Fig. 5C and Fig. S12). The docking binding energy was calculated to be −6.851 kcal/mol, indicating a potential interaction between KU-57788 and DRP1. To further validate this interaction, molecular dynamics simulations were carried out using the Gromacs software suite. The g-MMPBSA method was applied to calculate the binding free energy of the KU-57788-DRP1 complex, where KU-57788 served as the ligand and DRP1 as the receptor. The results showed an average binding free energy of −78.314 kcal/mol, supporting a strong and stable interaction between KU-57788 and DRP1.

To further validate the interaction between KU-57788 and DRP1, we employed surface plasmon resonance (SPR) assay to evaluate their binding and dissociation kinetics. Recombinant DRP1 protein was immobilized onto an NTA sensor chip, and KU-57788 was tested at working concentrations of 0 μM, 0.78 μM, 1.56 μM, 3.12 μM, 6.25 μM, 12.5 μM, 25 μM, and 50 μM. Data were analyzed using BIAcore T200 evaluation software, yielding a dissociation constant (K_D_) of 7.858 μM for the interaction between DRP1 and KU-57788 (Fig. 5D). The cellular thermal shift assay (CETSA) was further used to assess the efficiency of KU-57788 binding to DRP1 within cells. CETSA is based on the principle that target proteins often become stabilized upon binding with small molecules, leading to a slower degradation rate under increasing temperatures [29]. When DRP1 was treated with KU-57788, the results showed that under the same temperature conditions, DRP1 protein degradation was significantly slower compared to untreated conditions (Fig. 5E and Fig. S3K). Additionally, we assessed the effect of KU-57788 binding to DRP1 on its activity using an ATPase/GTPase activity assay and found that KU-57788 significantly enhanced DRP1 activity (Fig. 5F). These results collectively confirmed that KU-57788 directly binds to DRP1, thereby modulating its phosphorylation state and promoting its activation.

### KU-57788-mediated activation of DRP1 exhibits a protective effect on ATC cells

**Fig. 6:**
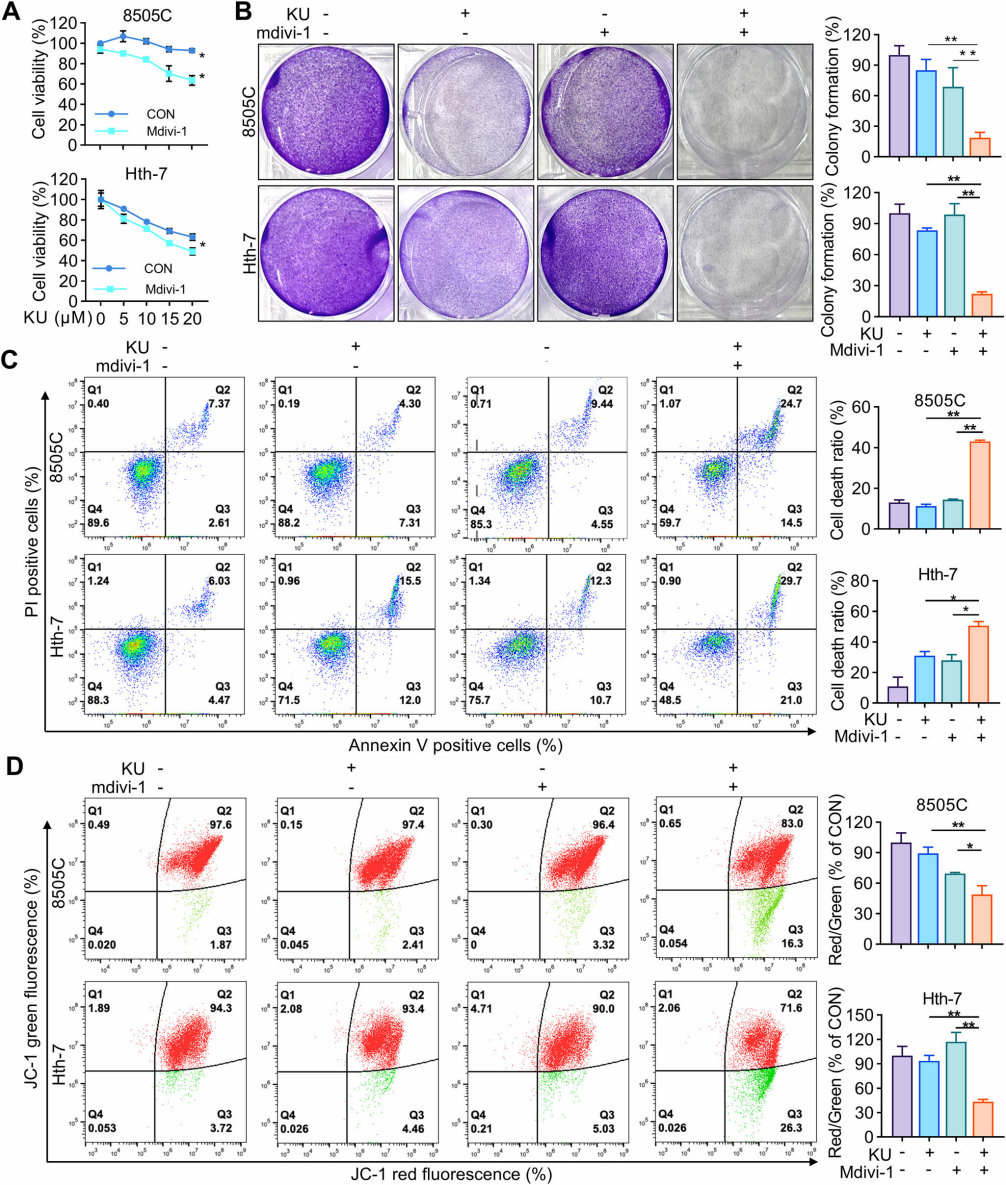
KU-57788-mediated activation of DRP1 exerts a protective effect on ATC cells. **A** In 8505C and Hth-7 cells, cell viabilities following treatment with KU-57788 (0, 5, 10, 15 and 20 μM) for 24 h in the presence or absence of Mdivi-1 (0 and 10 μM) determined by the CCK-8 assay kit, respectively. **B**,**C** and (**D**) Quantitative and qualitative analyze of 8505C and Hth-7 cell clone formation (seeded at 8 × 10⁴ cells/well), cell death and mitochondrial membrane potential at the indicated concentrations of KU-57788 (0 and 10 μM) with or without Mdivi-1 (0 and 10 μM). Data are shown as mean ± SD for *n* = 3, analyzed by one-way ANOVA using the Holm-Sidak method. *p < 0.05, **p < 0.01, ns, *p* > 0.05.

The interaction between KU-57788 and DRP1 promotes DRP1 activation-mediated mitochondrial hyper-fragmentation, which does not lead to substantial ATC cell death. This raises the question of whether DRP1 activation is related to KU-57788’s ability to inhibit ATC cell viability. Therefore, by introducing the DRP1-specific inhibitor mdivi-1, we observed that DRP1 inhibition significantly synergized with KU-57788 to suppress ATC cell viability and colony formation (Fig. 6A and Fig. 6B). Furthermore, DRP1 inhibition cooperatively enhanced KU-57788-induced ATC cell death, shifting the cells from a senescent state to a state of cell death (Fig. 6C). These findings suggest that KU-57788 activates DRP1, promoting mitochondrial fragmentation while also protecting ATC cells from KU-57788-induced cytotoxicity. DRP1 activation appears to provide a protective effect for ATC cells under KU-57788 treatment. Interestingly, DRP1 inhibition markedly enhanced the ability of KU-57788 to reduce mitochondrial membrane potential, indicating severe mitochondrial damage (Fig. 6D). This mitochondrial damage may be a key mechanism underlying the enhanced cytotoxicity observed when combining DRP1 inhibition with KU-57788.

### KU-57788-mediated activation of DRP1 synergistically enhances the sensitivity of ATC cells to ferroptosis

**Fig. 7:**
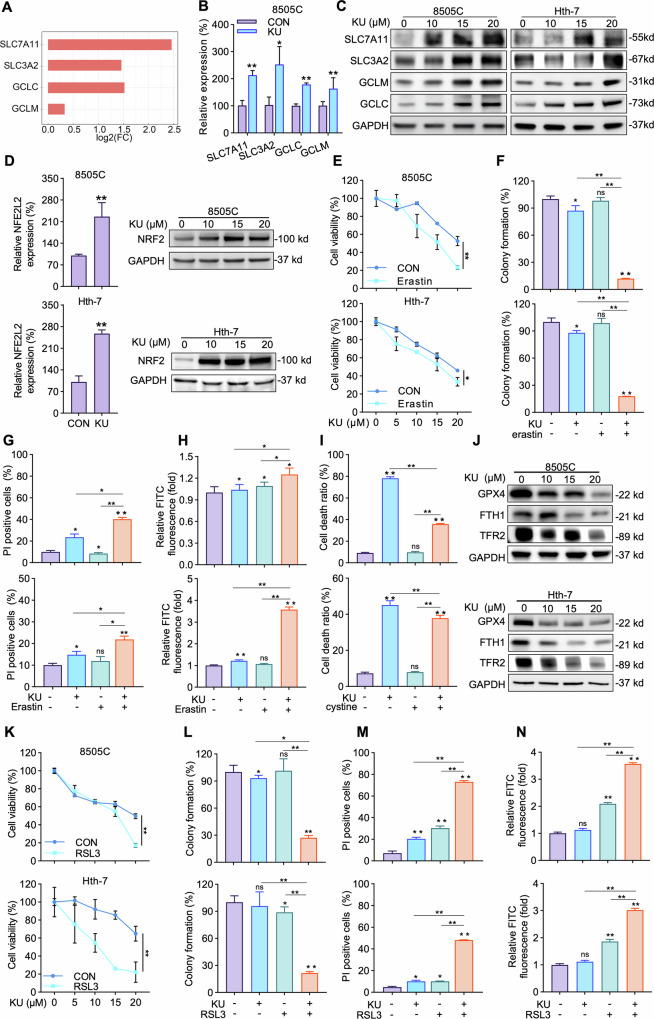
KU-57788-induced mitochondrial fission activates NRF2/SLC7A11/GSH axis and synergistically promotes ferroptosis in ATC cells. **A** The expression levels of genes related to glutathione metabolism and transport. **B**,**C** mRNA and protein change of SLC7A11, SLC3A2, GCLC and GCLM were measured after treating with KU-57788 (0 and 20 μM) for 24 h by qPCR and western blot. **D** RNA and protein change of NFE2L2 were measured after after treating with KU-57788 (0, and 20 μM) for 24 h by qPCR and western blot. **E**,**F**,**G** and (**H**) Quantitative analyze of 8505C and Hth-7 cell cell viabilities, clone formation (seeded at 8 × 10⁴ cells/well), cell death and lipid ROS at the indicated concentrations of KU-57788 (0 and 10 μM) with or without Erastin (0 and 5 μM). **I** Quantitative analyze of 8505 C and Hth-7 cell death at the indicated concentrations of KU-57788 (0 and 10 μM) with or without cystine (200 μM). The groups are as follows: Control (no KU-57788, no cystine); Cystine Deprivation (with KU-57788, no cystine); KU Treatment (no KU-57788, with cystine); KU-57788 + Cystine (with KU-57788, with cystine). **J** Western blot was used to measure protein levels of GPX4, FTH1 and TFR2 related to ferroptosis in 8505C and Hth-7cells after KU-57788 (0, 10, 15, and 20 μM) treatment for 24 h. **K**,**L**,**M** and (**N**) Quantitative analyze of 8505C and Hth-7 cell cell viabilities, clone formation, cell death and lipid ROS at the indicated concentrations of KU-57788 (0 and 10 μM) with or without RSL3 (0 and 2.5 μM). Data are shown as mean ± SD for *n* = 3, analyzed by one-way ANOVA using the Holm-Sidak method. *p < 0.05, **p < 0.01, ns, *p* > 0.05.

Current studies suggest that DRP1-regulated mitochondrial fission dynamics are intricately linked to oxidative stress, with hyperactivation of DRP1 often resulting in excessive mitochondrial fission and fragmentation, elevated ROS levels, and potential cell death [30]. However, our findings indicated that while KU-57788 activated DRP1 and promotes mitochondrial fission, it only slightly elevated elevate ROS levels and lipid ROS levels (Fig. S13A, B). To explore signaling pathway alterations following KU-57788 treatment in 8505C cells, we performed RNA-seq analysis. RNA-Seq data has been submitted to the GEO repository under accession number GSE319513. Strikingly, ferroptosis resistance-related pathways were identified to be significantly upregulated in KU-57788-treated 8505 C cells (Fig. S14A, B), including SLC3A2, SLC7A11, GCLC and GCLM (Fig. 7A). qPCR validation confirmed consistent upregulation of SLC3A2, SLC7A11, GCLC and GCLM in KU-57788-treated 8505C cells (Fig. 7B), while GCLC and GCLM were similarly upregulated in Hth-7 cells (Fig. S14C). Nonetheless, western blot analysis revealed a dose-dependent increase in their protein levels in both 8505C and Hth-7 cells upon KU-57788 treatment (Fig. 7C and Fig. S3L).

As a key transcription factor for antioxidant stress, NRF2 regulates ferroptosis-related target genes by promoting the expression of those involved in redox homeostasis, such as the glutathione (GSH) synthesis pathway genes (e.g., SLC7A11, SLC3A2, GCLM, and GCLC), which play a critical role in neutralizing ROS and lipid peroxides [31]. Consistent with this, we demonstrated that KU-57788 dose-dependently upregulates NRF2 mRNA and protein levels (Fig. 7D and Fig. S3M), suggesting NRF2-mediated activation of the GSH synthesis pathway.

To further validate the relationship between DRP1 and the NRF2/SLC7A11/GSH axis, we constructed DRP1 knockdown and DRP1 overexpression ATC cells, followed by treatment with KU-57788 (Fig. S15). The expression levels of SLC7A11, SLC3A2, GCLC, GCLM, and NRF2 were evaluated by qPCR analyses (Fig. S15). The results showed that knockdown of DRP1 had no significant effect on the expression of the above-mentioned genes in ATC cell lines 8505 C and Hth-7, except for a slight upregulation of GCLC and downregulation of GCLM in 8505C cells, and a mild upregulation of GCLM in Hth-7 cells (Fig. S15A). These findings suggest that DRP1 knockdown does not activate the NRF2/SLC7A11/GSH pathway. In contrast, overexpression of DRP1 in 8505 C cells led to a mild but significant upregulation of most related genes, except SLC7A11, which remained unchanged (Fig. S15B, C and Fig. S3O). Upon further treatment with KU-57788, the expression levels of all genes were markedly increased (Fig. S15B, C and Fig. S3O). These observations indicate that DRP1 overexpression alone may only partially activate the NRF2/SLC7A11/GSH pathway, whereas KU-57788 treatment can markedly enhance its activity. This further suggests that significant activation of the NRF2/SLC7A11/GSH pathway may occur only when DRP1 is in its active form or directly activated.

Furthermore, we introduced erastin, an SLC7A11 inhibitor and ferroptosis inducer. KU-57788 significantly acted synergistically with erastin in both 8505C and Hth-7 cells, leading to suppressed cell viability, colony formation, and induced cell death (Fig. 7E–G and Fig. S16A, B), with notable increases in lipid ROS levels (Fig. 7H and Fig. S16C). Additionally, KU-57788-treated 8505C and Hth-7 cells exhibited a synergistic response to cysteine deprivation in the culture medium, further confirmed the protective role of SLC7A11/GSH axis under KU-57788 treatment. Specifically, depriving these cells of cysteine disrupted glutathione synthesis, consequently exacerbating cell death; conversely, cysteine replenishment restored glutathione synthesis, promoted the clearance of accumulated peroxides, and ultimately alleviated the lethal effects induced by KU-57788 (Fig. 7I and Fig. S16D). Collectively, these results demonstrated that the activation of the NRF2/SLC7A11/GSH axis serves as a protective mechanism, shielding ATC cells from KU-57788-induced cytotoxicity.

In addition, we unexpectedly observed that KU-57788 dose-dependently downregulated the protein expression of GPX4, FTH1 and TFR2 (Fig. 7J and Fig. S3N), despite not inducing ferroptosis and only slightly increasing ROS and lipid ROS levels in ATC cells (Fig. S13A, B). This results further emphasized the protective role of NRF2/SLC7A11/GSH axis activation. Coincidentally, KU-57788-treated 8505C and Hth-7 cells exhibited a marked synergistic effect with GPX4 inhibition (RSL3, a GPX4 inhibitor), with cell viability and colony formation inhibition, increased cell death (Fig. 7K–M and Fig. S16E, F), upregulated lipid ROS (Fig. 7N and Fig. S16G). In addition, we performed cell death reversal experiments using specific inhibitors targeting ferroptosis (Fer-1, Ferrostatin-1), oxidative stress (NAC, N-acetylcystein), necrosis (Nec-1, Necrostatin-1), apoptosis (ZVAD, Z-Val-Ala-Asp(O-Me)-fluoromethylketone), and autophagy (CQ, Baf-A1 and 3-MA) to identify the type of cell death induced by the combined treatment with KU-57788 and RSL3. The results demonstrated that Fer-1 induced the most significant reversal of cell death, while NAC and Nec-1 only provided modest protective effects in 8505C; only Fer-1 and NAC provided protective effects in Hth-7 (Fig S17). These findings suggest a feedback loop were in KU-57788-induced DRP1 activation enhances mitochondrial fission and fragmentation, accompanied by the activation of the NRF2/SLC7A11/GSH axis to neutralize ROS and protect ATC cells from KU-57788 cytotoxicity. Simultaneously, this pathway synergistically enhances ferroptosis under specific conditions, establishing KU-57788 as a novel synergist in ferroptosis.

### KU-57788 suppresses ATC tumor growth and acts synergistically with ferroptosis inducers in vivo

**Fig. 8:**
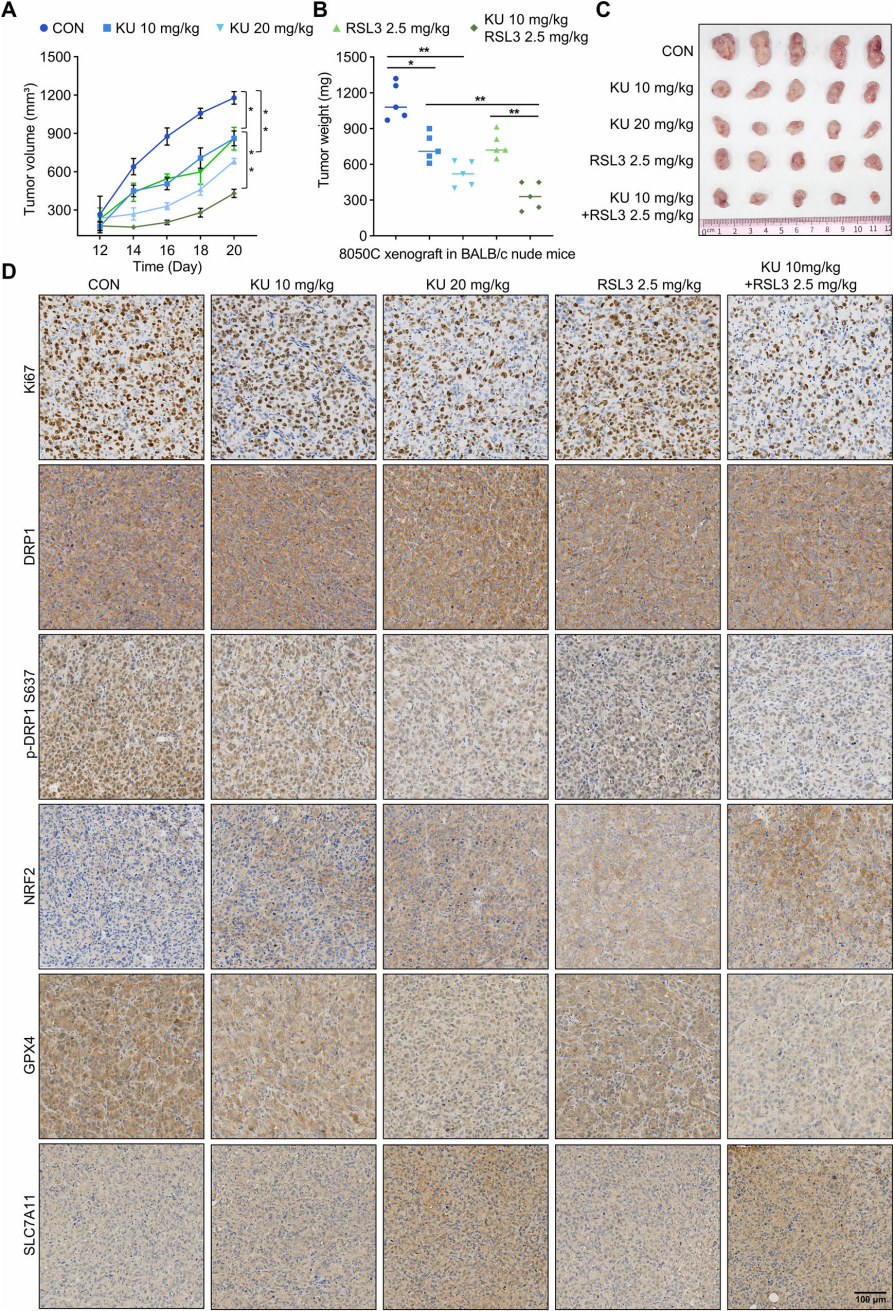
KU-57788 in combination with RSL3 inhibited the growth of mice xenografts in vivo. **A** Tumor volumes were measured every other day, growth curves of tumors treated with KU-57788 (0, 10 and 20 mg/kg, intraperitoneal injection) and RSL3 (0 and 2.5 mg/kg, intraperitoneal injection) (*n* = 5/group) were analyzed by GraphPad Prism 9 software. Data are shown as mean ± SD. **B** Bar charts showed average tumor weights and body weights of each group. Data are shown as mean ± SD (*n* = 5). **C** Representative gross image of tumors at the endpoint of the experiment. **D** Ki67, DRP1, p-DRP1 S637, NRF2, GPX4 and SCL7A11 expression levels in tumors as measured by IHC staining assay. Data are shown as mean ± SD for *n* = 3, analyzed by one-way ANOVA using the Holm-Sidak method. *p < 0.05, **p < 0.01, ns, *p* > 0.05.

We have revealed that KU-57788, by activating DRP1, promotes excessive mitochondrial fission and fragmentation in ATC cells, and activates the NRF2/SLC7A11/GSH axis to protect against cell death, while also suggesting that KU-57788 can act as a synergist in ferroptosis. To further investigate this, we established a subcutaneous xenograft tumor model in BALB/c nude mice using 8505C cells to assess the in vivo inhibitory effect of KU-57788 on ATC tumorigenesis. Concurrently, we employed RSL3, a commonly used ferroptosis inducer, in combination with KU-57788 to evaluate its role as a ferroptosis synergist. After subcutaneous tumor formation, mice were randomly divided into several groups and treated with different drugs via intraperitoneal injection, including the control group (DMSO), KU-57788 (10 mg/kg), KU-57788 (20 mg/kg), RSL3 (2.5 mg/kg), and a combination group (KU-57788 10 mg/kg + RSL3 2.5 mg/kg). The results showed that, compared to the control group, KU-57788 significantly inhibited tumor growth in a dose-dependent manner, with the combination of KU-57788 and RSL3 achieving over 80% tumor growth inhibition, demonstrating a significant synergistic effect (Fig. 8A–C). No significant changes in body weight were observed across any treatment groups (Fig. S18A). Histological analysis of heart, liver, and kidney tissues using H&E staining revealed no detectable pathological alterations (Fig. S18B). Additionally, biochemical indicators of liver and kidney function remained unaffected (Fig. S18C), further demonstrating the safety profile of the treatments. Additionally, IHC staining revealed a dose-dependent downregulation of Ki-67, p-DRP1 S637 and GPX4 levels, with a significant upregulation of NRF2 and SLC7A11 protein levels (Fig. 8D). These results are consistent with in vitro studies, indicating that KU-57788 not only inhibits the growth of ATC subcutaneous xenografts but also synergizes with ferroptosis inducers to promote ferroptosis.

## Discussion

ATC is an aggressive and highly lethal thyroid malignancy, distinguished by its rapid progression, poor prognosis, and status as the deadliest subtype of thyroid cancer, despite its low incidence [32]. ATC is notably resistant to conventional treatment modalities, including surgery, radiotherapy, and chemotherapy, and lacks effective targeted therapies and immunotherapy [33, 34]. As a result, therapeutic options for ATC remain severely limited, with a median survival time of only a few months for most patients. This highlights an urgent need for the development of innovative therapeutic strategies to improve survival and improve patient outcomes. In this study, we preliminarily revealed the role of DNA-PKcs in promoting the malignant phenotype of ATC and demonstrated the anti-ATC activity of its inhibitor, KU-57788. Additionally, we identified a strategy in which KU-57788 acts as a synergist to enhance the efficacy of ferroptosis inducers, offering potential combinatorial therapeutic strategies and novel research directions for ATC treatment.

Targeted therapy has emerged as a crucial strategy in cancer treatment and has garnered significant attention due to its high specificity [35]. By selectively targeting tumor-associated targets, it effectively suppresses tumor cell activity while sparing normal cells from excessive damage [36, 37]. However, in clinical applications, targeted therapies often exhibit multi-target effects, which can lead to both toxic side effects on normal cells and enhanced tumor cell cytotoxicity [37]. Our study demonstrates that the DNA-PKcs inhibitor KU-57788 exhibits significant anti-ATC activity both in vitro and in vivo. More importantly, we innovatively discovered that independent of its primary target, DNA-PKcs, KU-57788 binds to and activates DRP1, leading to excessive mitochondrial fission and activation of the NRF2/SLC7A11/GSH axis, thereby protecting ATC cells from ferroptosis. Unlike conventional compensatory pathway activation, KU-57788 exerts its effect on a novel target, DRP1, inducing mitochondrial hyper-fragmentation-mediated oxidative stress protection, which ultimately attenuates its own anticancer activity. These findings suggest that the multi-target nature of targeted therapies may paradoxically confer protective effects on tumor cells, allowing them to counteract the cytotoxic effects of targeted drugs by activating or inhibiting potential molecular targets. Our study provides novel insights into the target diversity of anticancer agents and offers new research directions for enhancing the efficacy and specificity of targeted cancer therapies.

Mitochondrial dynamics, including fission and fusion, are integral to maintaining the malignant phenotype of tumors by regulating mitochondrial function, energy metabolism, tumor cell survival, resistance to apoptosis, invasion, metastasis, and responsiveness to chemotherapy and radiotherapy [38, 39]. In tumor cells, mitochondria predominantly favor the fission state, as this process generates a higher number of mitochondria to meet the increased metabolic demands and rapid proliferation typical of cancer cells [39, 40]. Moreover, excessive mitochondrial fission leads to membrane depolarization, which reduces the activation of death signals, thereby promoting tumor cell survival [41]. As a result, targeting mitochondrial fission has emerged as a promising strategy in cancer treatment. Mitochondrial fission and fusion are governed by a balance between pro-fission proteins (such as DRP1, Fis1, and MFF) and pro-fusion proteins (such as MFN1, MFN2, OPA1, MIEF1, and MIEF2), with DRP1 standing out as the key regulator of mitochondrial fission [42]. DRP1 mediates the constriction of the mitochondrial outer membrane through its GTPase activity, initiating fission [43]. Notably, DRP1 is often upregulated in various cancers, driving malignant progression, chemoradiotherapy resistance, and poor prognosis [44–46]. Inhibition of DRP1 expressions or activity has been shown to significantly suppress tumor cell proliferation, invasion, and migration, while enhancing apoptosis and increasing synergistic responses to chemotherapy and radiotherapy [47, 48]. Our previous studies have demonstrated that DRP1 is specifically upregulated in ATC tissues, and its transcriptional inhibition impairs mitochondrial fission, induces mitochondrial membrane potential loss, and triggers cytochrome c release, leading to pyroptosis and apoptosis in ATC cells [25, 49, 50]. These findings underscore the pivotal role of DRP1 in sustaining the malignant phenotype of ATC. In this study, we explore a novel dimension of DRP1 activity, focusing on its hyperactivation as a potential therapeutic strategy for ATC. We identified KU-57788, a novel DRP1 agonist, which further enhances DRP1 activity, inducing excessive mitochondrial fission and fragmentation, mitochondrial membrane potential loss, and accelerating mitophagy in ATC cells. Notably, ATC cells respond by activating the NRF2/SLC7A11/GSH axis to protect themselves, and blocking this pathway synergistically enhances KU-57788-induced ferroptosis. Our findings reveal that hyperactivation of DRP1 can be leveraged as a strategy to synergistically promote ferroptosis in ATC cells, offering a promising combination approach for cancer therapy.

Inducing ferroptosis in tumor cells has emerged as a promising anticancer strategy, with mitochondrial dynamics playing a crucial regulatory role—an area of growing interest in recent ferroptosis research [51, 52]. Ferroptosis is an iron-dependent form of programmed cell death characterized by intracellular iron accumulation and elevated lipid peroxidation, ultimately leading to membrane rupture and cell death [53]. As the central hub of cellular energy production and metabolism, mitochondria constantly undergo fission and fusion, processes that are closely linked to ferroptosis regulation [54]. Excessive mitochondrial fission in tumor cells triggers oxidative stress, leading to a sharp increase in reactive oxygen species (ROS), which in turn promotes lipid peroxidation and facilitates ferroptosis [55]. Moreover, excessive fission disrupts mitochondrial membrane potential, causes mitochondrial damage, and impairs both cellular energy metabolism and iron homeostasis, further sensitizing cells to ferroptosis [56]. In contrast, mitochondrial fusion supports cellular resilience by maintaining mitochondrial function, integrating metabolic activities, preserving mitochondrial integrity, and effectively scavenging ROS, thereby reducing lipid peroxidation and mitigating ferroptosis [56]. DRP1, a key regulator of mitochondrial fission, is closely associated with excessive mitochondrial fission when hyperactivated. Suppressing DRP1 expression prevents excessive fission, preserves mitochondrial integrity, maintains redox homeostasis, and protects tumor cells from ferroptosis [57]. Our study demonstrates that DRP1 hyperactivation induces excessive mitochondrial fission, thereby synergistically enhancing the susceptibility of ATC cells to ferroptosis. Furthermore, we found that DRP1-driven mitochondrial fission is accompanied by disruptions in iron metabolism, creating a favorable intracellular environment for ferroptosis in ATC cells. Interestingly, despite DRP1-induced mitochondrial fission, we did not observe a significant increase in ROS levels, likely due to the activation of the NRF2/SLC7A11/GSH axis, which facilitates ROS clearance. Notably, blocking this pathway significantly enhanced the ferroptosis-inducing effects of DRP1 hyperactivation in ATC cells. Collectively, our findings provide new insights into the mechanistic link between mitochondrial dynamics and ferroptosis regulation in tumor cells, expanding the current understanding of this emerging field. Although these results highlight a role for KU-57788-activated DRP1 in modulating the NRF2/SLC7A11/GSH axis, we cannot exclude the possibility that KU-57788 exerts its anti-tumor effects through additional parallel mechanisms. Future studies will be essential to delineate the relative contribution of this pathway to the overall efficacy of KU-57788 and to explore its potential as a biomarker for treatment response.

In summary, we demonstrated the potent antitumor activity of the DNA-PKcs inhibitor KU-57788 against ATC and uncovered a novel, DNA-PKcs-independent mechanism underlying its effects. Specifically, we identified KU-57788 as a DRP1 agonist that induces excessive mitochondrial fission, which in turn activates the NRF2/SLC7A11/GSH axis as a protective response in ATC cells to mitigate its cytotoxic effects. Notably, inhibiting this axis or combining KU-57788 with ferroptosis-inducing agents significantly enhances its therapeutic efficacy. Our findings not only establish KU-57788 as a DRP1 agonist but also highlight its potential as a synergistic inducer of ferroptosis, providing a rationale for a combinatorial approach to ATC treatment. These findings offer valuable insights into improving targeted cancer therapies and serve as a theoretical foundation for enhancing their antitumor effects.

## Materials and methods

### Drugs and reagents

KU-57788 (#T6276), Mdivi-1 (#T1907), RSL3 (Cat: T3646), Ferrostatin-1 (#T13360), 3-methyladenine (#T1879), CQ (#T0194), BafA1 (#T6704), Necrostatin-1 (#T1847), Tween 80 (#T13947) and PEG300 (#T7022) were procured from TargetMol (USA). The DAPI (4′,6-Diamidine-2′-phenylindole dihydrochloride, #C1002), N-acetyl-L-cysteine (#S0077) and the tubulin-tracker Green (#C1051S) was purchased from Beyotime Biotechnology (China). The MitoTracker® Deep Red FM (#8778) was purchased from Cell Signaling Technology (MA, USA). The Imidazole Ketone Erastin (#GC52190) and Z-VAD-FMK (#GC12861) was purchased from GlpBio (Montclair, USA). ATPase/GTPase Activity Assay Kit (#MAK113) was purchased from Merck (Darmstadt, Germany).

### Cell culture

Human ATC cell lines Hth-7, KHM-5M, KMH-2, C643, and CAL-62 were purchased from the National Infrastructure of Cell Line Resource (Shanghai, China), while the 8505C cell line was obtained from Deutsche Sammlung von Mikroorganismen und Zellkulturen (DSMZ). Hth-7 were cultured in DMEM (Hyclone) supplemented 10% fetal bovine serum (#S-FBS-SA-015, SERANA, Germany), other cells were cultured in RPMI1640 (Hyclone) supplemented 10% fetal bovine serum (#S-FBS-SA-015, SERANA, Germany). The cells were maintained at 37 °C with 5% CO_2_.

### Cell viability and cell proliferation curve experiment

Cell viability was assessed using a CCK-8 assay (Cat: A311-01; Vazyme Biotech Co., Ltd., China) following the manufacturer’s protocol. Cells were seeded into 96-well plates at 5000 cells per well and incubated at 37 °C with 5% CO_2_ for 24 h before treatment. Dimethyl sulfoxide (DMSO) was added at ≤1 μL/mL, along with varying concentrations of drugs. Afterward, the CCK-8 reagent was added, and the cells were incubated for 1-4 hours at 37 °C and 5% CO_2_. As for cell proliferation curve experiment, RNA interference was used to knock down the target gene, and cells were seeded into a 96-well plate at a density of 2000 cells per well. Cell proliferation was measured at various points (0 h, 24 h, 48 h, 72 h) using the CCK-8 assay, and a growth curve was subsequently constructed. Absorbance at 450 nm was measured using the Synergy LX Multi-Mode Reader (BioTek Instruments, USA).

### Cloning formation

8505C and Hth-7 cells were seeded at an initial density of 2 × 10⁴–8 × 10⁴ cells per well, depending on growth rate, and treated with different concentrations of drugs for one week. After incubation, the plates were washed with PBS, fixed in paraformaldehyde for 15 min and stained with 0.1% crystal violet for 20 min, stained with crystal violet, and photographed. The results were quantitatively analyzed using ImageJ software (National Institutes of Health, USA).

### siRNA transfection

Beijing Tsingke Biotech Co., Ltd., China, synthesized the siRNA that targets DNA-PKcs, DRP1 and the negative control siRNA. The siRNA sequence of DNA-PKcs was: 5-CTTTATGGTGGCCATGGAGTT-3′, DRP1 was 5'-GCTACTTTACTCCAACTTATT-3'. According to the instructions provided by the manufacturer, Lipofectamine 3000 (Cat.NO. L3000015, Invitrogen) was used to transfect the siRNAs into ATC cells. After 48 h of transfection, the cells were examined using real-time quantitative PCR (qPCR) and western blotting to confirm the effectiveness of siRNA knockdown and used for experiments.

### SA-β-gal staining

SA-β-gal staining was performed using the Senescence β-Galactosidase (SA-β-gal) Staining kit (Beyotime, Cat #C0602, China). 8505C and Hth-7 were washed with PBS and incubated in a fixative solution for 15 min at room temperature. Then, the cells were washed with PBS and incubated in SA-β-gal staining solution at 37 °C overnight without CO_2_. Images were captured using a phase-contrast optical microscope (Thermo Fisher, Evos M7000, MA, USA).

### Determination of cell death rate

Cell death was assessed using an Annexin V-FITC and PI Kit (MultiSciences, China) following the manufacturer’s instructions. Cells were seeded at a density of 1.5 × 10⁵ per well and treated with different drug concentrations for 24 h. After treatment, the cells were harvested, washed with PBS, and stained with PI and Annexin V-FITC. Fluorescence data were collected using the NovoCyte® Quanteon® Benchtop Flow Cytometer and analyzed with FlowJo V10.

### JC-1 functional assay

The mitochondrial membrane potential was determined using JC-1 (Cat 40705ES03, Yeasen Biotechnology, China) according to the instructions provided by the manufacturer. Following a 12 h treatment, the 8505C and Hth-7 cells were exposed to JC-1 at a concentration of 5 µg/mL for a duration of 30 min. Subsequently, they were rinsed twice and reconstituted in PBS. Stained cells were analyzed using a NovoCyte® Quanteon® Benchtop Flow Cytometer and analyzed with FlowJo V10.

### Morphological examination and fluorescence staining

After treating the cells with various substances for the specified durations, they were observed using a phase-contrast optical microscope (Thermo Fisher, Evos M7000, MA, USA). To examine the mitochondria, ATC cells were treated with KU-57788 for a set period, stained with MitoTracker® Deep Red FM in the dark for 30 min, and then imaged using a Leica STED Confocal laser microscope (Germany).

For immunofluorescence staining, after treatment, cells were rinsed with PBS and fixed with 4% paraformaldehyde. Blocking was performed using a solution of 3% BSA, 0.4% gelatin, and Tris-Buffered Saline with Tween 20 (TBST, containing Tris-Hcl, NaCl and 0.1% tween20). For the double antibody staining of LC3B, DRP1, and HSP60, cells were incubated with primary antibodies overnight at 4 °C, followed by washing with PBS. Alexa Fluor 488-labeled Goat Anti-Rabbit IgG (#A0423, Beyotime Biotechnology, China) and Alexa Fluor 594-labeled Donkey Anti-Mouse IgG (#A0460, Beyotime Biotechnology, China) were used as secondary antibodies. Finally, the cells were stained with DAPI (4’, 6-Diamidino-2-phenylindole, #C1002, Beyotime Biotechnology, China) for 10 minutes. After PBS washing, images were captured using a Leica TCS SP8 Confocal laser microscope (Germany). After acquiring fluorescence images using confocal microscopy, signals from distinct fluorescence channels were extracted with ImageJ software, and Pearson’s correlation coefficient values were calculated.

### Transmission electron microscopy

8505C and Hth-7 cells were treated with KU-57788 for 24 h, then harvested and fixed with an electron microscope fixative (HaoKe Biotechnology Co. Ltd, China). After rinsing with 0.1 M phosphate buffer (pH 7.4), the cells were incubated in 1% OsO_4_ in 0.1 M PB for 2 h, followed by dehydration with a gradient of alcohol and 100% acetone. The samples were then embedded, polymerized, sectioned ultrathin, stained, and imaged using a transmission electron microscope (HT7650, HITACHI, Tokyo, Japan).

### Intracellular reactive oxygen species (ROS) and lipid ROS determination

Following a 24 h treatment, the cells were subjected to staining using 10 µM DCFH-DA (#T15458, TargetMol, USA) or 20 µM C11 Bodiphy 581/591 (#D3861, Thermo Fisher Scientific, USA) in a dark environment for 30 min. Subsequently, the cells were rinsed twice with PBS. The cells were examined with an Agilent NovoCyte Advanteon (California, USA) to ascertain the intracellular levels of ROS and lipid ROS, respectively.

### Western blotting

The treated cells were lysed using Cell lysis buffer for Western and IP (#P0013, Beyotime Biotechnology, China) with PMSF (100 µM, Beyotime Biotechnology, China). Protein concentration was measured by BCA assay (Thermo Fisher Scientific, USA). Proteins were separated by SDS-PAGE, transferred to polyvinylidene fluoride (PVDF) membranes, and blocked with 5% skim milk in TBST for 1 h. Membranes were incubated with primary antibodies overnight at 4 °C, followed by HRP-conjugated GAPDH Rabbit mAb (#AC054, 1:5000, ABclonal) for 1 h at 25 °C. Chemiluminescence was detected using a ChemiDoc-MP imager (Bio-Rad) and FD Bio-Dura ECL kit (Fdbio Science, China). The relative intensity of Western blot bands was quantified by density analysis using ImageJ software and normalized against an internal control protein to assess the expression level of the target protein.

The following primary antibodies diluted in TBST (containing 4% gelatin and 3% BSA) were used: anti-DRP1 (#8570, 1:3000, Cell Signaling Technology), anti-p-DRP1 S616 (#3455S, 1:3000, Cell Signaling Technology), anti-p-DRP1 S637 (#4867S, 1:3000, Cell Signaling Technology), anti- GAPDH (#60004-1-Ig, 1:5000, Proteintech), anti-Cyclin B1 (#4138, 1:3000, Cell Signaling Technology), anti-p16 (#1002202-23, 1:2000, abcam), anti-p62 (#ab109012, 1:3000, abcam), anti-LC3B (#ab192890, 1:3000, abcam), anti-Pink1 (#23274-1-AP, 1:3000, Proteintech), anti-Beclin1 (11306-1-AP, 1:3000, Proteintech), anti-TFR2 (#A9845, 1:5000, ABclonal), anti-FTH1 (#A19544, 1:5000, ABclonal), anti-HO-1 (#10701-AP, 1:2000, Proteintech), anti-GPX4 (ab125066, 1:5000, Abcam), Anti-HSP60 (66041-1-Ig, 1:3000, Proteintech), anti-SLC7A11 (#26864-1-AP,1:3000, Proteintech), anti-SLC3A2 (#15193-1-AP, 1:3000, Proteintech), anti-NRF2 (#16396-1-AP, 1:3000, Proteintech), anti-BNIP3 (#68091-1-Ig, 1:3000, Proteintech) and anti-Parkin (#2132, 1:3000, Cell Signaling Technology).

### Molecular docking

In this experiment, molecular dynamics simulations were performed using Gromacs software. The protein system was initially embedded in a solvent box containing the TIP3P water model, with a minimum boundary distance of 10 Å from the protein. Appropriate amounts of Na^+^ or Cl^-^ ions were added to maintain charge neutrality. The constructed system underwent energy minimization under the amber99sb force field to eliminate unreasonable atomic contacts and prevent simulation collapse. Following minimization, NVT and NPT equilibration steps were conducted, with the simulation set to run in the NPT ensemble at 300 K and 1.01325 bar. The SHAKE algorithm was employed to constrain bond lengths involving hydrogen atoms, with a time step of 2 fs and a total simulation time of 100 ns. Throughout the molecular dynamics simulation, the system’s energy, protein structure, and small molecule RMSD fluctuations were monitored to study the time-dependent changes in relevant properties.

### Surface plasmon resonance

NTA chips were used in this study and 5 mM NiCl_2_ was flowed over the chip surface at 30 μL/min for 120 s to capture divalent nickel ions. Subsequently, the Recombinant Human Dynamin-1-like protein (DRP1, #CSB-EP007078HU, Cusabio Biotech, Wuhan, China) was diluted to 10 μg/mL in PBS and injected at 10 μL/min for an appropriate duration. To analyse the binding properties of the target protein to the analyte KU-57788, we set a maximum concentration of 50 μM in a 1% DMSO system and established eight dilution concentrations from 0 μM to 50 μM in 2-fold increments. Analyses were performed at a flow rate of 30 μL/min, with a binding time of 120 s and a dissociation time of 180 s. Kinetic parameters were determined using multiple cycles, response signals were plotted versus analysis time and fitted to a 1:1 Langmuir binding model using BIAcore T200 software to obtain binding and dissociation rate constants.

### Quantitative real-time PCR (qPCR)

Total RNA from the treated cells was extracted using RNAisoPlus (Takara, Japan), and cDNA was synthesized using PrimeScript^TM^ RT Master Mix (Takara, Japan). Quantitative real-time PCR was performed using Hieff^®^ qPCR SYBR Green Master Mix (Yeasen Biotech Co., Ltd, China) on LC-480II (Roche, Swiss). The qPCR primer sequences for genes are shown in supplement table 1. which was used as the internal reference gene. The relative mRNA levels were calculated using the 2^–ΔΔCt^ method.

### GTPase activity analysis

To measure the GTPase activity of the DRP1 protein using an ATPase/GTPase Activity Assay kit (#MAK113, Sigma-Aldrich, USA), first, prepare a standard curve for phosphate concentration by mixing phosphate standards and purified water according to the instructions. For the sample preparation, add 10 µg of DRP1 enzyme and KU-57788 sample to wells in a 96-well plate and adjust the volume to 10 µL with the assay buffer. For the reaction, prepare the reaction mix by adding assay buffer and 4 mM GTP to the sample wells. Incubate the plate at room temperature for 30 min to allow the reaction to occur. After incubation, add the malachite green reagent to each well and incubate for another 30 min to terminate the reaction and develop the colorimetric product. Measure the optical density at 620 nm to quantify the GTPase activity, calculate the ΔOD, and plot the standard curve to determine the free phosphate concentration produced during the reaction. Finally, calculate the enzyme activity using the formula provided in the kit instructions, considering the sample volume, reaction time, and phosphate concentration.

### Cellular thermal shift assay (CETSA)-Western blot (WB)

The CETSA-WB experiment was performed as previously described [29]. In brief, soluble protein lysates from 8505C and Hth-7 cells were aliquoted into PCR tubes and treated with KU-57788 (20 µM) or DMSO for 2 h at room temperature, prior to the CETSA heat pulse. The samples were subjected to heat treatment at temperatures ranging from 40 °C, 50 °C, 60 °C, 70 °C, 80 °C and 90 °C for 3 min, followed by a 3-minute cooling period at 4 °C in a thermocycler. After centrifugation at 20,000 × *g* for 20 min at 4 °C, the resulting soluble supernatant was analyzed by western blotting.

### Xenograft tumor model

The animal trials were carried out by the authorized procedure of the Animal Ethics Committee of Zhejiang Provincial People’s Hospital (Approval NO. 20241012162462). Female BALB/c nude mice (3-4 weeks old) were obtained from Shanghai SLAC Animal Co. (China). 8505C cells (5 × 10⁶ cells/100 µL) were subcutaneously implanted into the right axilla. Once tumors became palpable, mice were randomly divided into groups (*n* = 5/group). KU-57788 and RSL3 were diluted in a solution of 5% DMSO, 30% PEG300, 10% Tween 80, and 55% PBS. Mice received 100 µL of the drug mix intraperitoneally daily for 12 days. Mice were weighed regularly, and tumor volumes were measured every other day using the formula: Tumor vol (mm³) = 0.5 × (short diameter) ² × (long diameter). After 12 days, mice were euthanized, and tumors, blood, and organs (liver, kidneys, heart) were collected, fixed in 4% formalin, embedded in paraffin, sectioned, and stained with hematoxylin and eosin.

### Immunohistochemical staining and hematoxylin-eosin (HE) staining

Tissue sections (4 μm thick) were cut from paraffin blocks, deparaffinized, rehydrated, and treated with citrate buffer (pH 6.0) for antigen retrieval. After blocking with 3% BSA for 30 min, the sections were incubated with the primary antibody (Ki67, 1:500; DRP1, 1:100; p-DRP1 s637, 1:200; NRF2, 1:400; GPX4, 1:100; SLC7A11, 1:200) for 1 h at 25 °C, followed by the HRP-labeled secondary antibody (#IKI0029, HaoKe Biotechnology Co. Ltd, China) for 30 min at room temperature. Nuclei were stained with hematoxylin (blue), and positive staining was observed as a brownish-yellow color. Images were captured using a fluorescence microscope (Konfoong Biotech, Ningbo, China).

Paraform sections were deparaffinized by sequential immersion in Xylene (I, II, III, #10023418, Sinopharm Chemical Reagent Co., Ltd, China) and graded alcohols (absolute ethanol I, II, 85%, and 75%) followed by washing in tap water. Hematoxylin staining was performed for 5 min, differentiated with hydrochloric acid for 2 s, blued with ammonia water for 15–30 s, and washed with water. Eosin staining was done for 5–8 s. Sections were dehydrated through alcohol and Xylene, then mounted with neutral balsam. The slides were observed under a microscope, and images were captured and analyzed.

### Statistical analysis

A minimum of three repetitions were conducted for all experiments. Data is presented as mean ± SD. The *t*-test was used to compare two groups, while one-way ANOVA was applied for multiple group comparisons. *P*-values were two-sided, with significance set at *P* < 0.05. Statistical analysis was conducted using GraphPad Prism 9 (GraphPad Software, USA).

## Supplementary information

Original Western Blot

Supplement figure

## Data Availability

The datasets used and/or analysis during the current study are available from the corresponding author on reasonable request.
