# Supplementary material for: Direct coupling and protective activation of DRP1 by the DNA-PKcs inhibitor KU-57788 synergizes with ferroptosis in anaplastic thyroid cancer cells
Source: Cell Death Dis. 2026 Apr 28;17(1):570. doi: 10.1038/s41419-026-08595-3 (PMC13265778; doi:10.1038/s41419-026-08595-3)
Supplement: Supplementary file 1 — Original Western Blot [file 41419_2026_8595_MOESM1_ESM.pdf]

Figue 1

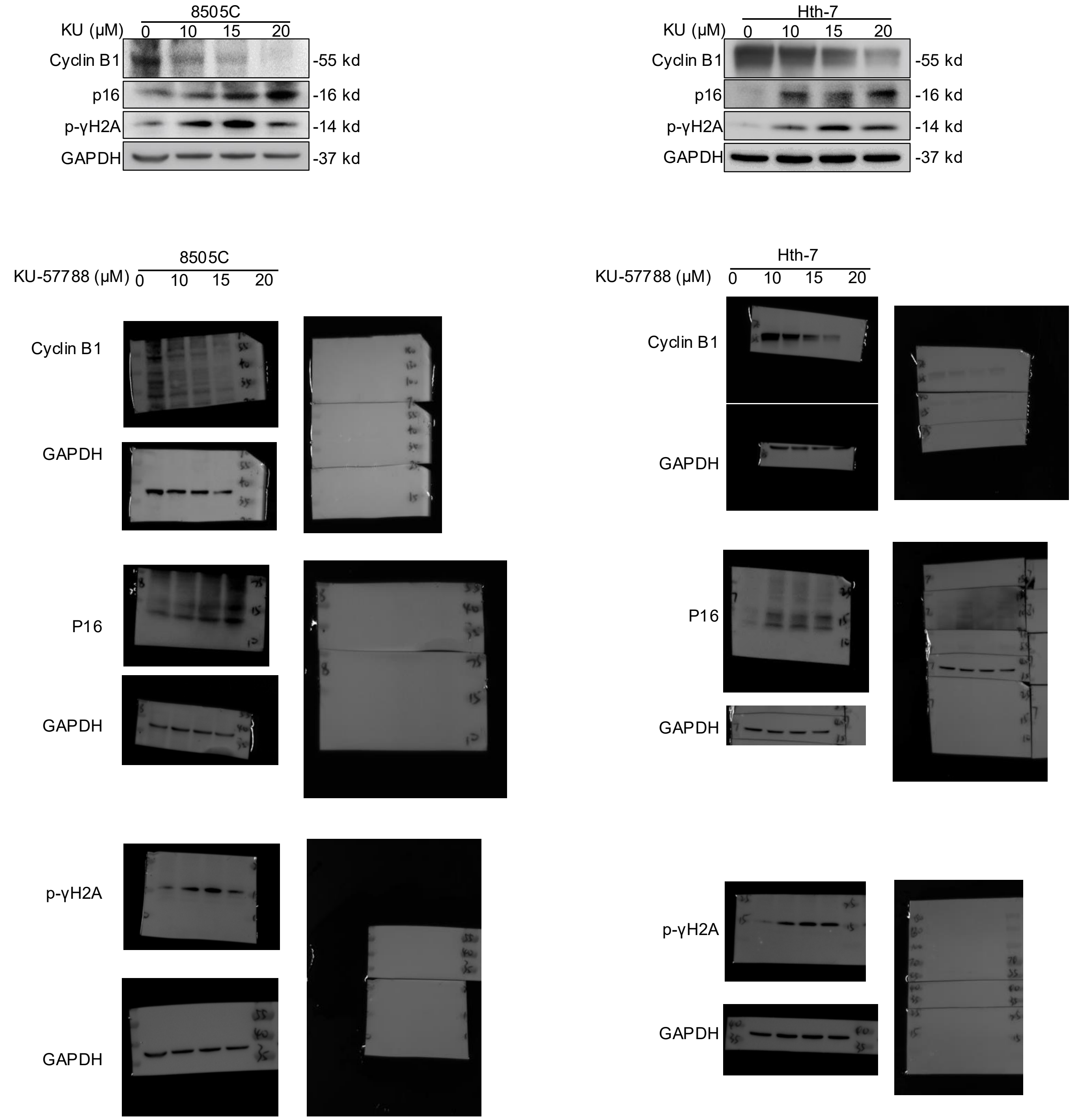
8505C
KU (μM) 0 10 15 20
Cyclin B1
p16
p-γH2A
GAPDH
-55 kd
-16 kd
-14 kd
-37 kd
Hth-7
KU (μM) 0 10 15 20
Cyclin B1
p16
p-γH2A
GAPDH
-55 kd
-16 kd
-14 kd
-37 kd
8505C
KU-57788 (μM) 0 10 15 20
Cyclin B1
GAPDH
P16
GAPDH
p-γH2A
GAPDH
Hth-7
KU-57788 (μM) 0 10 15 20
Cyclin B1
GAPDH
P16
GAPDH
p-γH2A
GAPDH

Figue 2

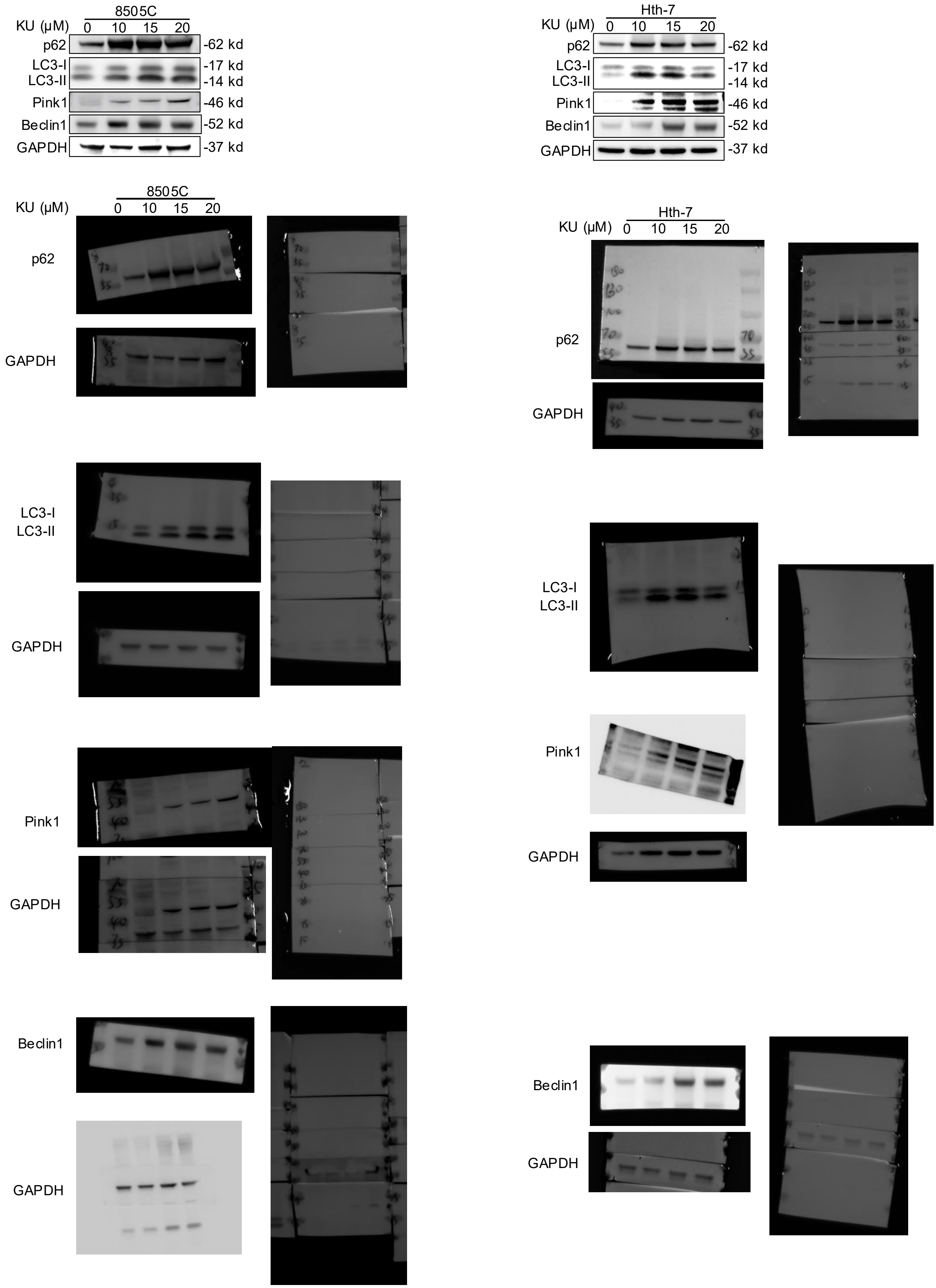

8505C
KU (μM) 0 10 15 20
p62 -62 kd
LC3-I -17 kd
LC3-II -14 kd
Pink1 -46 kd
Beclin1 -52 kd
GAPDH -37 kd
Hth-7
KU (μM) 0 10 15 20
p62 -62 kd
LC3-I -17 kd
LC3-II -14 kd
Pink1 -46 kd
Beclin1 -52 kd
GAPDH -37 kd
8505C
KU (μM) 0 10 15 20
p62
GAPDH
LC3-I
LC3-II
GAPDH
Pink1
GAPDH
Beclin1
GAPDH
Hth-7
KU (μM) 0 10 15 20
p62
GAPDH
LC3-I
LC3-II
Pink1
GAPDH
Beclin1
GAPDH

Figue 4

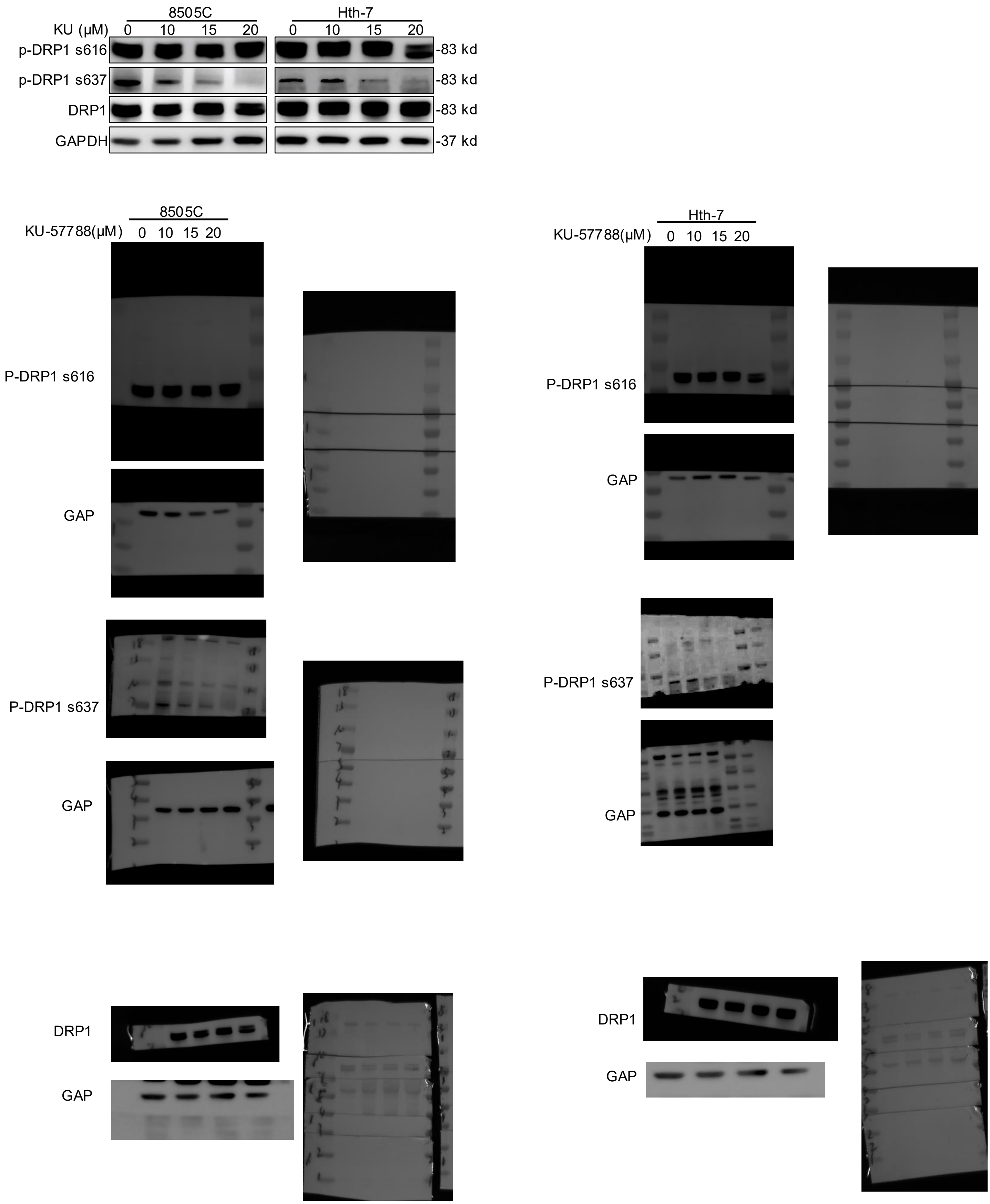
8505C
Hth-7
KU (μM)
0 10 15 20
0 10 15 20
p-DRP1 s616
p-DRP1 s637
DRP1
GAPDH
-83 kd
-83 kd
-83 kd
-37 kd
8505C
KU-57788(μM) 0 10 15 20
Hth-7
KU-57788(μM) 0 10 15 20
P-DRP1 s616
GAP
P-DRP1 s637
GAP
P-DRP1 s616
GAP
P-DRP1 s637
GAP
DRP1
GAP
DRP1
GAP

Figue 5

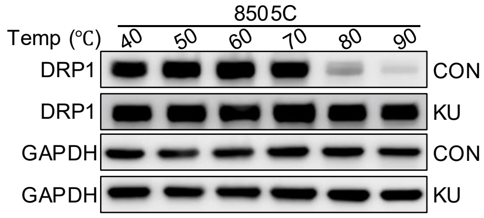
8505C
Temp (℃) 40 50 60 70 80 90
DRP1 CON
DRP1 KU
GAPDH CON
GAPDH KU

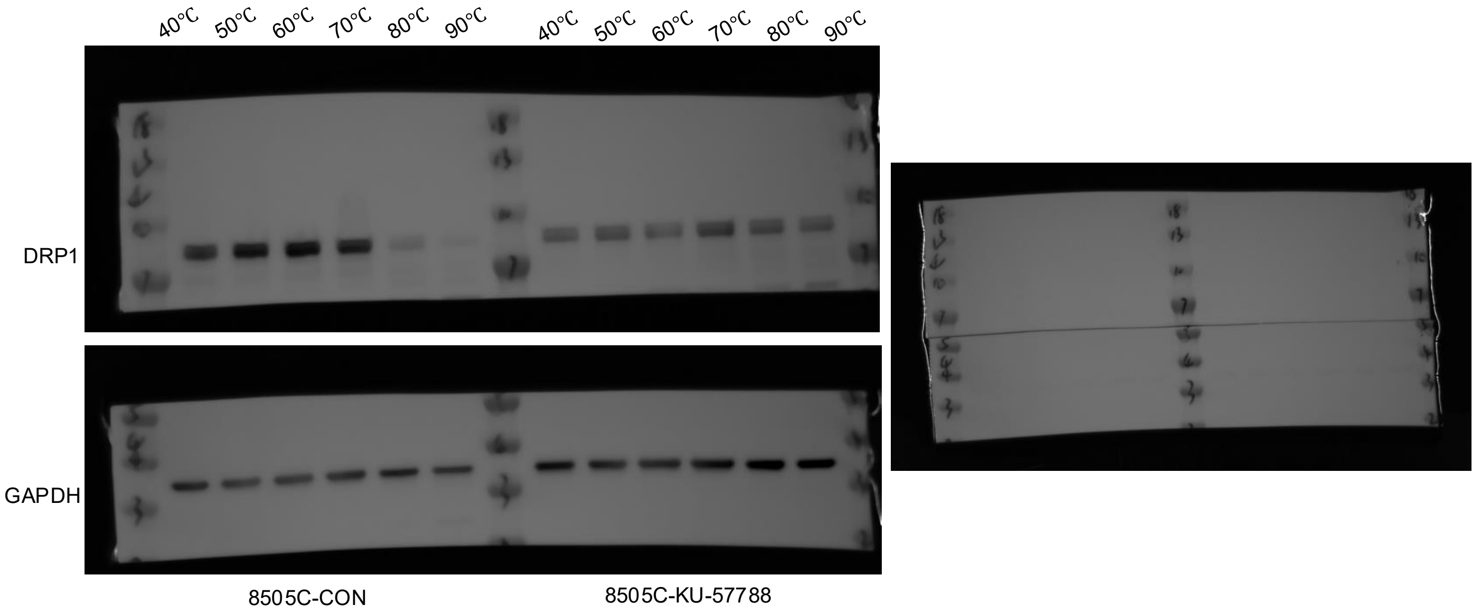
40℃ 50℃ 60℃ 70℃ 80℃ 90℃ 40℃ 50℃ 60℃ 70℃ 80℃ 90℃
DRP1
GAPDH
8505C-CON
8505C-KU-57788

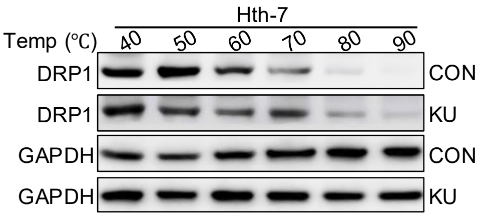
Hth-7
Temp (℃) 40 50 60 70 80 90
DRP1 CON
DRP1 KU
GAPDH CON
GAPDH KU

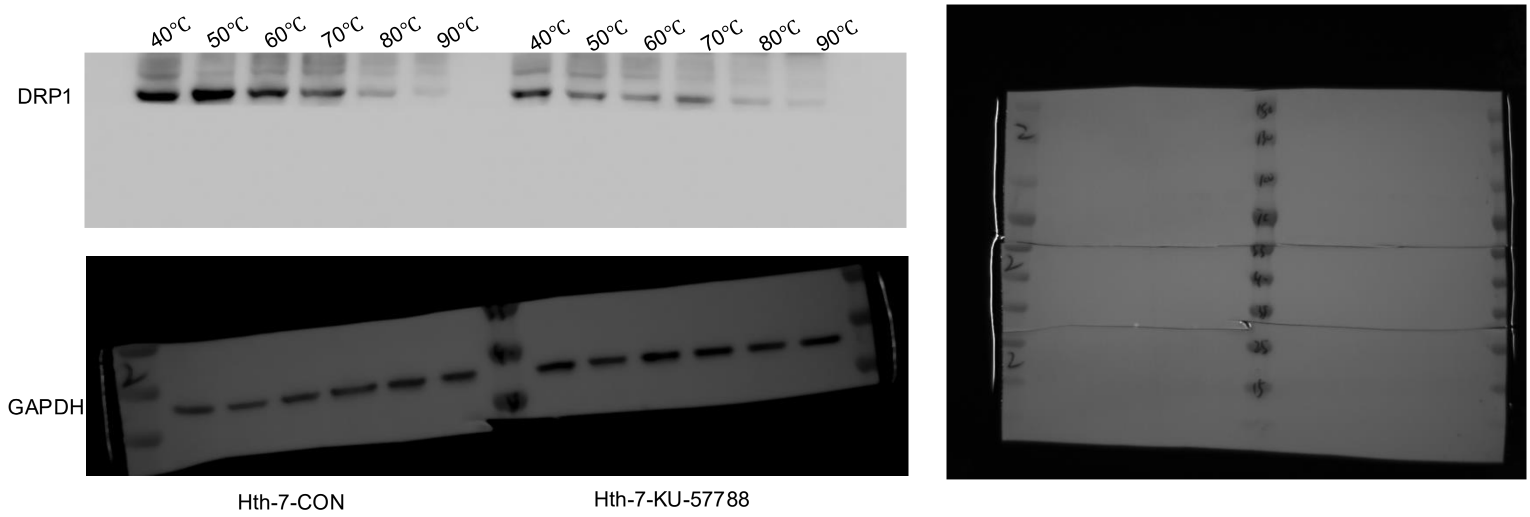
40℃ 50℃ 60℃ 70℃ 80℃ 90℃ 40℃ 50℃ 60℃ 70℃ 80℃ 90℃
DRP1
GAPDH
Hth-7-CON
Hth-7-KU-57788

Figue 7C

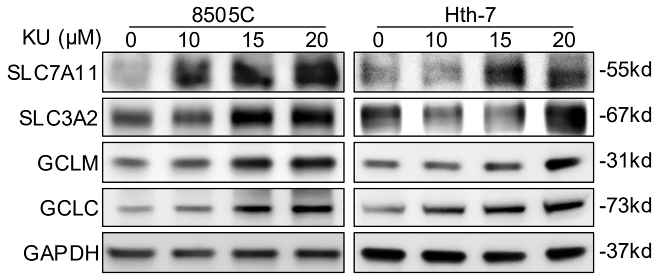
8505C
Hth-7
KU (μM) 0 10 15 20 0 10 15 20
SLC7A11 -55kd
SLC3A2 -67kd
GCLM -31kd
GCLC -73kd
GAPDH -37kd

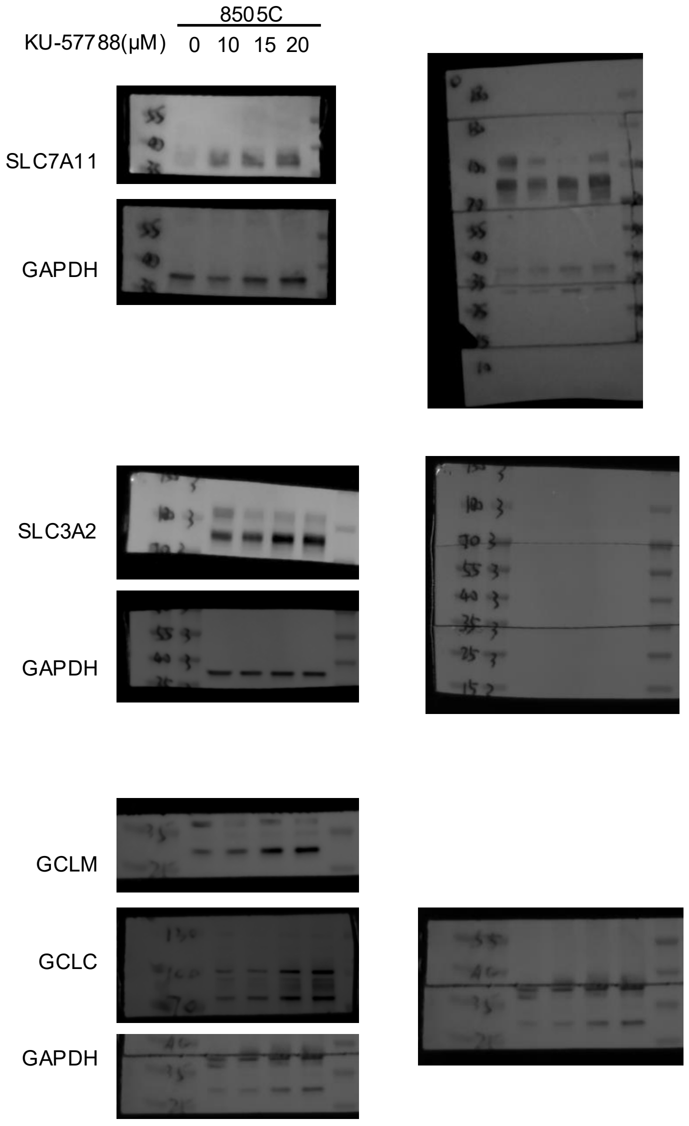
8505C
KU-57788(μM) 0 10 15 20
SLC7A11
GAPDH
SLC3A2
GAPDH
GCLM
GCLC
GAPDH

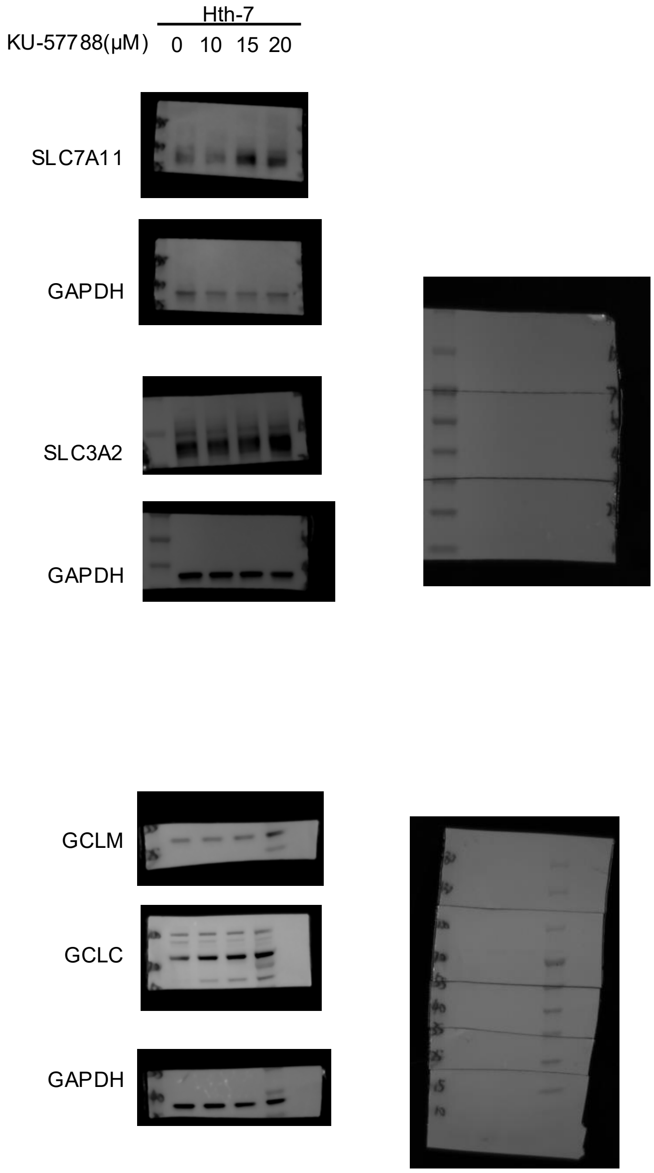
Hth-7
KU-57788(μM) 0 10 15 20
SLC7A11
GAPDH
SLC3A2
GAPDH
GCLM
GCLC
GAPDH

Figue 7D

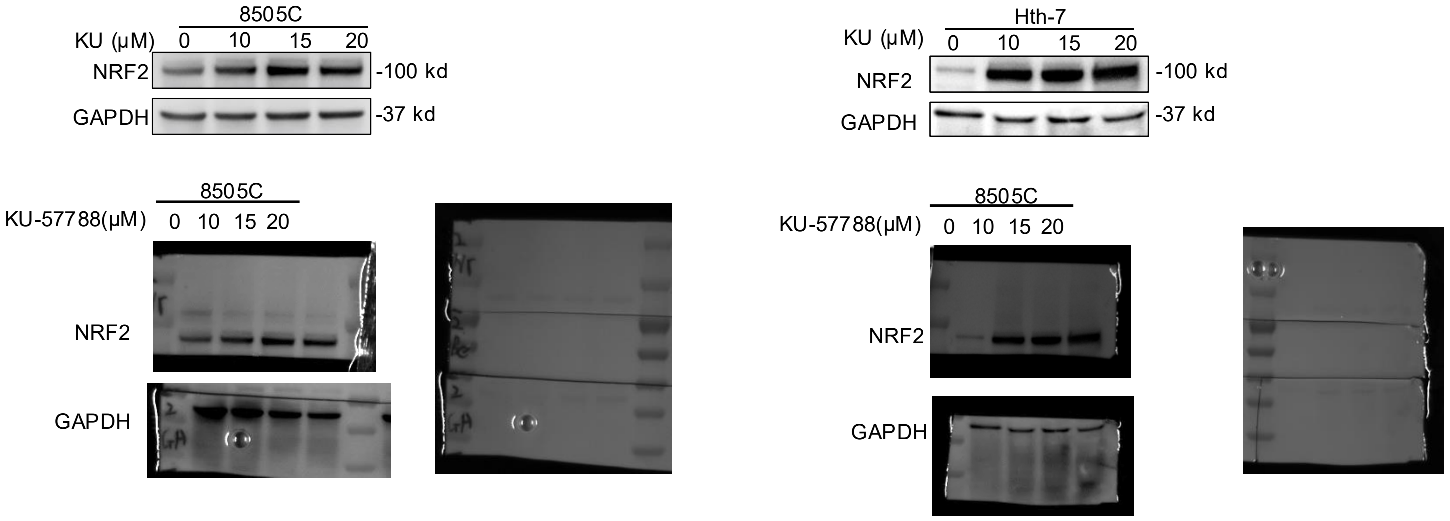
8505C
KU (μM) 0 10 15 20
NRF2
-100 kd
GAPDH
-37 kd
Hth-7
KU (μM) 0 10 15 20
NRF2
-100 kd
GAPDH
-37 kd
8505C
KU-57788(μM) 0 10 15 20
NRF2
GAPDH
8505C
KU-57788(μM) 0 10 15 20
NRF2
GAPDH

Figue 7J

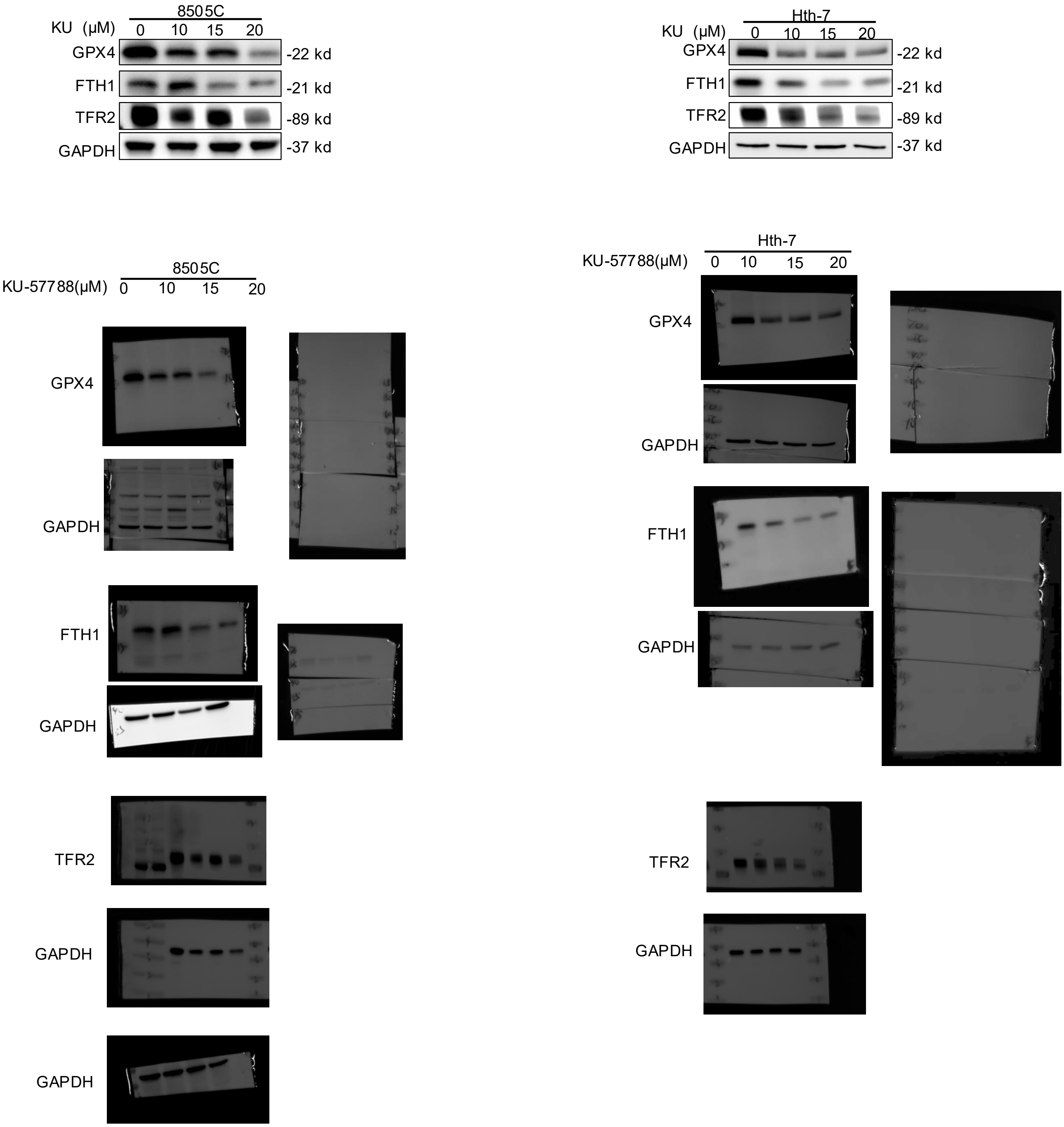
8505C
KU (μM) 0 10 15 20
GPX4 -22 kd
FTH1 -21 kd
TFR2 -89 kd
GAPDH -37 kd
Hth-7
KU (μM) 0 10 15 20
GPX4 -22 kd
FTH1 -21 kd
TFR2 -89 kd
GAPDH -37 kd
8505C
KU-57788(μM) 0 10 15 20
GPX4
GAPDH
FTH1
GAPDH
TFR2
GAPDH
GAPDH
Hth-7
KU-57788(μM) 0 10 15 20
GPX4
GAPDH
FTH1
GAPDH
TFR2
GAPDH

Figue S2

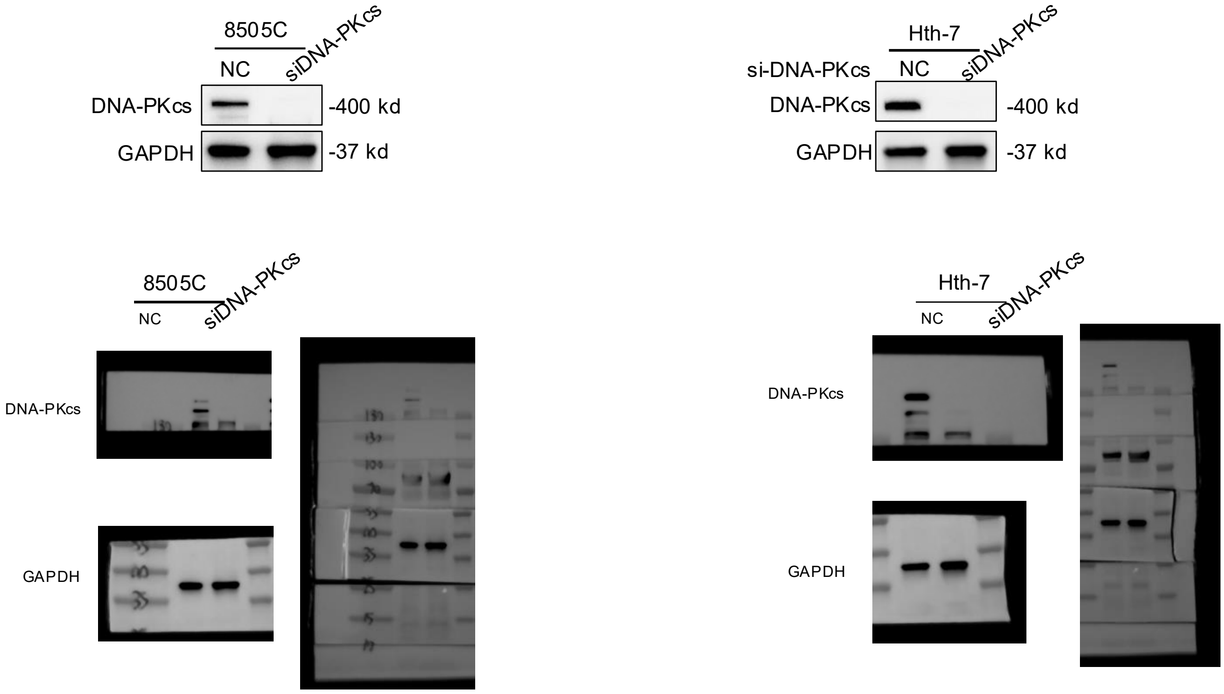
8505C
NC
siDNA-PKcs
DNA-PKcs
-400 kd
GAPDH
-37 kd
Hth-7
si-DNA-PKcs
NC
siDNA-PKcs
DNA-PKcs
-400 kd
GAPDH
-37 kd
8505C
NC
siDNA-PKcs
DNA-PKcs
GAPDH
Hth-7
NC
siDNA-PKcs
DNA-PKcs
GAPDH

Figue S6

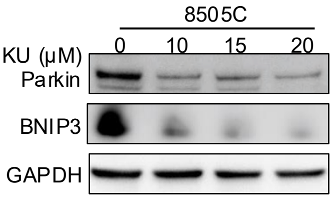
8505C
KU (μM)
0
10
15
20
Parkin
BNIP3
GAPDH

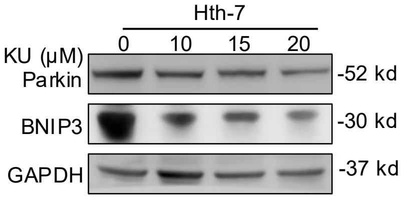
Hth-7
KU (μM)
0
10
15
20
Parkin
BNIP3
GAPDH
-52 kd
-30 kd
-37 kd

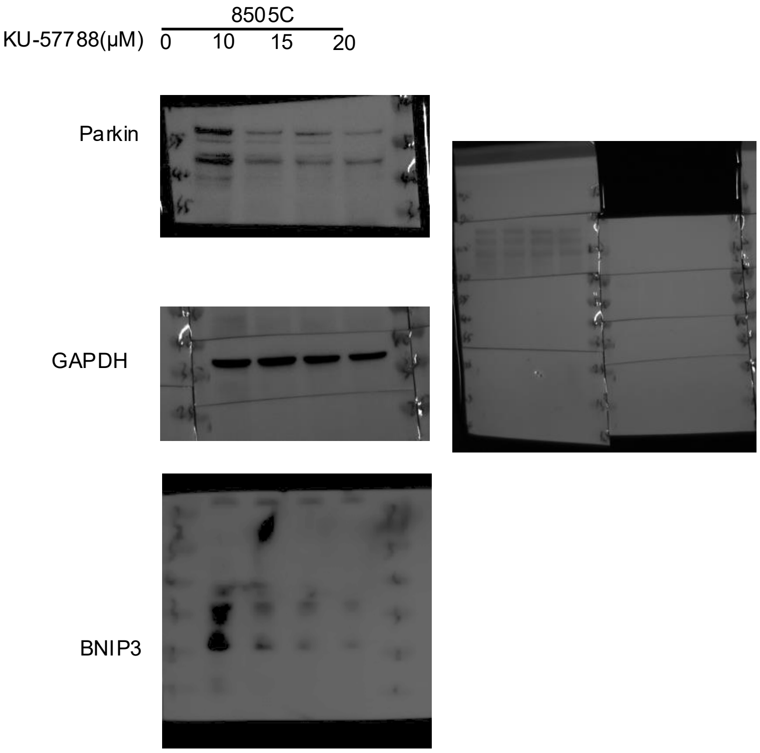
8505C
KU-57788(μM)
0
10
15
20
Parkin
GAPDH
BNIP3

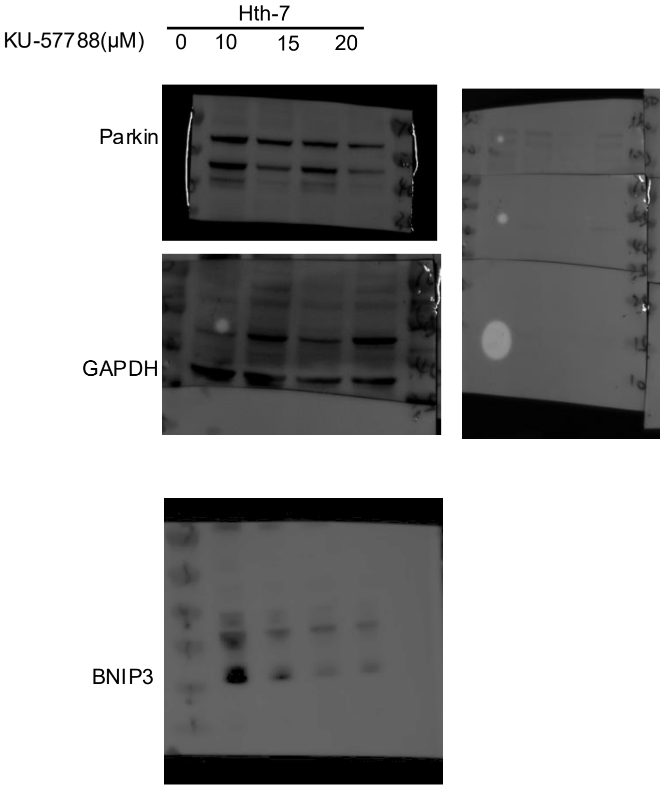
Hth-7
KU-57788(μM)
0
10
15
20
Parkin
GAPDH
BNIP3

Figue S8 A

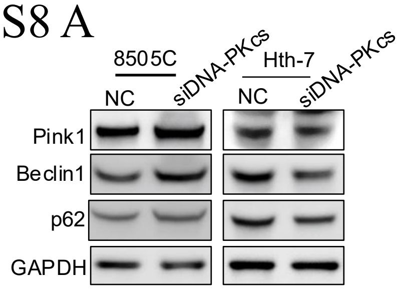
8505C
Hth-7
NC
siDNA-PKcs
NC
siDNA-PKcs
Pink1
Beclin1
p62
GAPDH

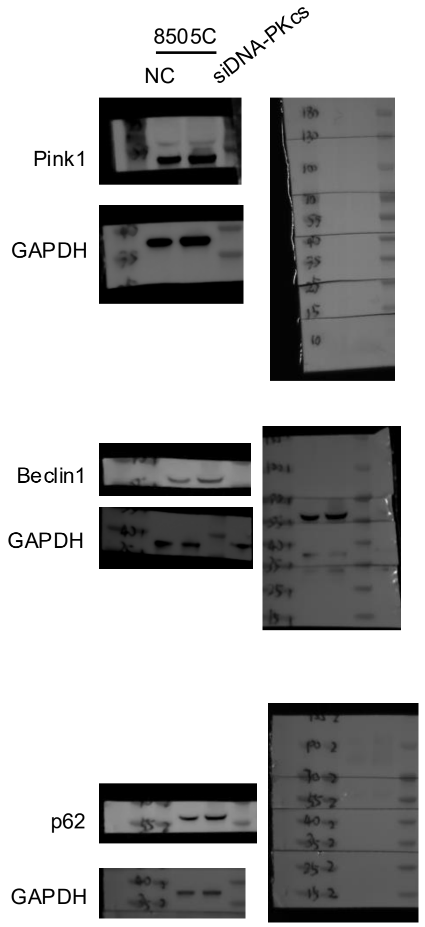
8505C
NC
siDNA-PKcs
Pink1
GAPDH
Beclin1
GAPDH
p62
GAPDH

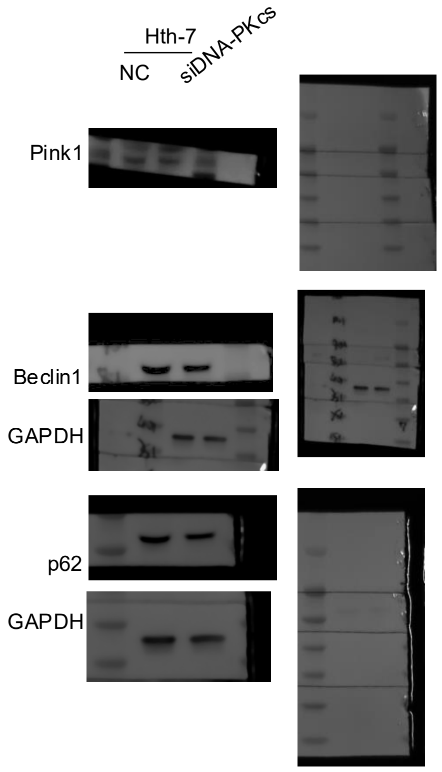
Hth-7
NC
siDNA-PKcs
Pink1
Beclin1
GAPDH
p62
GAPDH

Figue S8 B

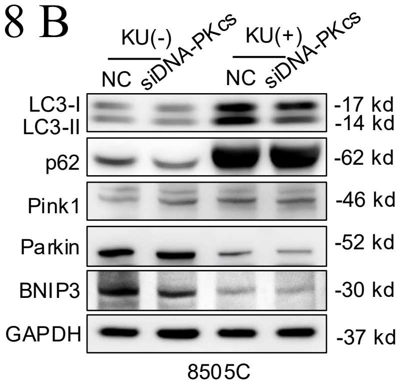
KU(-)
KU(+)
NC
siDNA-PKcs
LC3-I
LC3-II
p62
Pink1
Parkin
BNIP3
GAPDH
-17 kd
-14 kd
-62 kd
-46 kd
-52 kd
-30 kd
-37 kd
8505C

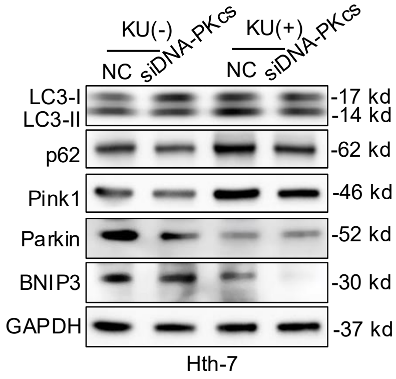
KU(-)
KU(+)
NC
siDNA-PKcs
LC3-I
LC3-II
p62
Pink1
Parkin
BNIP3
GAPDH
-17 kd
-14 kd
-62 kd
-46 kd
-52 kd
-30 kd
-37 kd
Hth-7

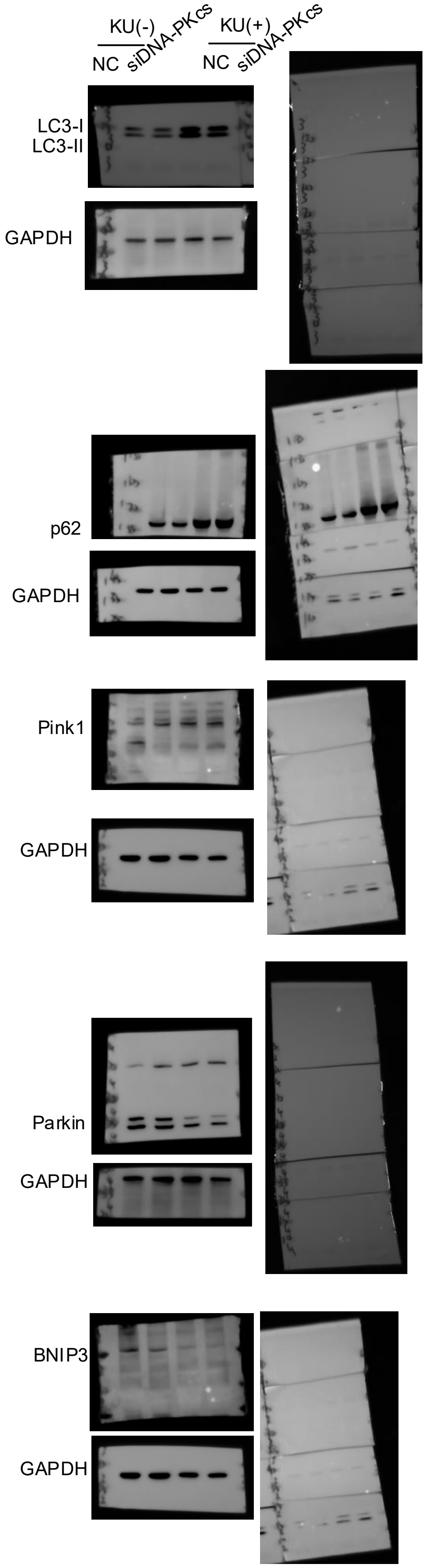
KU(-)
KU(+)
NC
siDNA-PKcs
LC3-I
LC3-II
GAPDH
p62
GAPDH
Pink1
GAPDH
Parkin
GAPDH
BNIP3
GAPDH

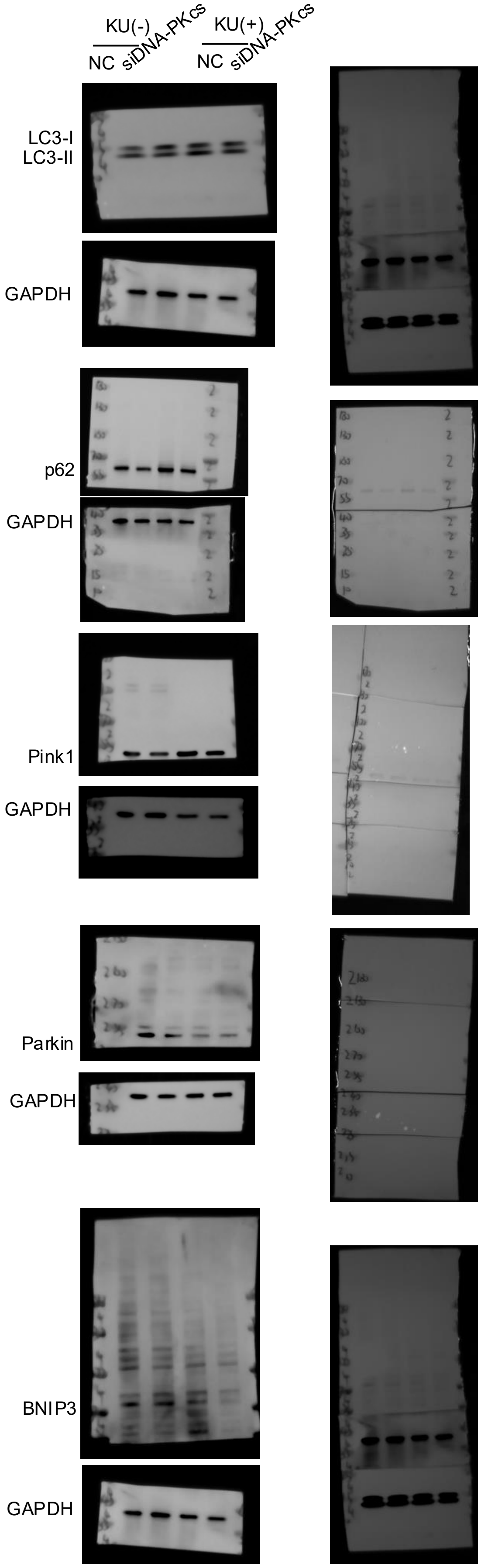
KU(-)
KU(+)
NC
siDNA-PKcs
LC3-I
LC3-II
GAPDH
p62
GAPDH
Pink1
GAPDH
Parkin
GAPDH
BNIP3
GAPDH

Figue S8 D

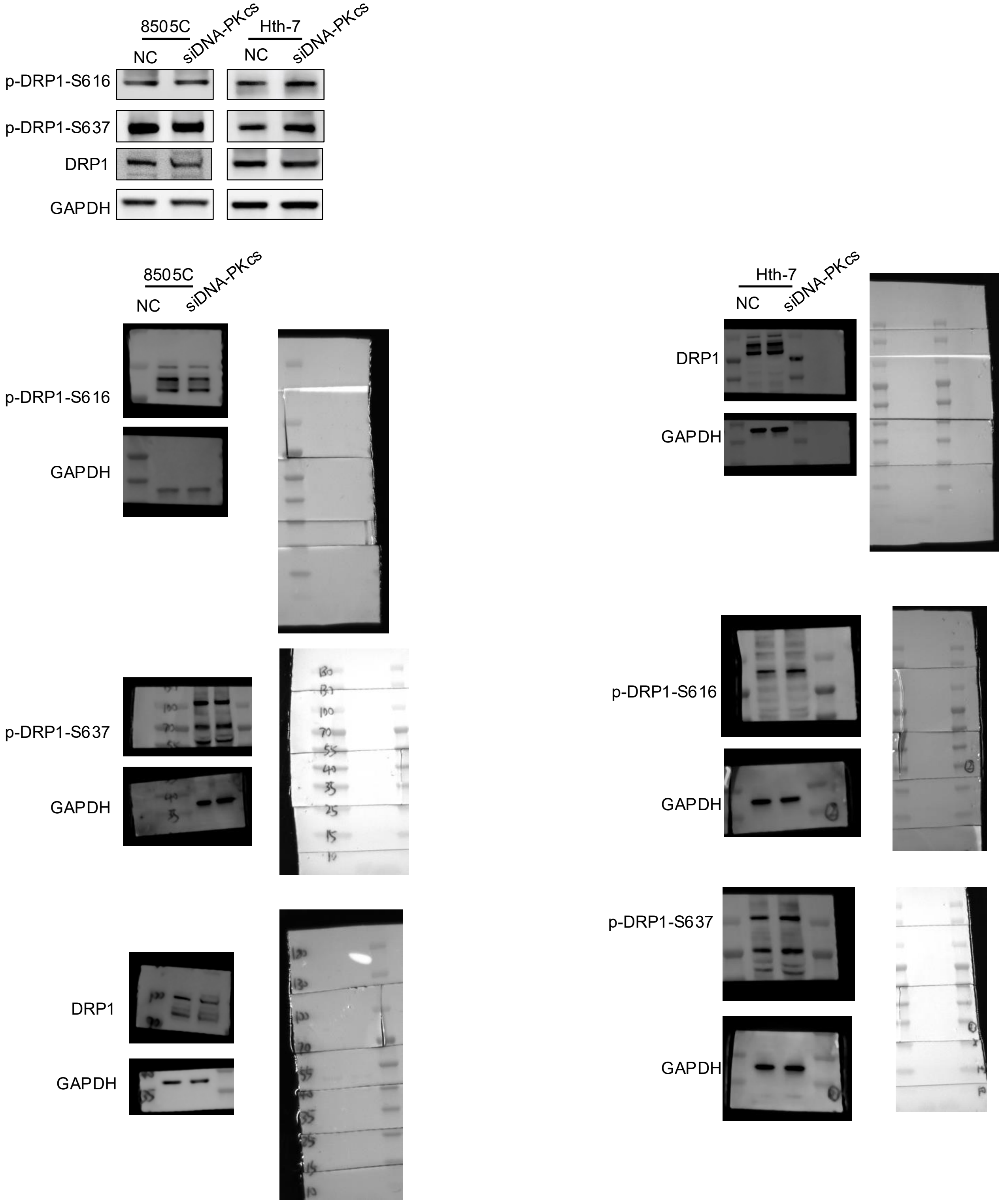
8505C
NC
siDNA-PKcs
Hth-7
NC
siDNA-PKcs
p-DRP1-S616
p-DRP1-S637
DRP1
GAPDH
8505C
NC
siDNA-PKcs
p-DRP1-S616
GAPDH
p-DRP1-S637
GAPDH
DRP1
GAPDH
Hth-7
NC
siDNA-PKcs
DRP1
GAPDH
p-DRP1-S616
GAPDH
p-DRP1-S637
GAPDH

Figue S9

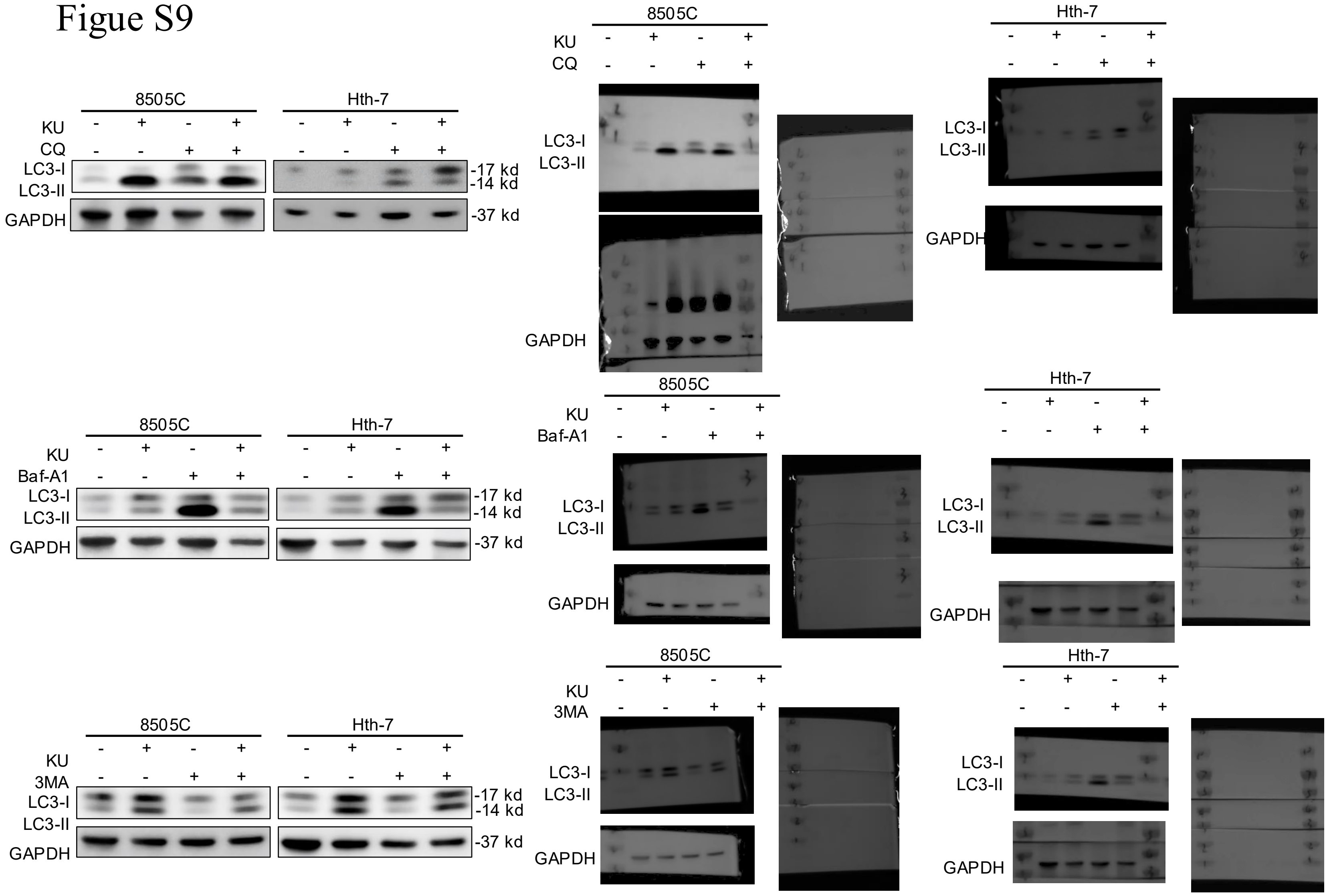

Figue S11 A

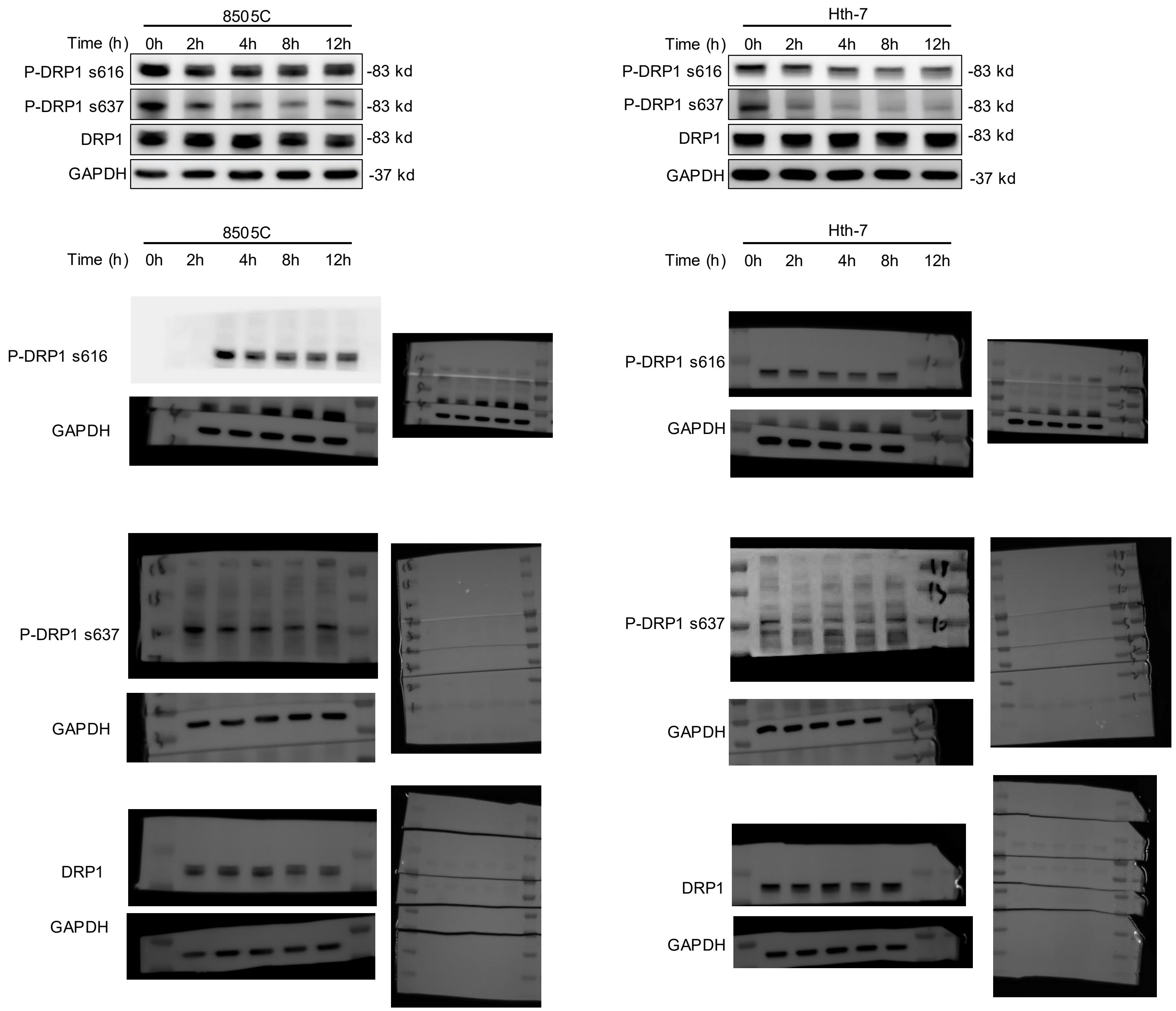

Figue S11 C

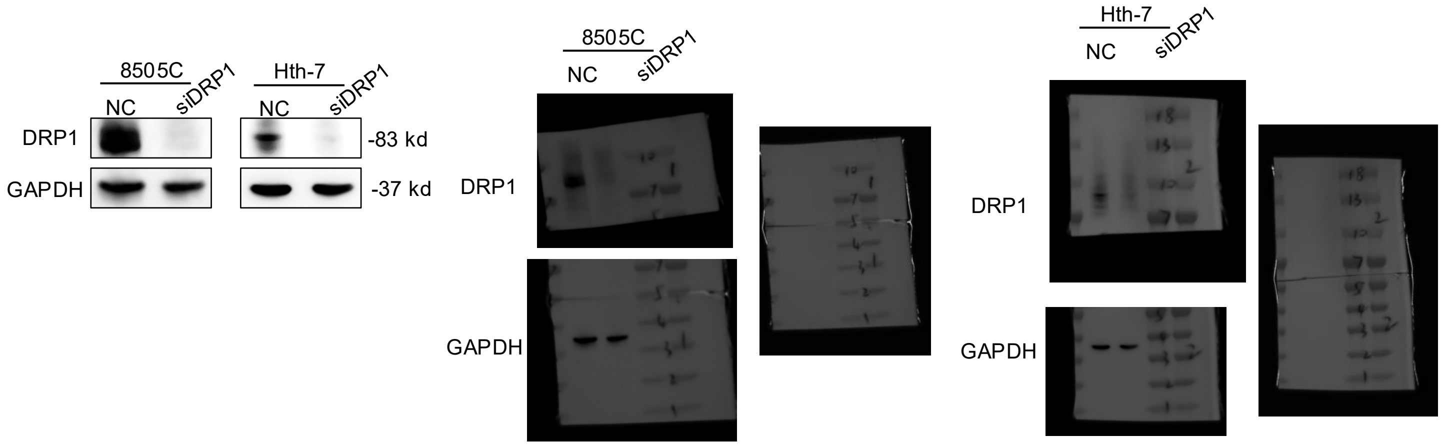

Figue S15 C

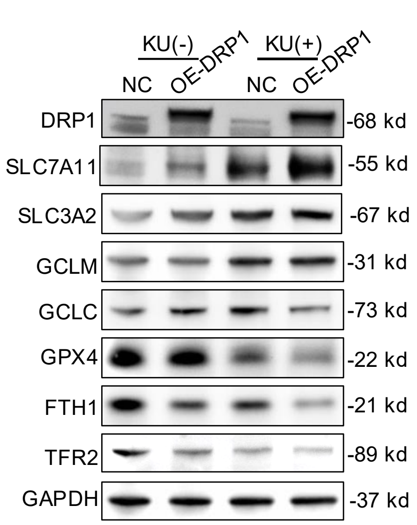

KU(-)
KU(+)
NC
OE-DRP1
NC
OE-DRP1
DRP1
-68 kd
SLC7A11
-55 kd
SLC3A2
-67 kd
GCLM
-31 kd
GCLC
-73 kd
GPX4
-22 kd
FTH1
-21 kd
TFR2
-89 kd
GAPDH
-37 kd

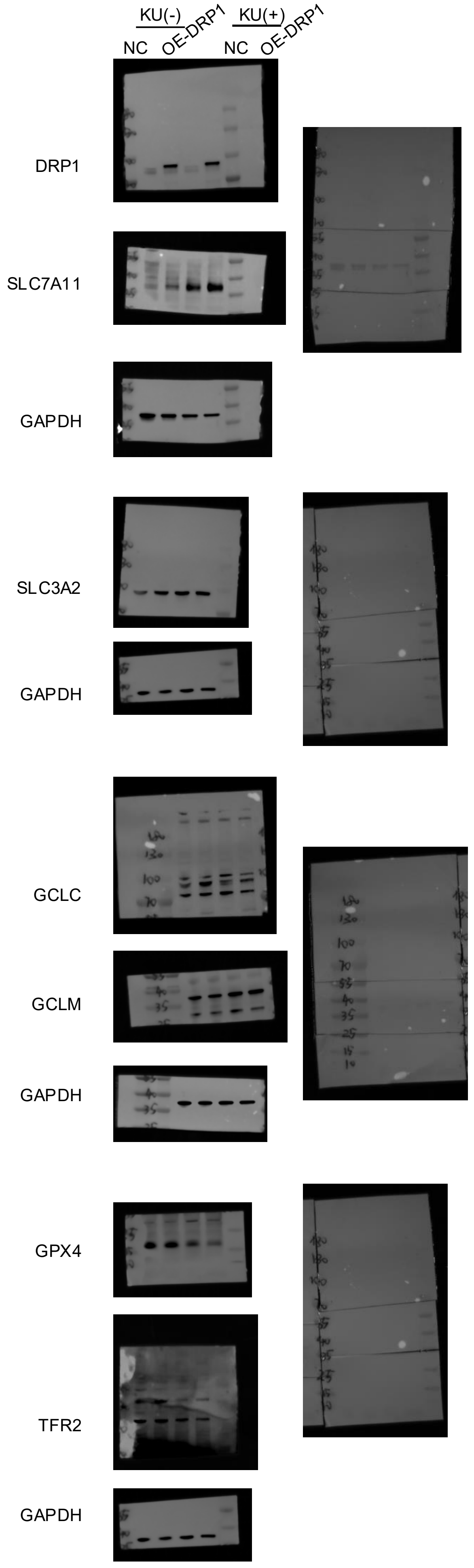

KU(-)
KU(+)
NC
OE-DRP1
NC
OE-DRP1
DRP1
SLC7A11
GAPDH
SLC3A2
GAPDH
GCLC
GCLM
GAPDH
GPX4
TFR2
GAPDH
