# Supplementary material for: Direct coupling and protective activation of DRP1 by the DNA-PKcs inhibitor KU-57788 synergizes with ferroptosis in anaplastic thyroid cancer cells
Source: Cell Death Dis. 2026 Apr 28;17(1):570. doi: 10.1038/s41419-026-08595-3 (PMC13265778; doi:10.1038/s41419-026-08595-3)
Supplement: Supplementary file 2 — Supplement figure [file 41419_2026_8595_MOESM2_ESM.docx]

**Supplementary Materials for**

Direct coupling and protective activation of DRP1 by the DNA-PKcs inhibitor KU-57788 synergizes with ferroptosis in anaplastic thyroid cancer cells

Lingling Ding†, Changtian Yin†, Yehao Guo†, Qiang Geng†, Wanwan He, Yawen Guo, Jinpeng Wen, Aoni Zhou, Jieyu Luo, Xinxin Ren, Jiajie Xu, Renhao Ou, Ruonan Jia, Jiaxin Tian, Yuchen Wang, Yefeng Cai, Wenzhen Wang, Haifeng Xu, Lei Zhu, Minghua Ge*, Guowan Zheng*, Chuanming Zheng*

*Corresponding authors.

E-mail address: mingdoc@163.com, zhengguowan@hmc.edu.cn, geminghua@hmc.edu.cn.

**†**These authors made equal contributions to this work.

**Supplementary Figure**

**
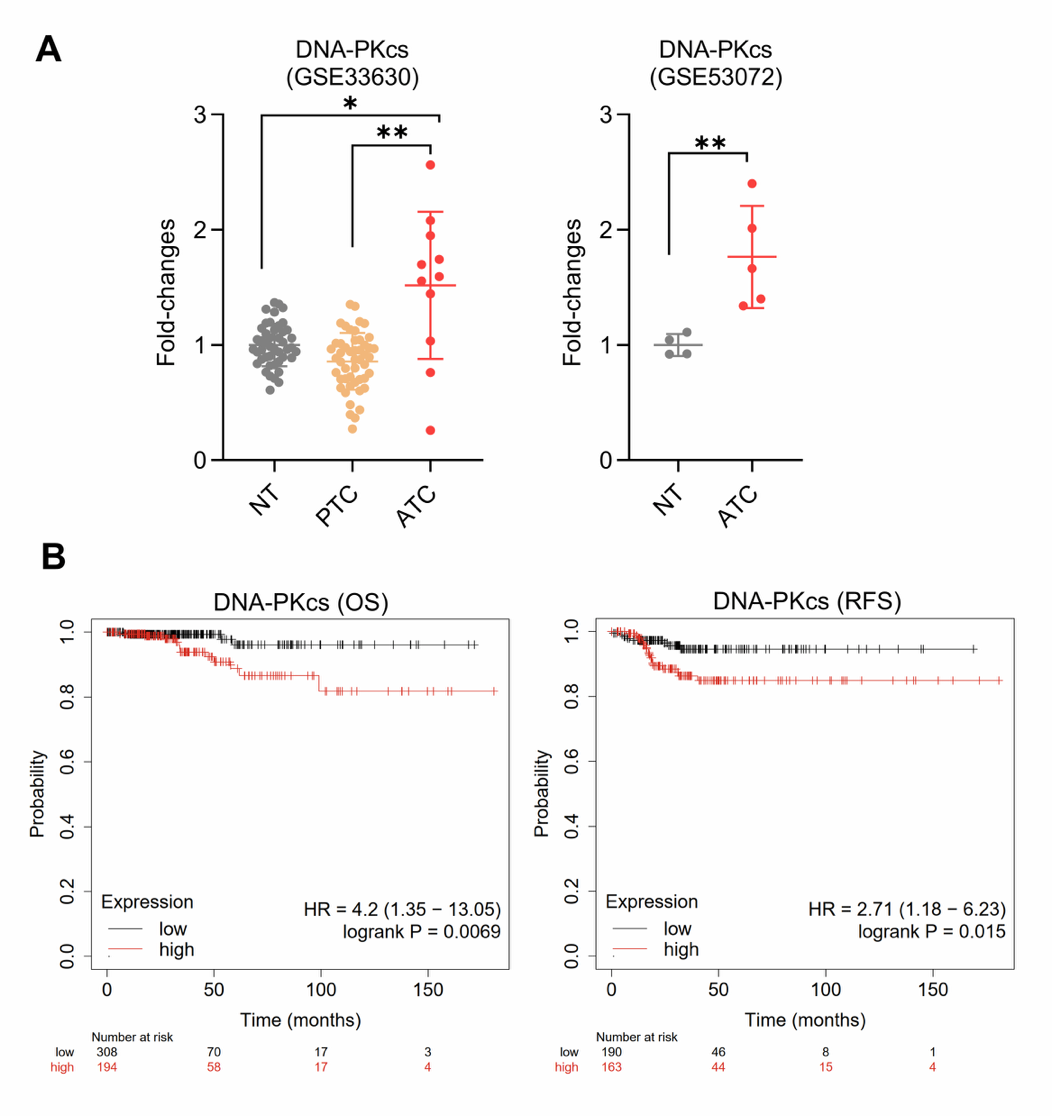
**

**Supplementary Fig. 1 DNA-PKcs is specifically upregulated in ATC. A** The expression analysis of DNA-PKcs was performed on the GSE33630 chip data, which included 11 cases of ATC, 49 cases of PTC, and 45 cases of adjacent cancer tissues, as well as the GSE53072 chip data, which included 5 cases of ATC and 4 cases of adjacent cancer tissues. **B** Kaplan-Meier Plotter database (https://kmplot.com/analysis/index.php?p=service&cancer=pancancer_rnaseq#) was used to analyze the expression level of DNA-PKcs in relation to overall survival (OS) and relapse-free survival (RFS) in thyroid cancer patients. **p*< 0. 05, ***p* < 0. 01, ns, *p* > 0.05

**
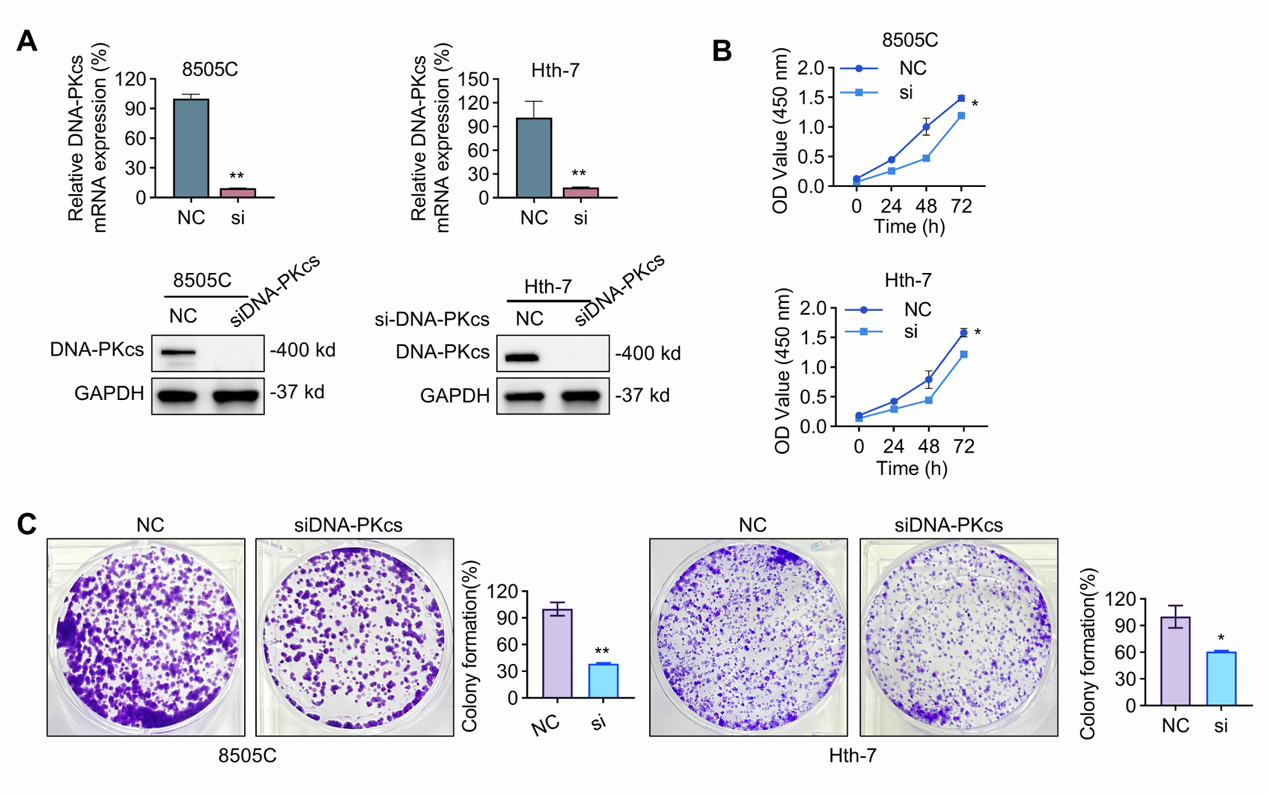
**

**Supplementary Fig. 2 Knockdown of DNA-PKcs effectively inhibits the proliferation and colony formation of ATC cells. A** qPCR and western blot technique were used to investigate the changes in mRNA and protein levels of DNA-PKcs in ATC cells 8505C and Hth-7 after knockdown of DNA-PKcs. **B** The CCK-8 method was used to continuously examine the cell proliferation of ATC cells 8505C and Hth-7 at 24 h, 48 h, and 72 h after knockdown of DNA-PKcs. **C** A colony formation assay was used to examine the colony formation ability of ATC cells 8505C and Hth-7 after knockdown of DNA-PKcs. **p* < 0. 05, ***p* < 0. 01, ns, *p* > 0.05.


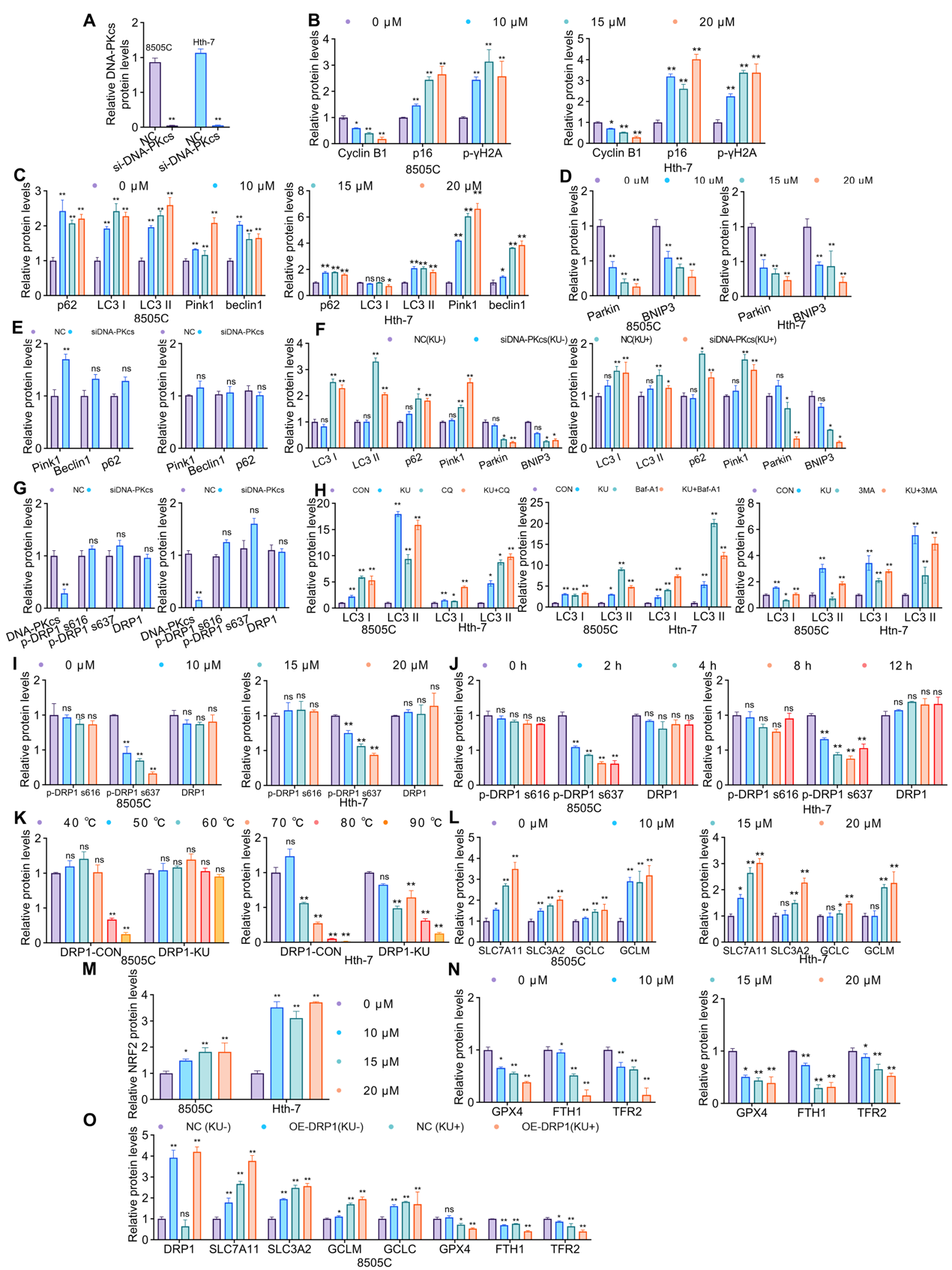


**Supplementary Fig. 3 Quantitative analysis of the Western blot results.** Band intensities were quantified and normalized to those of GAPDH. **A** Quantitative analysis of the Fig. S2A. **B** Quantitative analysis of the Fig. 1D. **C** Quantitative analysis of the Fig. 2C. **D** Quantitative analysis of the Fig. S6. **E, F and G** Quantitative analysis of the Fig. S8A, B and D. **H** Quantitative analysis of the Fig. S9. **I** Quantitative analysis of the Fig. 4A. **J** Quantitative analysis of the Fig. S11A. **K** Quantitative analysis of the Fig. 5E. **L and M** Quantitative analysis of the Fig. 7C and D. **N** Quantitative analysis of the Fig. 7J. **O** Quantitative analysis of the Fig. S15C. Data are presented as mean ± SEM (n=3). Statistical significance was determined by one-way ANOVA (**p* < 0.05, ***p* < 0.01, ns, *p* > 0.05. versus the control group).


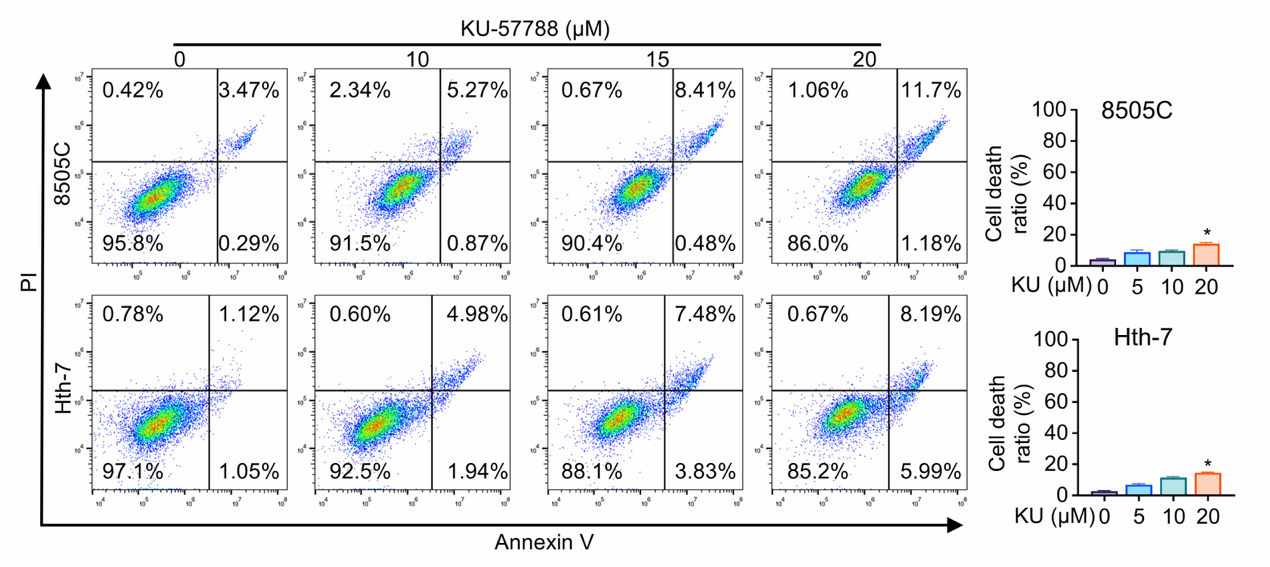


**Supplementary Fig. 4 KU-57788 mildly induces cell death in ATC cells.** Cell deaths of KU-57788 treated 8505C and Hth-7 (0, 10,15, and 20 μM, for 24 h) was measured by flow cytometry and the cell death ratio was presented. **p* < 0. 05.

**
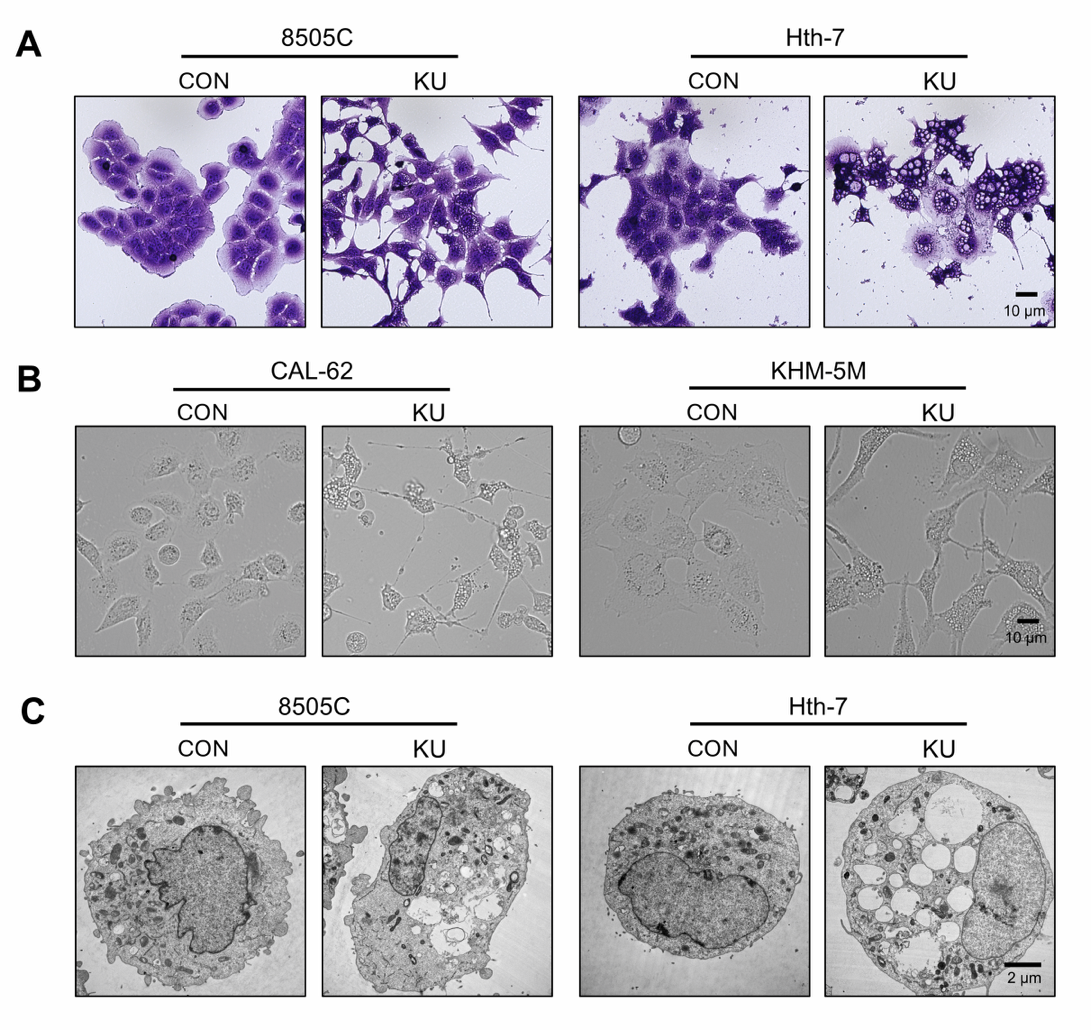
**

**Supplementary Fig. 5 KU-57788 induces morphological changes in ATC cells. A and B** Characteristic morphology of 8505C and Hth-7 cells induced by KU-57788 (0 and 10 μM for 24 h), captured by a phase-contrast optical microscope (Evos M7000). **C** Transmission electron microscopy was used to observe mitochondrial morphological changes in 8505C and Hth-7 cells treated with KU-57788 (0 and 10 μM for 24 h).

**
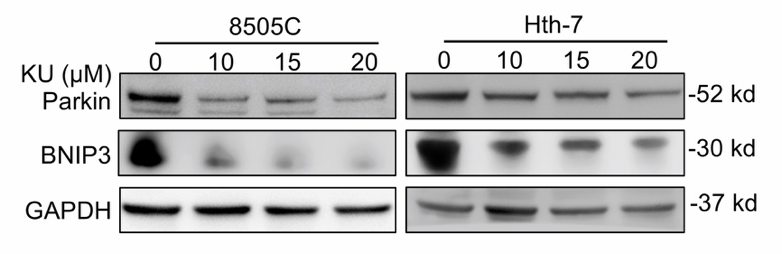
**

**Supplementary Fig. 6** Western blot technique was used to investigate the changes in protein levels of Parkin, BNIP3, and GAPDH in ATC cells 8505C and Hth-7, after KU-57788 (0, 10, 15 and 20 μM for 24 h) respectively

**
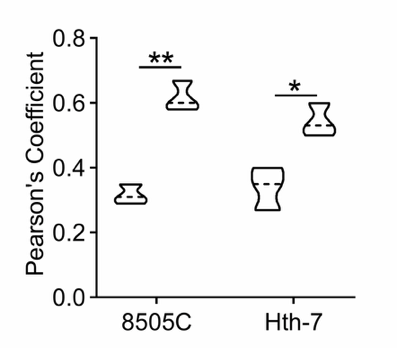
**

**Supplementary Fig. 7** Quantification of colocalization of LC3B and HSP60 using Pearson’s coefficient. Images were analyzed using ImageJ and Jacob Plugin. Pearson’s coefficient values are plotted. Data are shown as mean ± SD for n = 3, analyzed by one-way ANOVA using the Holm-Sidak method. ***p*< 0.01.

**
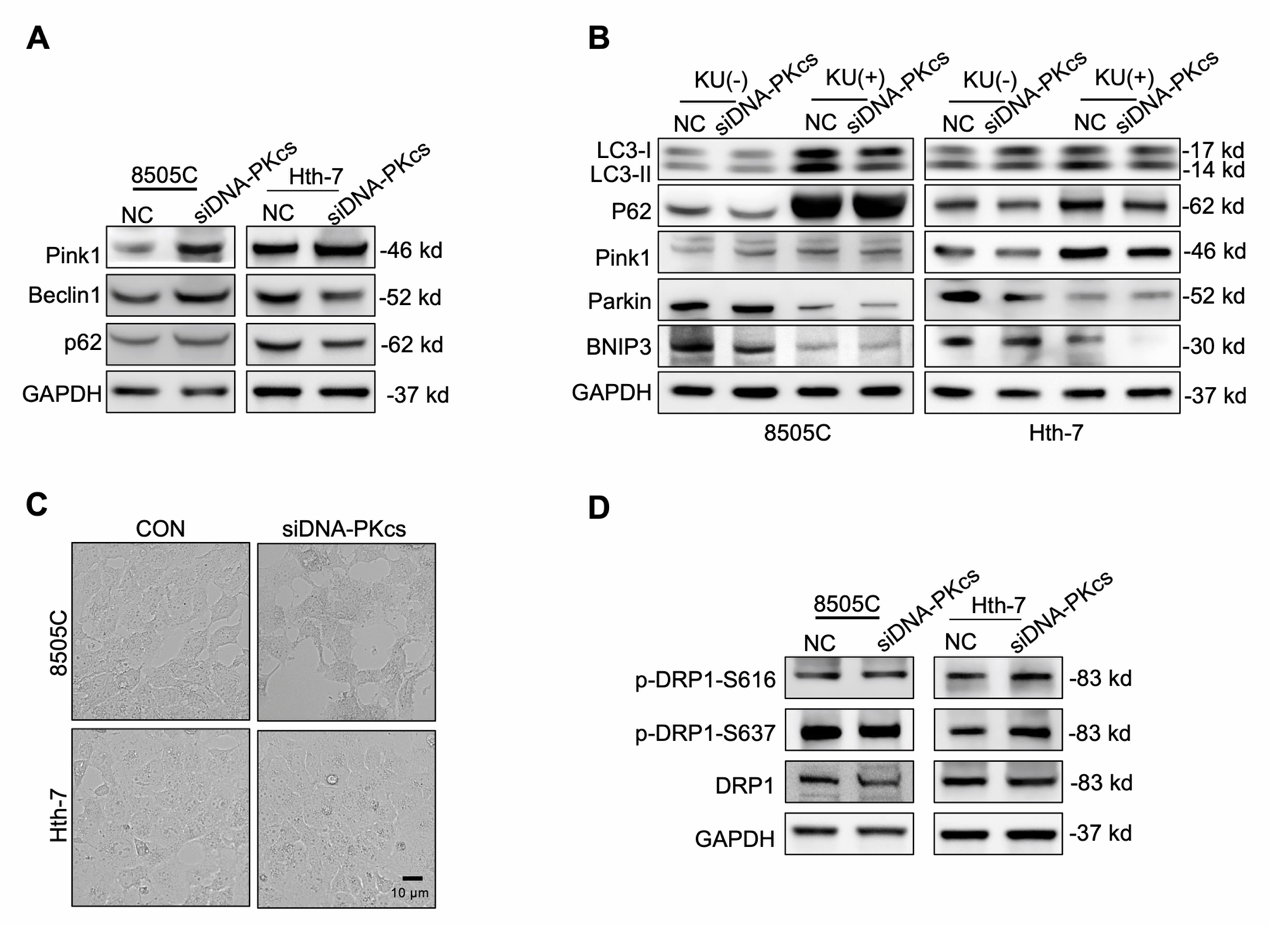
**

**Supplementary Fig. 8 Knockdown of DNA-PKcs has minimal effect on mitophagy, DRP1, and its phosphorylation status in ATC cells. A** Western blot technique was used to investigate the changes in protein levels of Pink1, Beclin1, p62, and GAPDH in ATC cells 8505C and Hth-7 after knockdown of DNA-PKcs. **B** Western blotting to analyze the protein levels of LC3, p62, Pink1, Parkin, BNIP3, and GAPDH in ATC cells (8505C and Hth-7) following DNA-PKcs knockdown and treatment with or without KU-57788 (0 and 20 μM for 24 h). **C** Characteristic morphology of 8505C and Hth-7 cells after knockdown of DNA-PKcs by using a phase-contrast optical microscope (Evos M7000). **D** Western blot assay was used to investigate the changes in protein levels of p-DRP1-S616, p-DRP1-S637, DRP1, and GAPDH in ATC cells 8505C and Hth-7 after knockdown of DNA-PKcs.


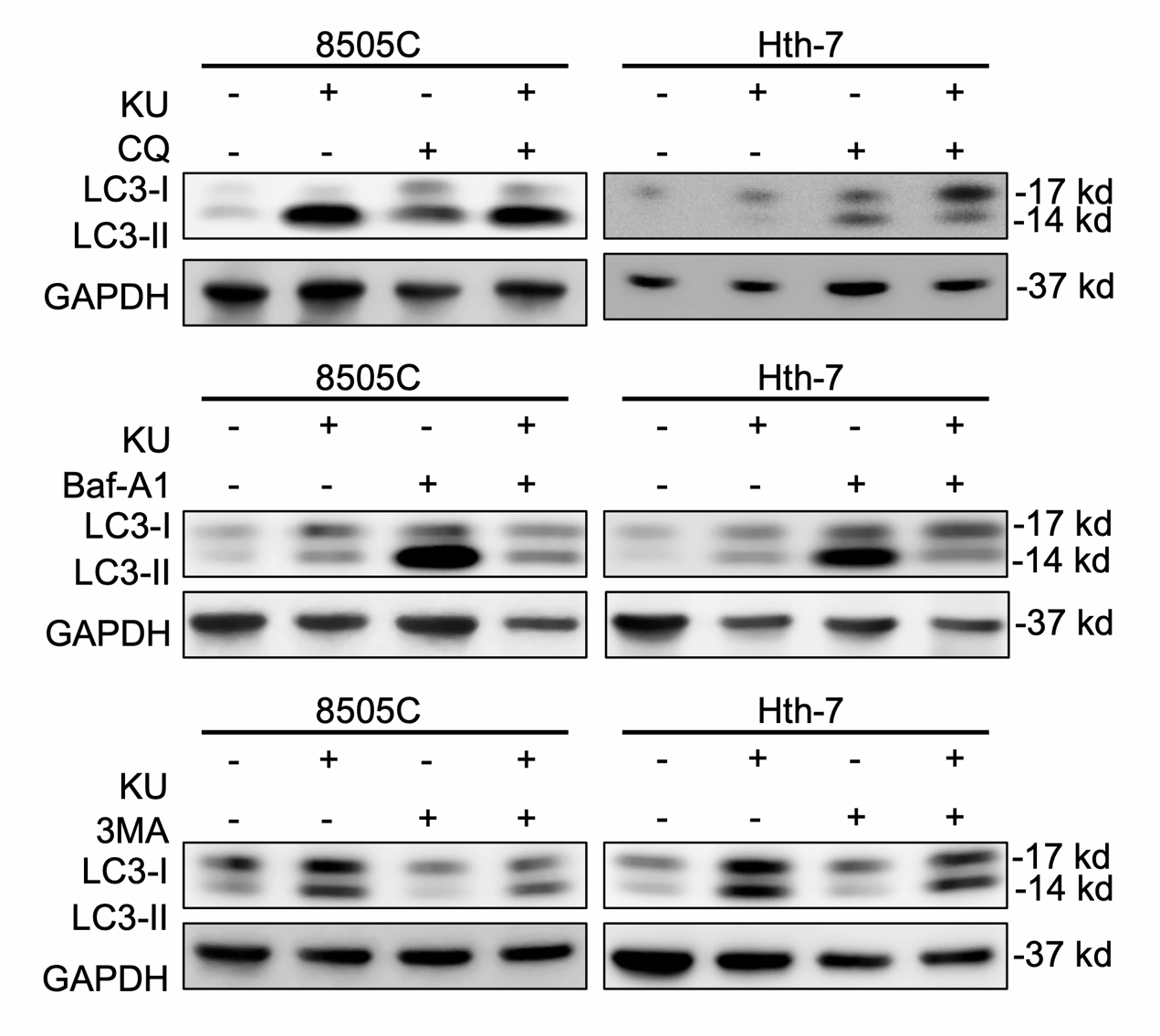


**Supplementary Fig. 9 KU-57788 activates autophagy as a stress response in ATC cells.** Western blot analysis was used to measure the protein levels of LC3 and GAPDH in 8505C and Hth-7 cells after treatment with CQ (10 μM, pre-treated for 2 hours)，BafA1 (100 nM, pre-treated for 2 hours), and 3-MA (3 mM, pre-treated for 2 hours), in combination with KU-57788 (20 μM for 24 hours).

**
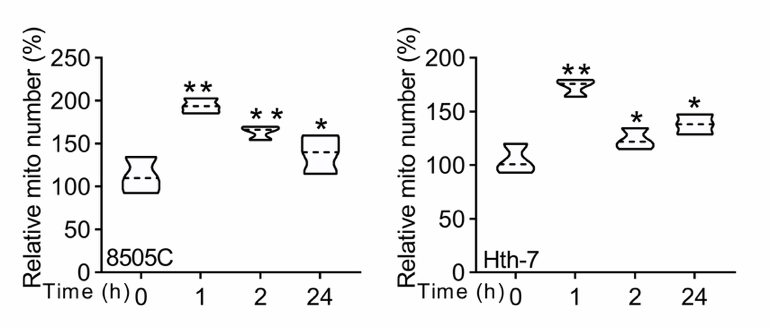
**

**Supplementary Fig. 10** Quantification of mitochondrial number (mito number) from Fig. 3C. **p* < 0. 05, ***p* < 0. 01.


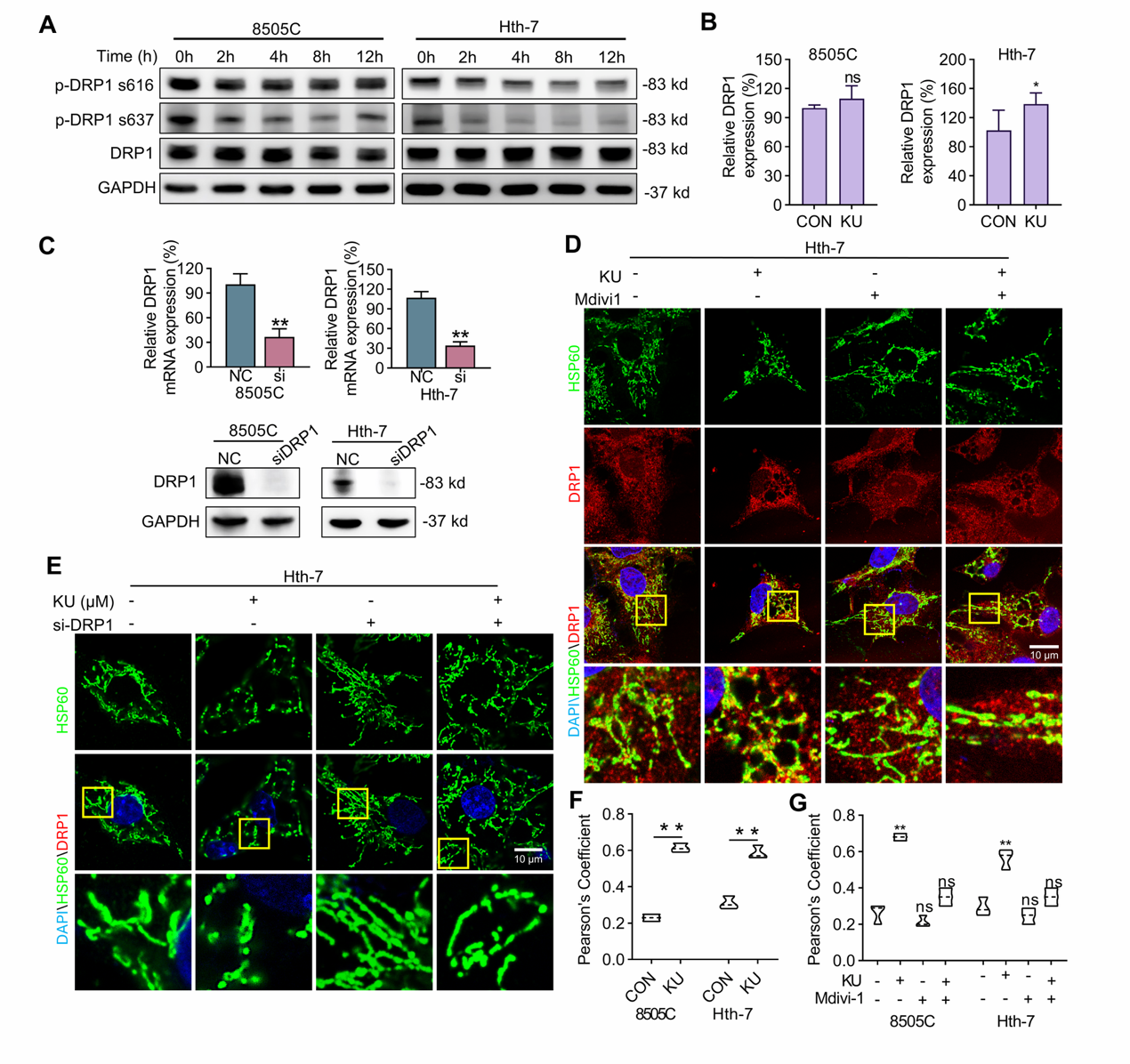


**Supplementary Fig. 11 KU-57788-mediated activation of DRP1 exerts a protective effect on ATC cells. A** Western blot was used to measure p-DRP1 s616, p-DRP1 s637, DRP1, and GAPDH protein levels in 8505 C and Hth-7 cells, after KU-57788 (0, and 20 μM for 24 h) for 0 h, 2 h, 4 h, 8 h,12 h. **B** mRNA change of DRP1 were measure after after treating with KU-57788(0 and 20 μM) for 24 h by qPCR. **C** qPCR and western blot technique were used to investigate the changes in mRNA and protein levels of DNA-PKcs in ATC cells 8505C and Hth-7 after knockdown of DRP1. **D** After knock-down DRP1, confocal laser was used to observe mitochondrial morphological changes in Hth-7, treated with KU-57788 (0 and 10 μM for 24 h), where mitochondrial were stained by HSP60 antibody. **E** Confocal laser was used to observe mitochondrial morphological changes in Hth-7, treated with KU-57788 (0 and 10 μM) and Mdivi-1 (0 and 10 μM) for 24 h, where mitochondrial were stained by HSP60 and DRP1antibody. **F** Quantification of colocalization of DRP1 and HSP60 of Fig. 4B using Pearson’s coefficient. Images were analyzed using ImageJ and Jacob Plugin. Pearson’s coefficient values are plotted. **G** Quantification of colocalization of DRP1 and HSP60 of Fig. 4E and Fig. S11D using Pearson’s coefficient. Images were analyzed using ImageJ and Jacob Plugin. Pearson’s coefficient values are plotted. **p* < 0. 05, ***p* < 0. 01, ns, *p* > 0.05.


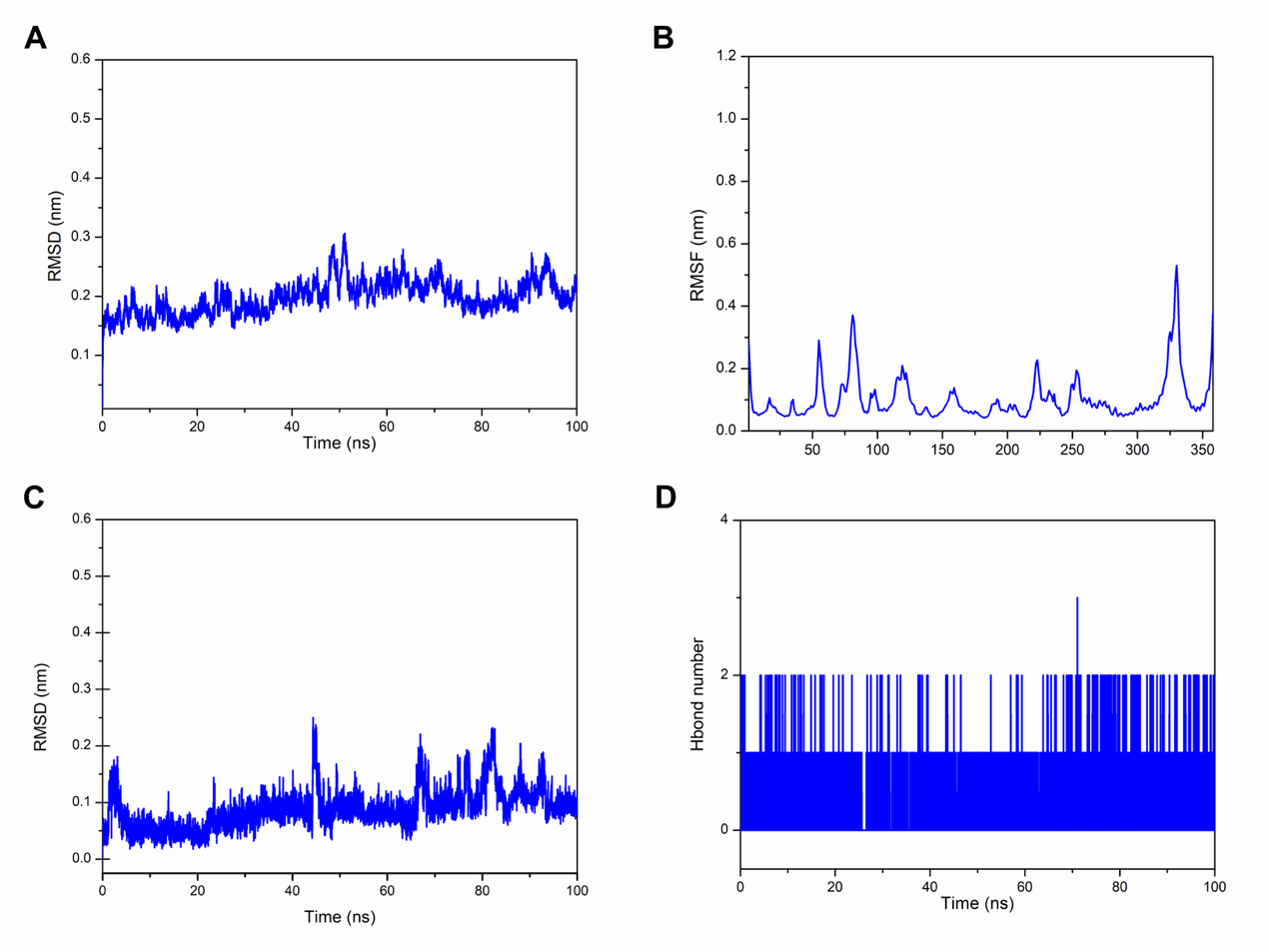


**Supplementary Fig. 12 A** Root Mean Square Fluctuation (RMSF) of the protein structure. **B** Root Mean Square Fluctuation (RMSF) of the ligand during molecular dynamics simulation. **C** Root Mean Square Deviation (RMSD) of the ligand during molecular dynamics simulation. **D** Hydrogen bond network in the ligand-protein complex.

**
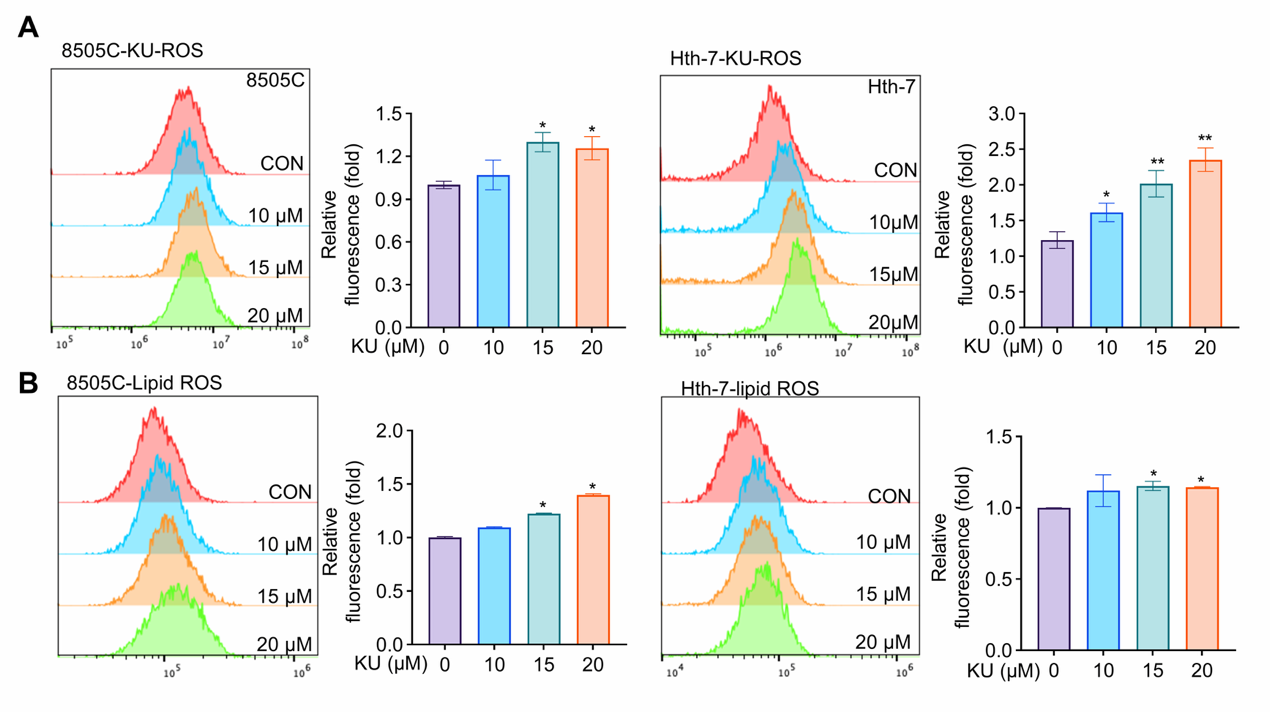
**

**Supplementary Fig. 13 Changes in ROS and lipid ROS after treating ATC cells with Ku-57788 and Mdivi-1. A and B** The levels of reactive oxygen species (ROS) and Iipid ROS were measured following treatment of KU-57788 (0, 10, 15, and 20 µM) for 24 hours in 8505C and Hth-7 cells. **p* < 0. 05, ***p* < 0. 01.

**
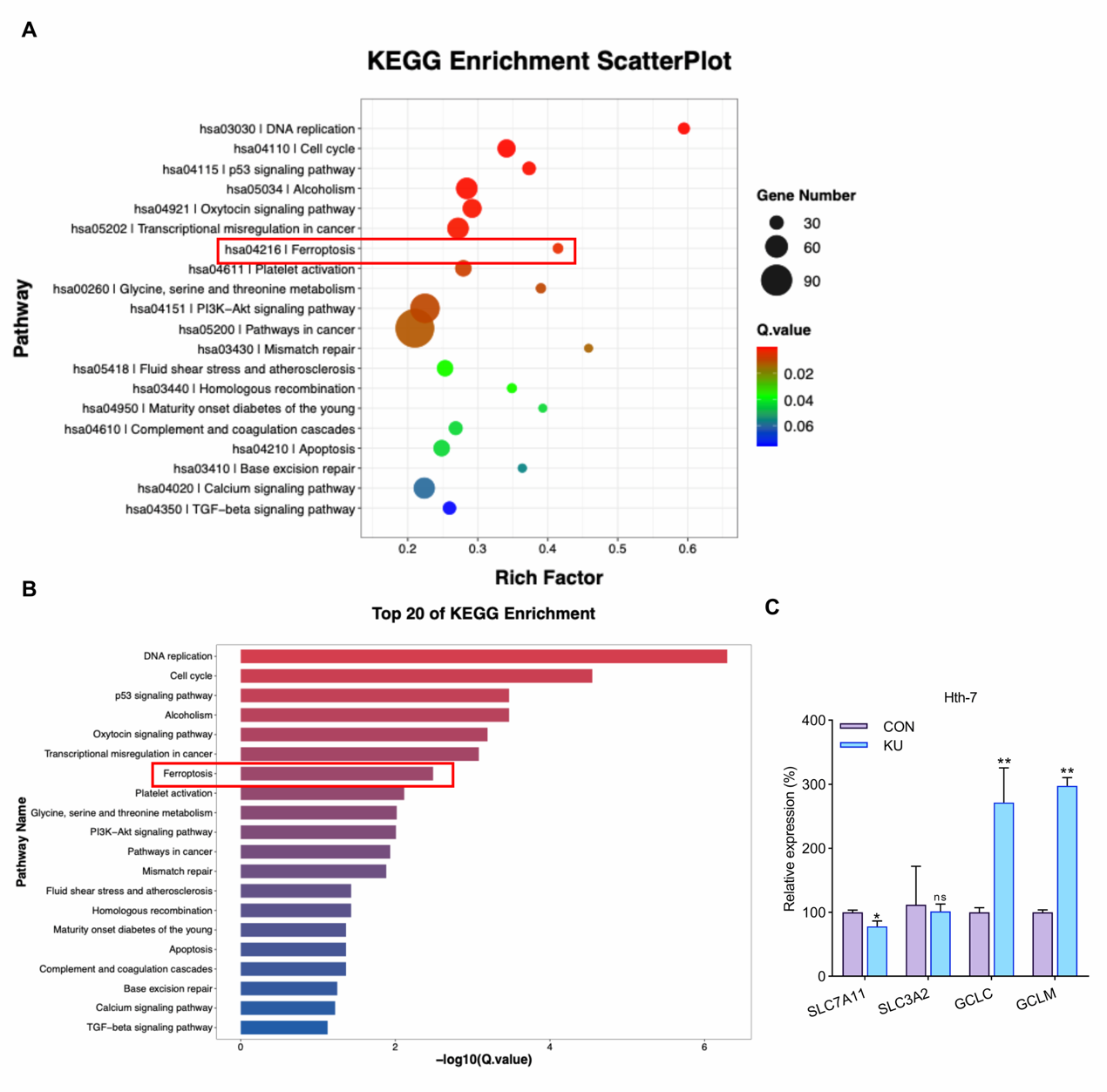
**

**Supplementary Fig. 14** **KEGG Enrichment and gene expression changes related to ferroptosis. A** KEGG enrichment scatter plot. **B** Top 20 KEGG pathway enrichment analysis. **C** RNA expression changes of ferroptosis-related genes SLC7A11, SLC3A2, GCLC, and GCLM in Hth-7 cells after treatment with KU-57788 (0 and 20 µM for 24h). **p* < 0. 05, ***p* < 0. 01, ns, *p* > 0.05.


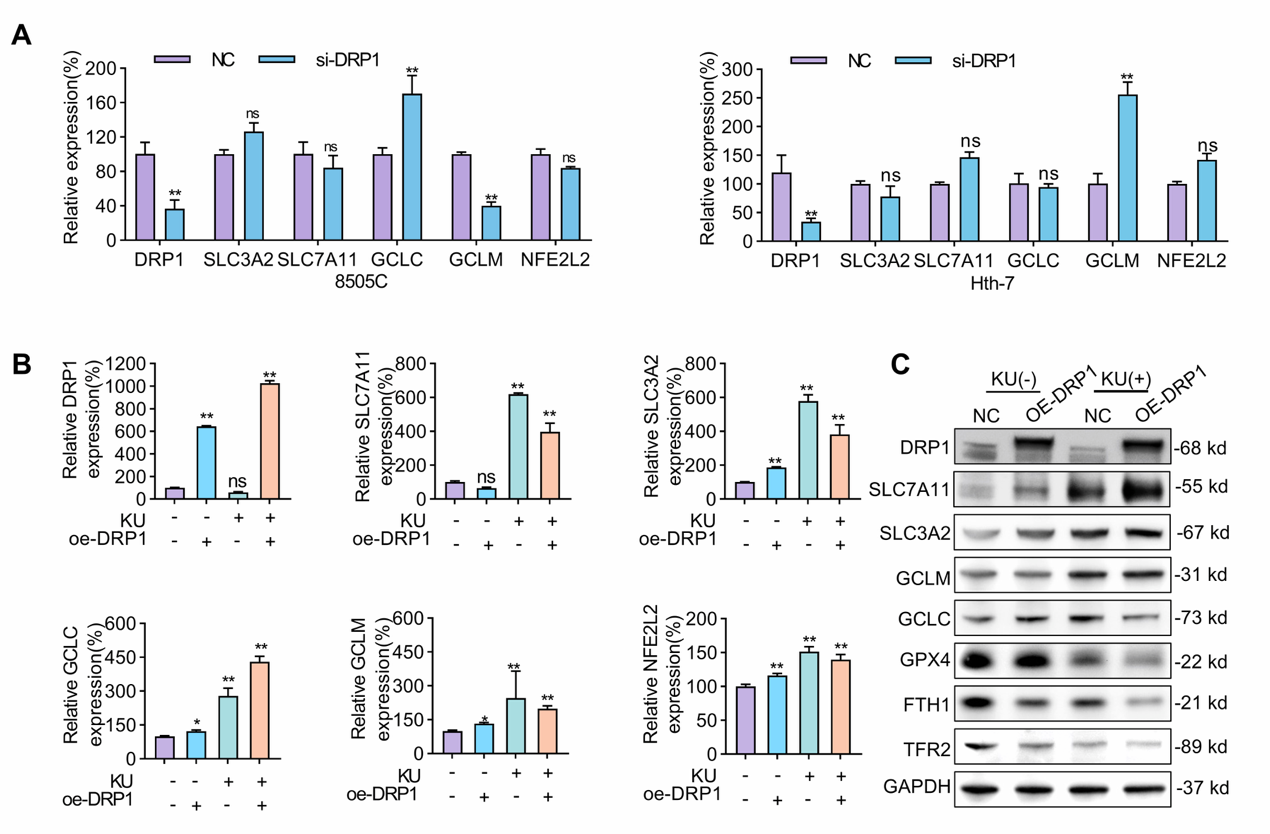


**Supplementary Fig. 15 DRP1 upregulation induced by KU-57788 serves as the activating mechanism for the NRF2/SLC7A11/GSH pathway. A** RT-qPCR was used to investigate the changes in mRNA levels of DRP1, SLC3A2, SLC7A11, GCLC, GCLM and NFE2L2 in ATC cells 8505C and Hth-7 after knockdown of DRP1 in 8505C and Hth-7. **B** qPCR used to investigate the changes in mRNA levels of DRP1, SLC3A2, SLC7A11, GCLC, GCLM and NFE2L2 in ATC cells 8505C after overexpress of DRP1 in 8505C. **C** Western blot used to investigate the changes in mRNA levels of DRP1, SLC3A2, SLC7A11, GCLC, GCLM and NFE2L2 in ATC cells 8505C and Hth-7 after overexpress of DRP1 in 8505C and treatment with or without KU-57788 (0 and 20 μM for 24 h). **p* < 0. 05, ***p* < 0. 01, ns, *p* > 0.05.


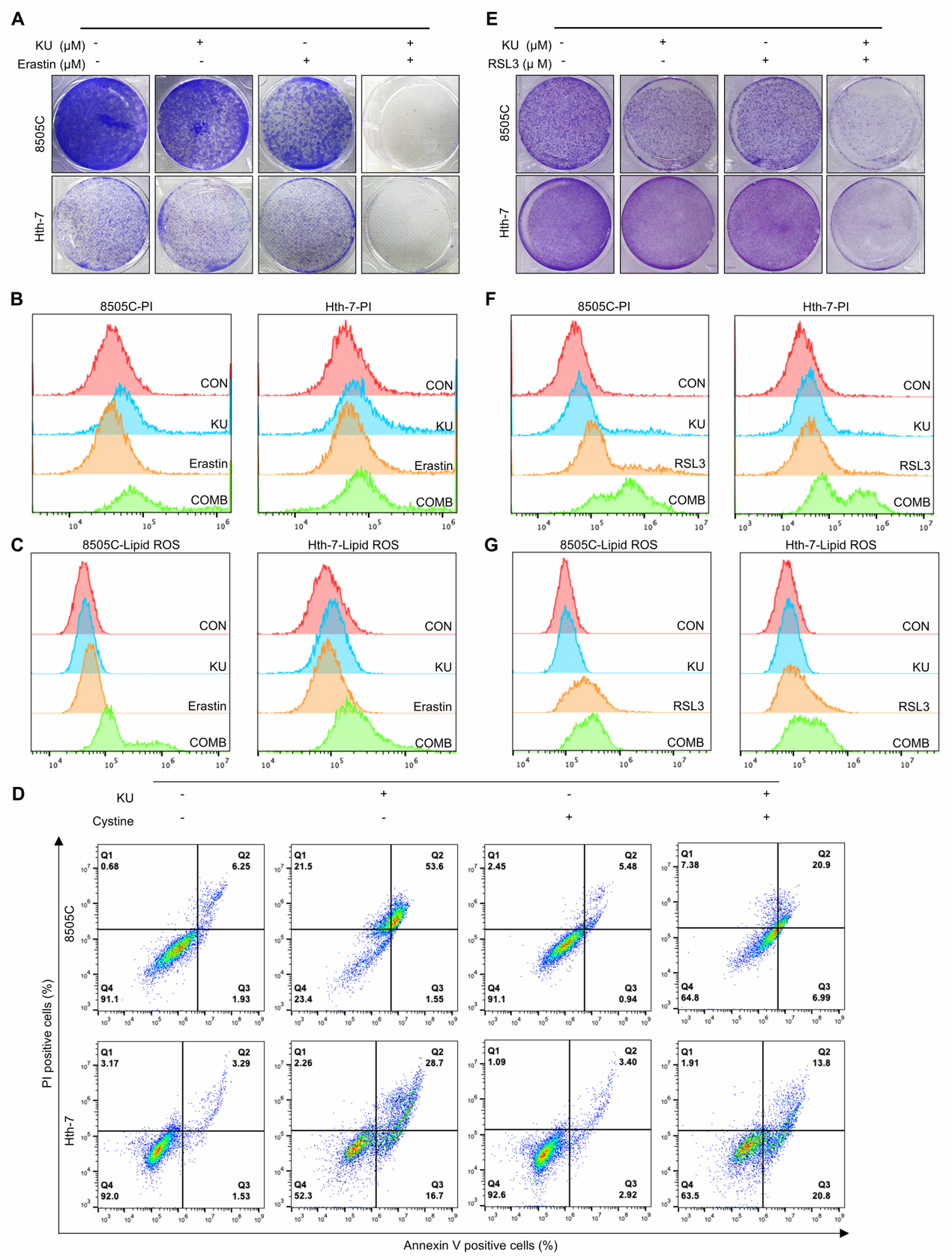


**Supplementary Fig. 16 KU-57788 synergizes with ferroptosis inducers to enhance cell death. A, B and C** The clone formation, cell death and lipid ROS at the indicated concentrations of KU-57788 (0 and 10 μM) with or without Erastin (5 μM) in 8505C and Hth-7 cell. **D** Cell deaths of 8505C and Hth-7 after treated of KU-57788 (0 and 10 μM, for 24 h) with or without cystine was measured by flow cytometry and the ratio presented. The groups are as follows: Control (no KU-57788, no cystine); Cystine Deprivation (with KU-57788, no cystine); KU Treatment (no KU-57788, with cystine); KU-57788 + Cystine (with KU-57788, with cystine). **E, F and G** The clone formation, cell death and lipid ROS at the indicated concentrations of KU-57788 (0 and 10 μM) with or without RSL3 (2.5 μM) in 8505C and Hth-7 cell.


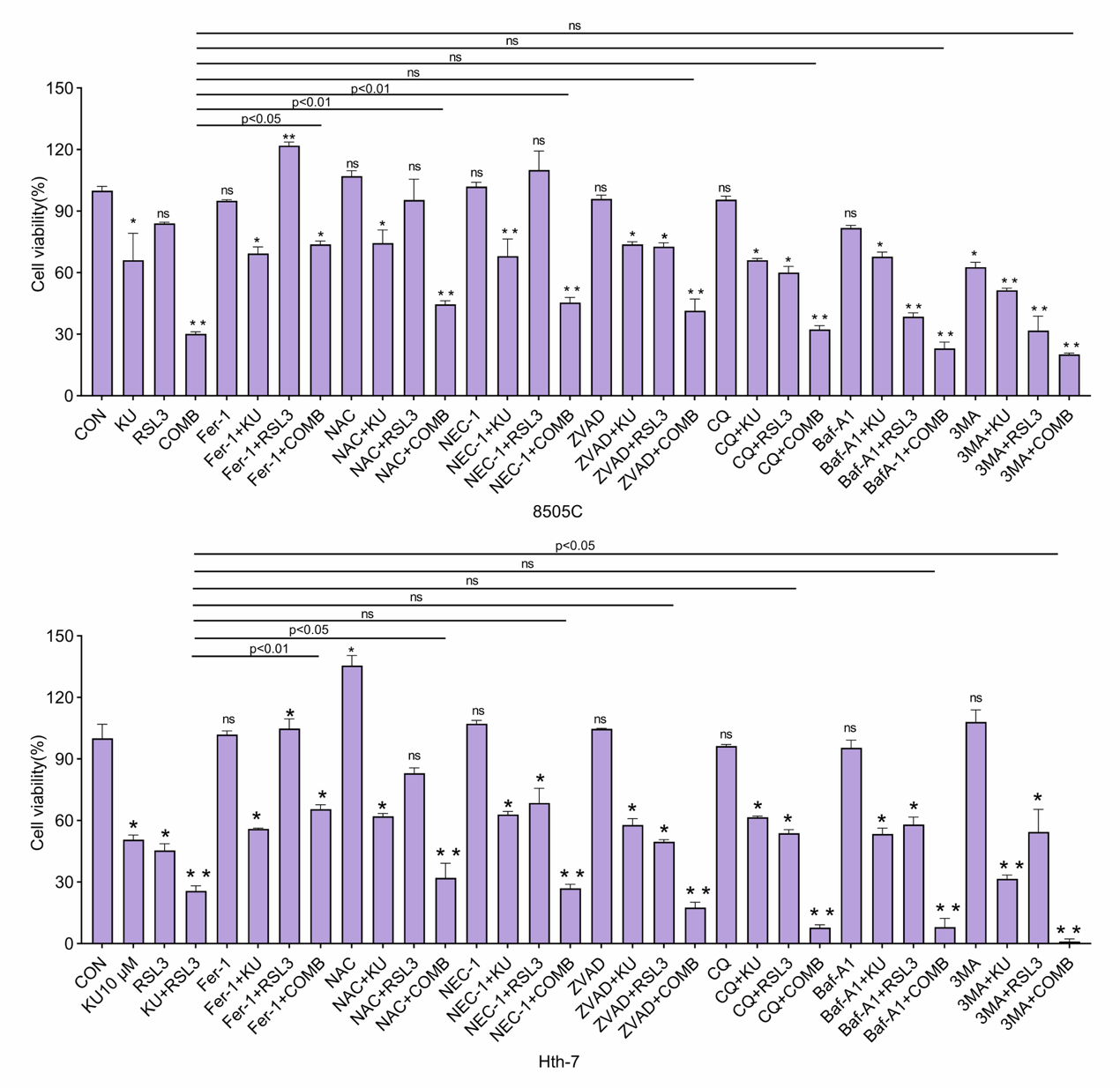


**Supplementary Fig. 17** **Inhibition of ferroptosis rescues KU and RSL3-induced cell death.** 8505C and Hth-7 cells were pre-treated with the ferroptosis inhibitor Fer-1 (1 μM), the NAC (2.5 mM), Nec-1 (10 μM), ZVAD (40 μM), the autophagy inhibitor CQ (10 μM), Baf-A1 (100 nM) and 3-MA (3 mM), pre-treated for 2 hours, followed by co-treatment with KU-57788 and RSL3. Cell viability was subsequently assessed, compared with the control group, **p* < 0. 05, ***p* < 0. 01, ns, *p* > 0.05.

**
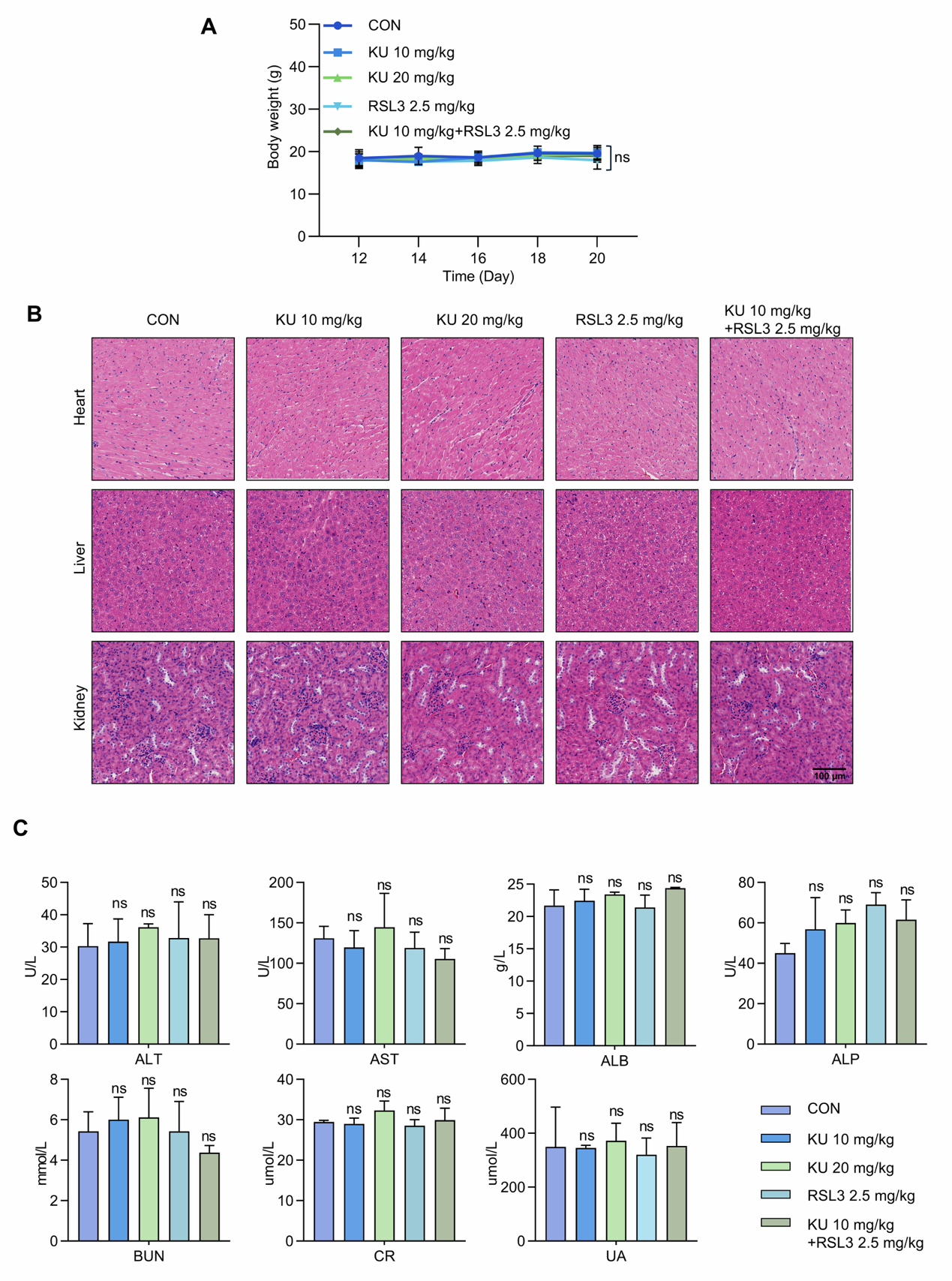
**

**Supplementary Fig. 18** **The drug toxicity results of KU-57788 and RSL3 *in vivo* A** The body weight was measured with KU-57788 (0, 10 and 20 mg/kg, intraperitoneal injection) and RSL3 (0 and 2.5 mg/kg, intraperitoneal injection) (n = 5/group) were analyzed by GraphPad Prism 9 software. Data are shown as mean ± SD. **B** Quantitative chart about ALT, AST, ALB, ALP, BUN, CR and UA of each group. **C and D** Liver, heart and kidney HE staining of each group. Values are presented as mean ± SD for n = 3, analyzed by one-way ANOVA using the Holm-Sidak method (B). **p* < 0. 05, ***p* < 0. 01, ns, *p* > 0.05.

**Supplementary Table**

The PCR primer sequences for human genes

| **Gene** | **Forward/Reverse** | **Sequences (5′-3′)** |
| --- | --- | --- |
| *PRKDC* (DNA-PKcs) | Forward | CCTGGCCGGTCATCAACTG |
|  | Reverse | AGTAAGGTGCGATCTTCTGGC |
| *DNM1L* (DRP1) | Forward | TCACCCGGAGACCTCTCATTC |
|  | Reverse | GGTTCAGGGCTTACTCCCTTAT |
| *SLC7A11* | Forward | GCGTGGGCATGTCTCTGAC |
|  | Reverse | GCTGGTAATGGACCAAAGACTTC |
| *SLC3A2* | Forward | CTGGTGCCGTGGTCATAATC |
|  | Reverse | GCTCAGGTAATCGAGACGCC |
| *GCLC* | Forward | GGCACAAGGACGTTCTCAAGT |
|  | Reverse | CAGACAGGACCAACCGGAC |
| *GCLM* | Forward | TGTCTTGGAATGCACTGTATCTC |
|  | Reverse | CCCAGTAAGGCTGTAAATGCTC |
| *NFE2L2* (NRF2) | Forward | TCCAGTCAGAAACCAGTGGAT |
|  | Reverse | GAATGTCTGCGCCAAAAGCTG |
| *18S rRNA gene* | Forward | AGGCCCTGTAATTGGAATGAGTC |
|  | Reverse | GCTCCCAAGATCCAACTACGAG |
